# Evaluation of Tendon Fastener Designed for Percutaneous Placement in an Ovine Model for Repair of Articular-Sided Rotator Cuff Tears

**DOI:** 10.3390/jfb17100526

**Published:** 2026-10-17

**Authors:** Mark Miller, Adam Kohring, Tiffany Bridges, Benjamin C. Gadomski, Kevin Labus, Daniel Regan, Jeremiah Easley, Drew W. Koch, Ashley Potter, William B. Morrison

**Affiliations:** 1 Department of Orthopedic Surgery, Jefferson Health New Jersey, Stratford, NJ 08084, USA; 2 Department of Veterinary Medicine, Colorado State University College of Veterinary Medicine & Biomedical Sciences, Fort Collins, CO 80523, USAdrew.koch@colostate.edu (D.W.K.);; 3 Department of Radiology, Jefferson Health, Philadelphia, PA 19107, USA; william.morrison@jefferson.edu

**Keywords:** rotator cuff tears, tendon healing, augmented repair

## Abstract

**Introduction**: Surgical treatment for partial-thickness rotator cuff tendon (RCT) tears often involves open or arthroscopic conversion to a full-thickness tear. A novel method has been developed that utilizes sutureless bone-to-tendon fixation using a percutaneous *fastener*. **Purpose**: This study characterizes the biomechanical stability of the *fastener* in a partial-tear ovine rotator cuff model compared to that of a screw-and-washer. **Methods**: The study included an ex vivo cadaveric phase and an in vivo pilot. During the ex vivo phase, the infraspinatus tendon was partially transected in 20 cadaveric sheep shoulders. Shoulders were allocated into three groups: *fastener* (FS) (n = 10), screw-and-washer (S/W) (n = 7), and an empty group without fixation (n = 3). In an in vivo pilot, three sheep underwent bilateral partial infraspinatus transection and repair, with the *fastener* in one shoulder and the screw-and-washer in the contralateral shoulder; animals were euthanized at 8 weeks for histological analysis. Both cohorts underwent biomechanical assessments, and the in vivo samples underwent histological dissection and analysis. **Results**: No significant biomechanical differences were noted among the three groups in ultimate load (FS: 2822 N, S/W: 2599 N, empty: 2924 N), ultimate displacement (FS: 15.9 mm, S/W: 15.5 mm, empty: 13.8 mm), stiffness (FS: 252.4 N/mm, S/W: 237.7 N/mm, empty: 279.9 N/mm), cross-sectional area (FS: 90.0 mm^2^, S/W: 93.0 mm^2^, empty: 78.8 mm^2^), ultimate stress (FS: 32.9 MPa, S/W: 28.3 MPa, empty: 37.8 MPa), ultimate strain (FS: 0.203 mm/mm, S/W: 0.183 mm/mm, empty: 0.183 mm/mm), or elastic modulus (FS: 231.1 MPa, S/W: 220.3 MPa, empty: 271.3 MPa). The mode of failure was most frequently a mid-substance tear. Both the *fastener* and the screw-and-washer demonstrated comparable histology across the graded parameters regardless of the evaluated animal or sample. **Conclusions**: The *fastener* offers similar biomechanical stability to that of a screw-and-washer construct in the treatment of partial rotator cuff tears in an ovine model. **Clinical Relevance**: The results of this animal model study provide evidence to consider further clinical testing.

## 1. Introduction

Traumatic and degenerative tears of the rotator cuff are among the most common musculoskeletal pathologies, affecting between 6.8% and 22.4% of adults over 40 years of age in the United States and resulting in significant economic and logistical burdens placed on healthcare systems [1]. As understanding and imaging modalities have improved, the rate of partial rotator cuff tears has continued to increase over time [1]. Conservative management of PTRCT with less than 50% tendon involvement is preferred but is a multifactorial decision that can be affected by the patients’ age, symptoms, functional deficit, size and location of the tear, nature of onset, and etiology [2].

Nonoperative therapies include physical therapy, exercise, activity modification, and anti-inflammatory medications. Patients who have exhausted conservative management may benefit from surgical repair, which can involve arthroscopic debridement, conversion, in situ, all-intra-articular, and transosseous repair [1]. Biomechanical studies of articular-sided tears have demonstrated that in situ fixation has the greatest strength when compared with completion of the tear and subsequent repair [3,4]. Despite these findings, there does not appear to be a clinical advantage to conversion repair when compared to in situ repair [5].

Although operative management of in situ articular-sided rotator cuff tendon (ASRCT) tears has proven successful, these repairs are not without a cost burden to the healthcare system. One study found that the total healthcare cost for management and surgical treatment of partial rotator cuff injury was over thirty-four thousand dollars [6]. Patients are typically required to exhaust conservative options before electing surgical treatment, despite the increased risk of further injury to the rotator cuff that can occur in the interim, thus leading to increased patient morbidity and increased cost to the healthcare system [7,8,9]. One solution to decrease cost would be to perform these types of repairs in the outpatient setting under minimal sedation, which has been previously described in the Wide-Awake, Local Anesthesia, No Tourniquet (WALANT) model frequently utilized in distal upper extremity surgery and demonstrated to lower overall costs [10,11].

Given the aforementioned literature, there exists a need within the healthcare system to address rotator cuff tears through minimally invasive methods. A novel method to address articular-sided rotator cuff tears in a minimally invasive fashion is a sutureless, bone-to-tendon fixation device using the *fastener* [Trace Orthopedics LLC]. The *fastener* is designed to be placed percutaneously through a needle under ultrasound guidance, without the need for general anesthesia. To our knowledge, the utilization of the *Fastener* device for repair of ASRCT tears has not been previously described. The objective of the present study was to characterize the biomechanical stability of the *fastener (FS)* in a partial-tear ovine rotator-cuff model, compared to a commercially available screw-and-washer (S/W) construct, alongside an in vivo pilot study to assess bony healing potential.

## 2. Methods

### 2.1. Overview

The ex vivo and in vivo portions of the study were performed in parallel. The ex vivo cadaveric phase involved explanted ovine shoulders. Twenty shoulders (a combination of left and right) were collected during necropsy from 20 different sheep involved in studies unrelated to the shoulder, with one shoulder used per animal. Cadavers underwent a partial articular-sided infraspinatus transection involving less than 50% of the width of the tendon. Shoulders were divided into three groups: 10 shoulders were implanted with the *FS*, seven were implanted with a commercially available screw-and-washer device, and three shoulders were left untreated to serve as controls. All underwent biomechanical testing, radiography, and histological testing.

The in vivo phase involved three healthy sheep, with both shoulders utilized for comparison. In each animal, the left shoulder was implanted with the commercially available S/W device, while the right shoulder was implanted with the *FS* device. Side allocation was fixed for all animals to standardize surgical technique and positioning. Bilateral acute articular-sided partial transection (50% of the width of the tendon) and re-attachment of the infraspinatus tendon were performed, with either the *fastener* or the screw-and-washer. After the implantation, the animals survived until 8 weeks (+/− 4 days). The three subjects were humanely euthanized and submitted to necropsy for gross evaluation. Analyses included radiographic, µCT and histological analysis.

### 2.2. Animal Information

An ovine model was utilized for this study because of the comparable anatomy and physiology of the ovine infraspinatus tendon and the rotator cuff of the human. This is an accepted translational model of chronic cuff injury followed by acute rotator cuff repair [12,13,14,15,16,17]. The study complied with the Animal Welfare Act (7U.S.C§§2131et. seq) and the Assurance Statement with the Office of Laboratory Animal Welfare at the National Institutes of Health (A3572-01). The animal facility also maintained accreditation by AAALAC. The Preclinical Surgical Research Laboratory (PSRL) at Colorado State University was committed to complying with all applicable regulations governing the care and use of laboratory animals. Animal care and use procedures in this protocol were approved by the Colorado State University Institutional Animal Care and Use Committee (IACUC) before animal receipt or transfer.

### 2.3. Ex Vivo Testing

#### 2.3.1. Destructive Biomechanical Testing

**Figure 1:**
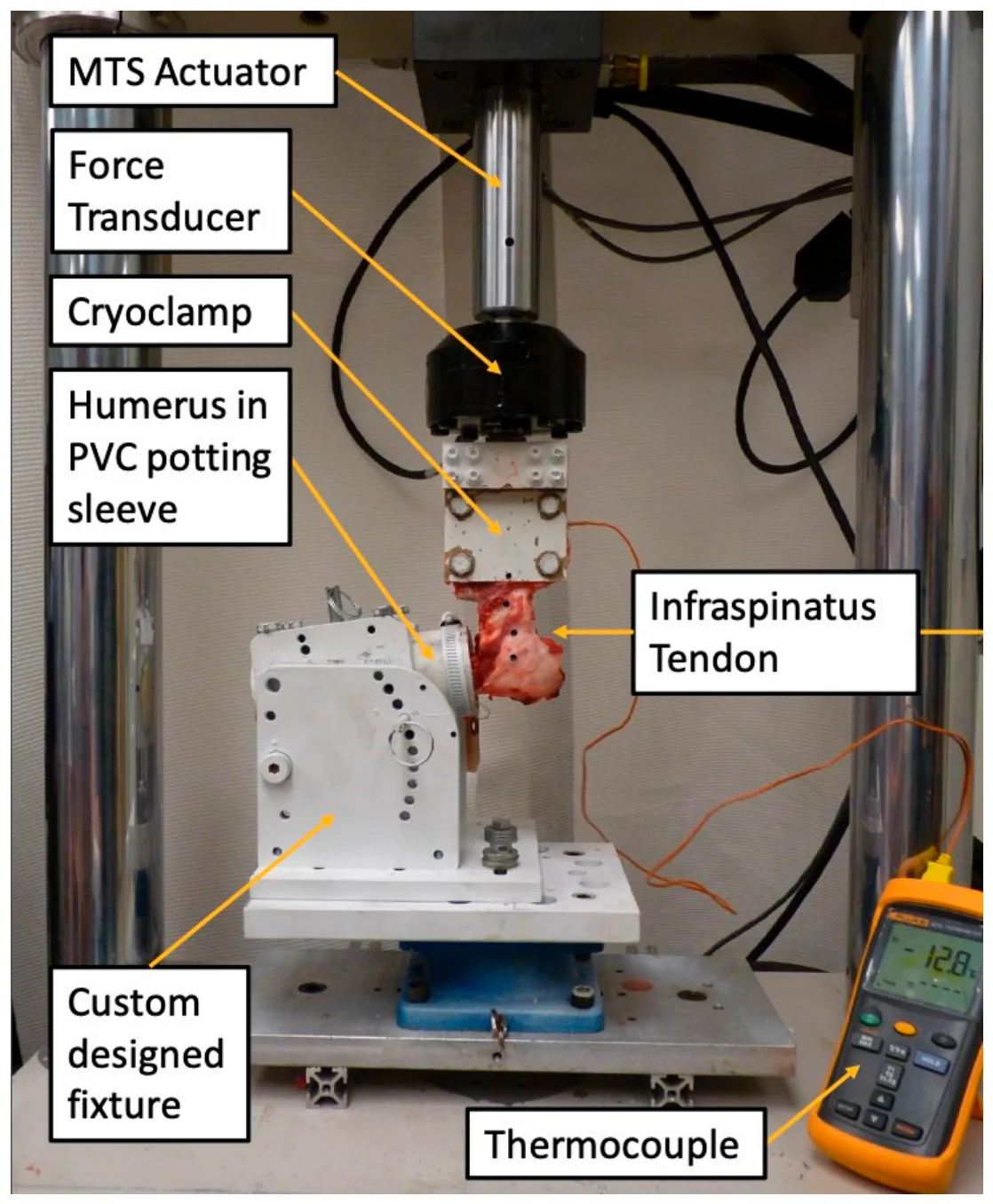
Ovine infraspinatus tendon–humerus bone construct mounted in the MTS testing system prior to biomechanical assessment. Features of interest have been highlighted, including the cryo-clamp.

In the ex vivo specimens, the *fastener* and screw-and-washer were implanted under direct visualization. Destructive biomechanical analyses were performed to quantify changes in the mechanical characteristics of the twenty ovine cadaver shoulder specimens. Sample hydration was maintained via physiologic saline spray at approximately ten-minute intervals during the entire preparation and testing protocol. Following dissection, the cross-sectional area of each tendon was measured by applying a uniform pressure of 0.12 MPa to a custom fixture that fully confines the tendon. The humeri were potted in a strong two-part hard-casting resin (SmoothCast 321, Smooth-On, Macungie, PA, USA) within a cylindrical polymer sleeve to ensure proper mechanical fixation between the sample and the testing system. The potted section of each sample was rigidly fixed to the frame of a servo-hydraulic material testing system (MiniBionix 858, MTS, Eden Prairie, MN, USA), such that the infraspinatus tendon physiological loading direction was aligned axially with the actuator of the material testing machine (Figure 1). The tendon was gripped in a custom cryo-clamp rigidly attached to a force transducer and the actuator. The internal clamping temperature was maintained at a maximum of −10 °C throughout the test to prevent the tendon from slipping in the grip. The temperature was confirmed using a thermocouple probe within the grip.

**Figure 2:**
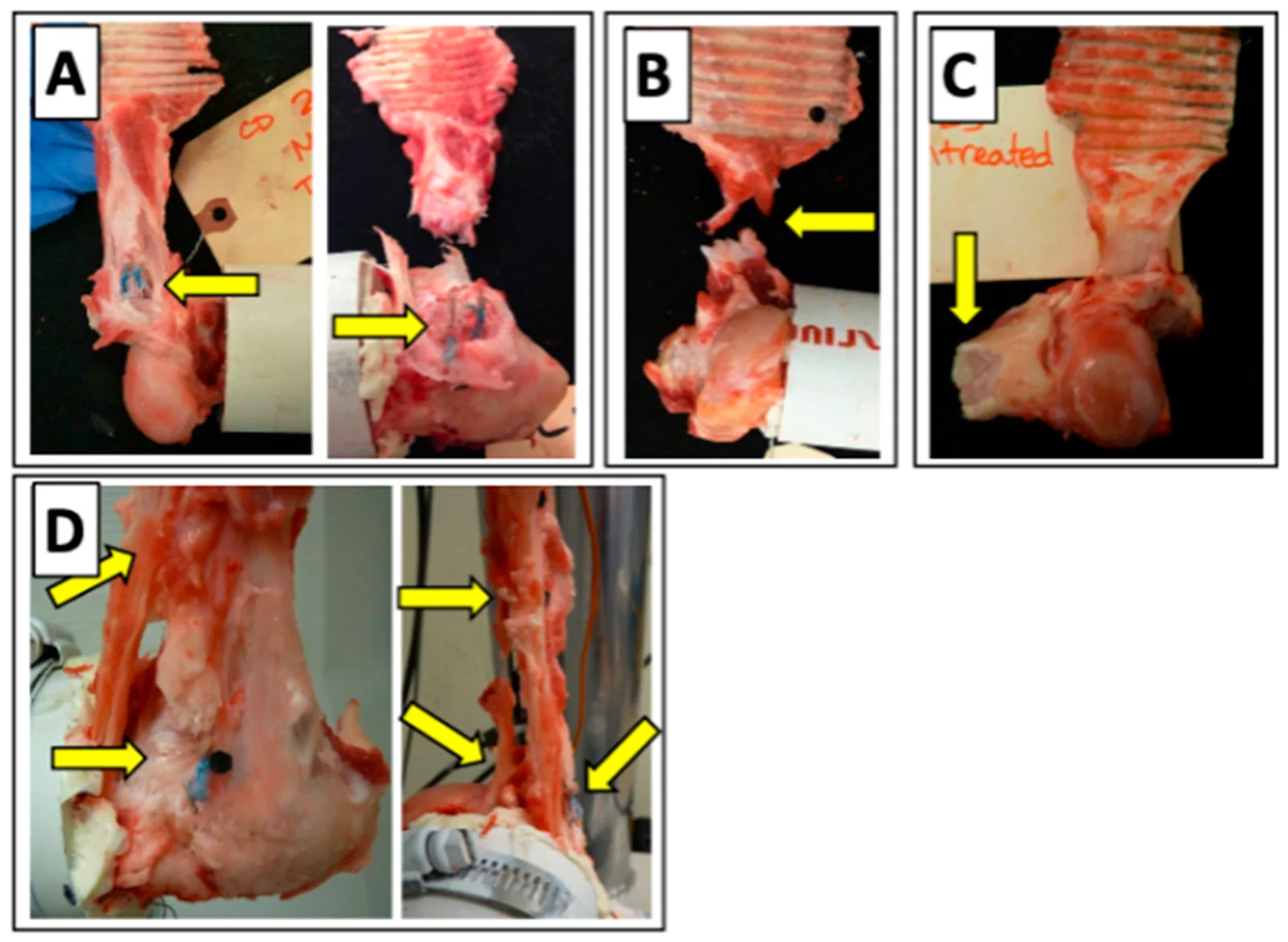
Digital images representing the expected modes of failure that may be observed following destructive ramp-to-failure testing. Yellow arrows indicate the location(s) of the failure in each image. Suture material at the repair site can be seen in inserts (**A**,**D**). ((**A**) Failure at repair site, (**B**) mid-substance failure, (**C**) bone avulsion, and (**D**) failure at repair site + mid-substance failure (mixed failure mode)).

The biomechanical testing procedure consisted of an initial static pre-load, followed by preconditioning cycles and a stress-relaxation test. The 10 N pre-load was maintained for about 2 min or until the specimen was fully relaxed. Following the pre-load, the reference gauge length of the tendon was measured from the bone insertion to the grip using calipers. The measured gauge length was used to determine the applied displacement for preconditioning cycles and stress-relaxation testing. The samples underwent 10 cycles of preconditioning between 0% and 2% engineering strain. Following preconditioning, specimens were loaded in one ramp-to-failure test at 0.5% strain/sec. Digital photographs were taken of each specimen after failure to document the location and mode of failure (Figure 2).

#### 2.3.2. Structural Properties

Structural properties representing the mechanical behavior of the bone-tendon construct were calculated from the force and displacement data collected during the ramp-to-failure test. Ultimate load (N) is the maximum force during the test. Ultimate displacement (mm) is the displacement when the ultimate load was achieved during the test. Stiffness (N/mm) is the slope of the force-displacement curve taken in the linear portion before discernible yield.

#### 2.3.3. Mechanical Properties

Material properties were calculated by normalization of the structural properties to the cross-sectional area. Ultimate stress (MPa) was the maximum stress during the test. Ultimate strain (mm/mm) was the strain when ultimate stress was achieved during the test. Elastic modulus (MPa) was the slope of the stress–strain curve taken in the linear portion before discernible yield. Following destructive biomechanical testing, radiographs were taken of the tested specimens in the coronal plane. Following destructive biomechanical testing, radiographs and imaging, tested samples were discarded.

### 2.4. In Vivo Testing

For the in vivo phase, animals underwent a visual herd examination upon receipt at the laboratory by a veterinarian. Additionally, a follow-up examination was performed by a veterinarian or qualified staff before selection for this study. The animals selected were deemed free of any disease or condition that would interfere with the purpose or conduct of the study. The first three (3) animals from the pool of available animals coming down the chute that met study requirements were selected, and ear tags were assigned.

#### 2.4.1. Specimen Imaging and Dissection

For preparation of the shoulder joint, a 10 cm skin incision was made over the point of the shoulder joint, followed by a dissection of the medial subcutaneous coli in line with the incision and a medial deltoid split. Next, the infraspinatus tendon was isolated, inspected, and found to be intact, and approximately 50% of the infraspinatus was sharply released in the transverse plane along the articular edge at the humeral attachment. The injury was then repaired via an S/W or *FS* construct. The deltoid was then returned to its normal position and reattached to the brachial fascia. Sheep were monitored daily for general health, neurologic problems or lameness, and incision healing throughout the study period. All animals were humanely euthanized by an intravenous overdose of pentobarbitone sodium (88 mg/kg), per the American Veterinary Medical Association (AVMA) guidelines. All three animals were sacrificed 8 weeks (+/− 4 days) following surgery. Following euthanasia, a complete gross necropsy was performed. The right and left shoulders were carefully removed en bloc and labeled with the collection date, animal identification, and study identification.

Following gross necropsy, ovine forelimbs were delivered fresh to the Orthopaedic Bioengineering Research Laboratory (OBRL), accompanied by the necropsy record. Samples were dissected into humerus–infraspinatus tendon constructs (HTCs), and extraneous soft tissue was removed. Following fine dissection, digital images and radiographs were taken of all samples in the sagittal and coronal planes. All images included, at a minimum, study identification, sample identification, implant level, animal model type, and date.

#### 2.4.2. Histology Slide Preparation

Histological slides were prepared from the bilateral shoulders of the three in vivo ovine specimens. The samples allocated for histology were grossly sectioned into final tissue slabs, imaged, and then preserved in 10% neutral buffered formalin. Following full fixation, samples were dehydrated and cleared on a tissue processor. Samples were then infiltrated and polymerized into a hardened block with Acrylosin Hard solution (Dorn and Hart Microedge Inc, Loxley, AL, USA). A minimum of n = 2 slides per sample were cut in the sagittal plane, including the humeral enthesis, infraspinatus tendon, repair site, and test article, resulting in a total of at least 18 slides (N = 9 shoulders × 1 (Region of Interest (ROI)/shoulder) × 2 (slides/ROI)). Slides were made using the Exakt method (Exakt Technologies, Oklahoma City, OK, USA). Slides were ground to a final thickness of approximately 50–70 microns, polished, and stained with Sanderson’s Rapid Bone Stain and counter-stained with Van Gieson stain. Calibrated digital images at 10× overall magnification were captured for each slide (ImagePro, Media Cybernetic, Silver Spring, MD, USA). Stained histology sections were evaluated by the study pathologist, who was initially blinded to the treatment parameters. Sanderson’s Rapid Bone Stained and counterstained with Van Gieson stain slides from the humerus and infraspinatus tendons were analyzed and graded according to cell type and responses, following the grading scheme and references included in Table 1. The equipment used included the Olympus BX43 microscope, Olympus DP73 camera, and Olympus CellSens software.

### 2.5. Statistical Analysis

Statistical comparisons on continuous data (biomechanics) were made using a standard one-way ANOVA, with an alpha value (α) of 0.05 and a Tukey post hoc test where appropriate.

## 3. Results

### 3.1. Destructive Biomechanical Testing Results

**Figure 3:**
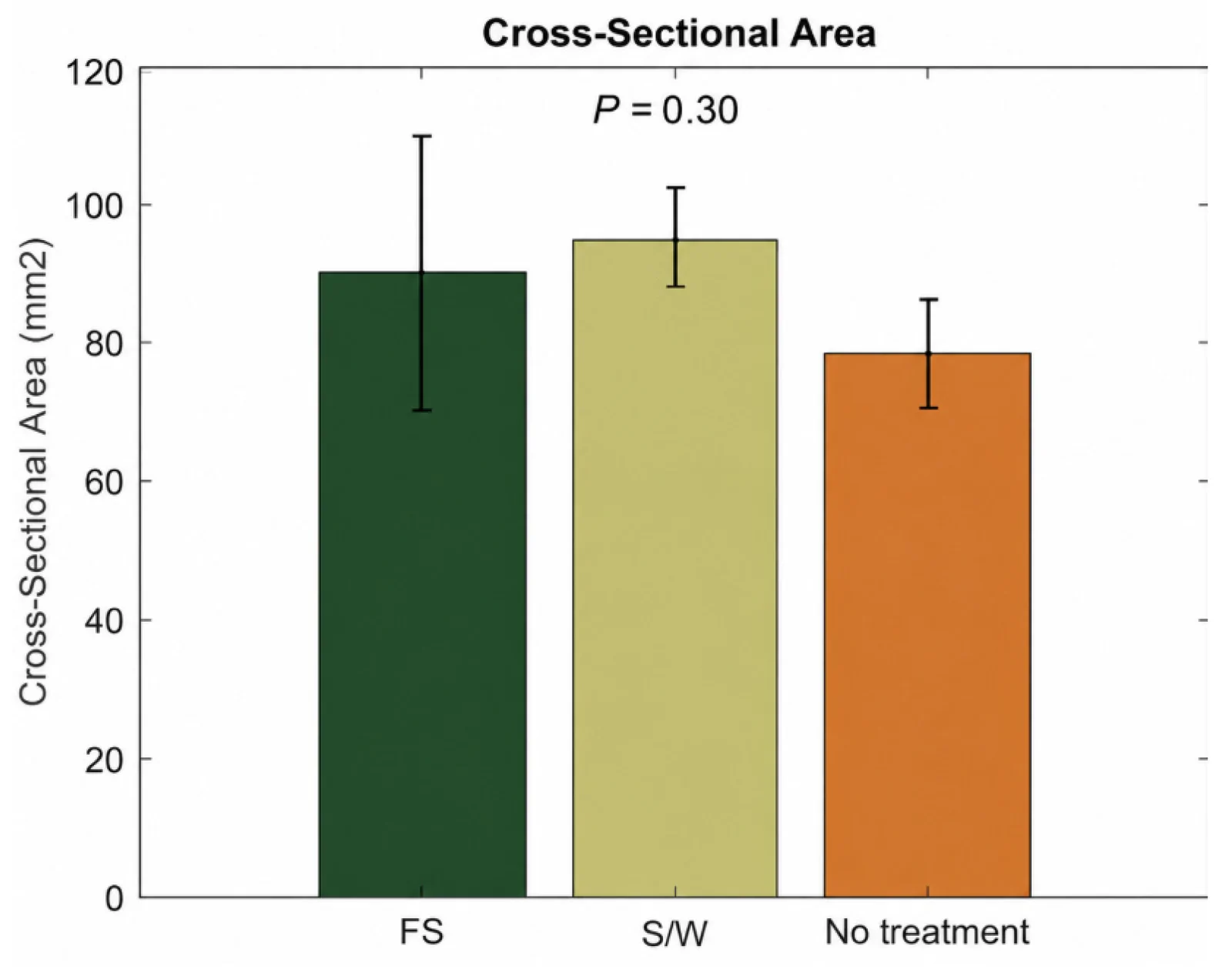
Biomechanical testing of tendon cross-sectional area between groups.

**Figure 4:**
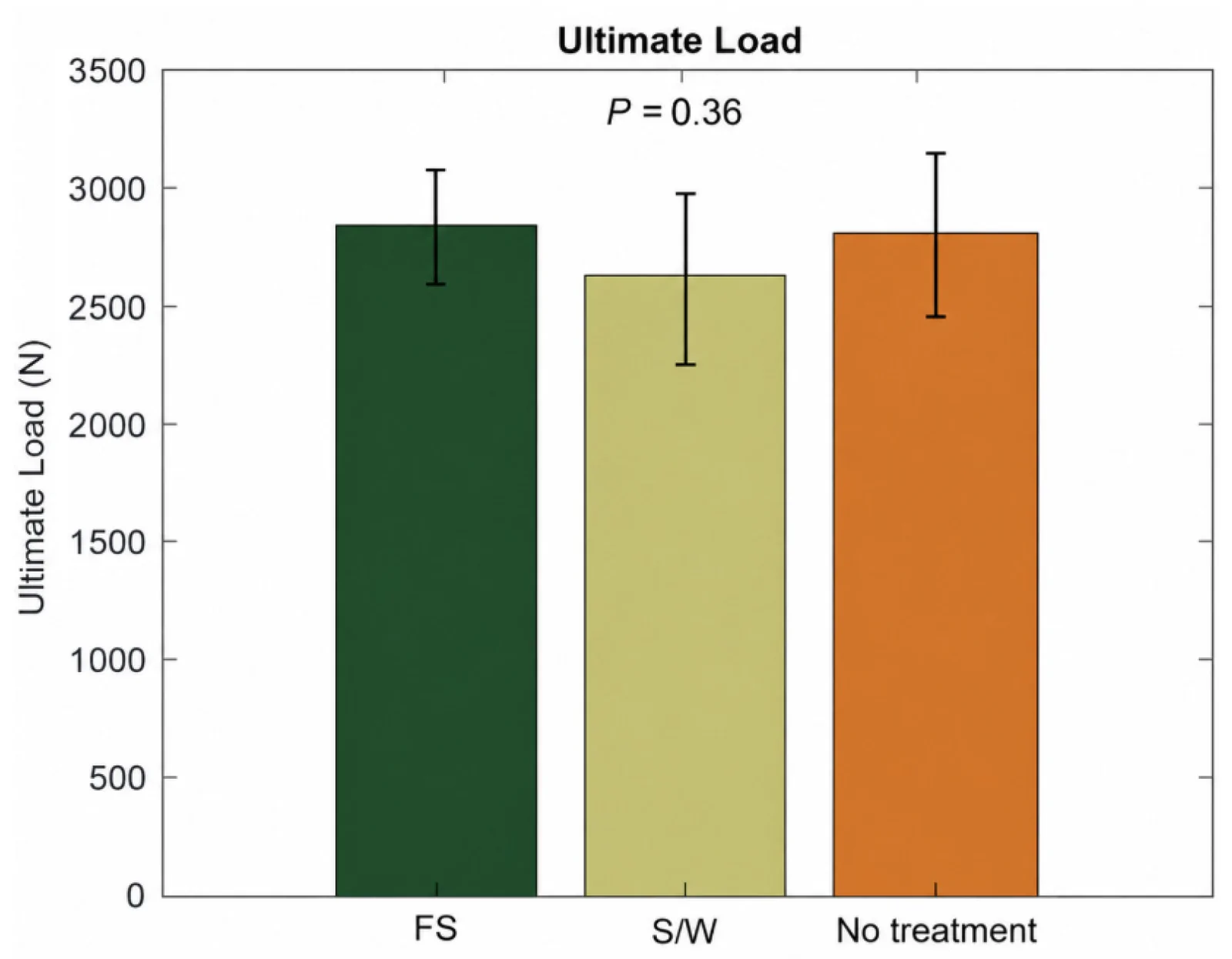
Biomechanical testing of tendon ultimate load.

**Figure 5:**
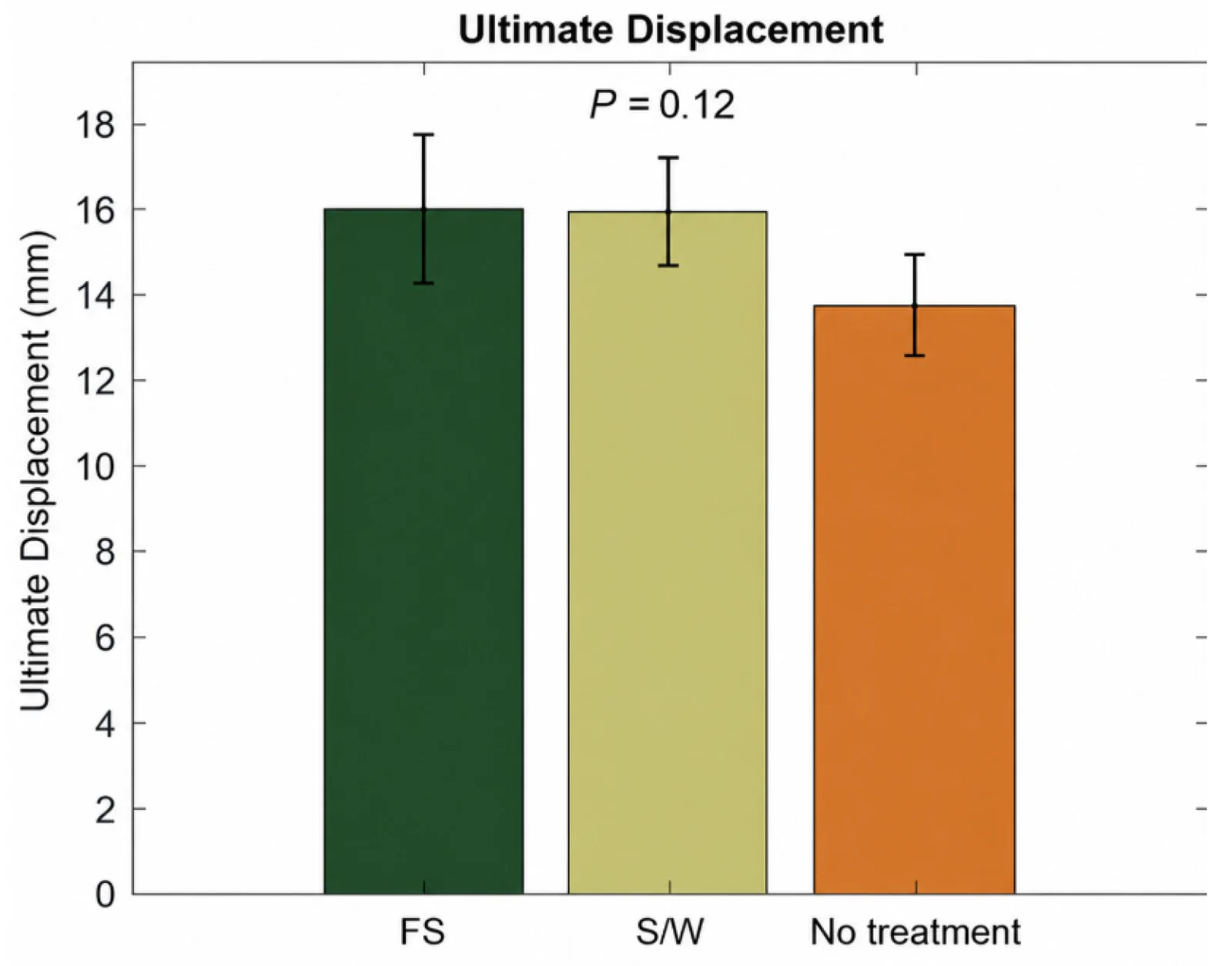
Biomechanical testing of tendon ultimate displacement.

**Figure 6:**
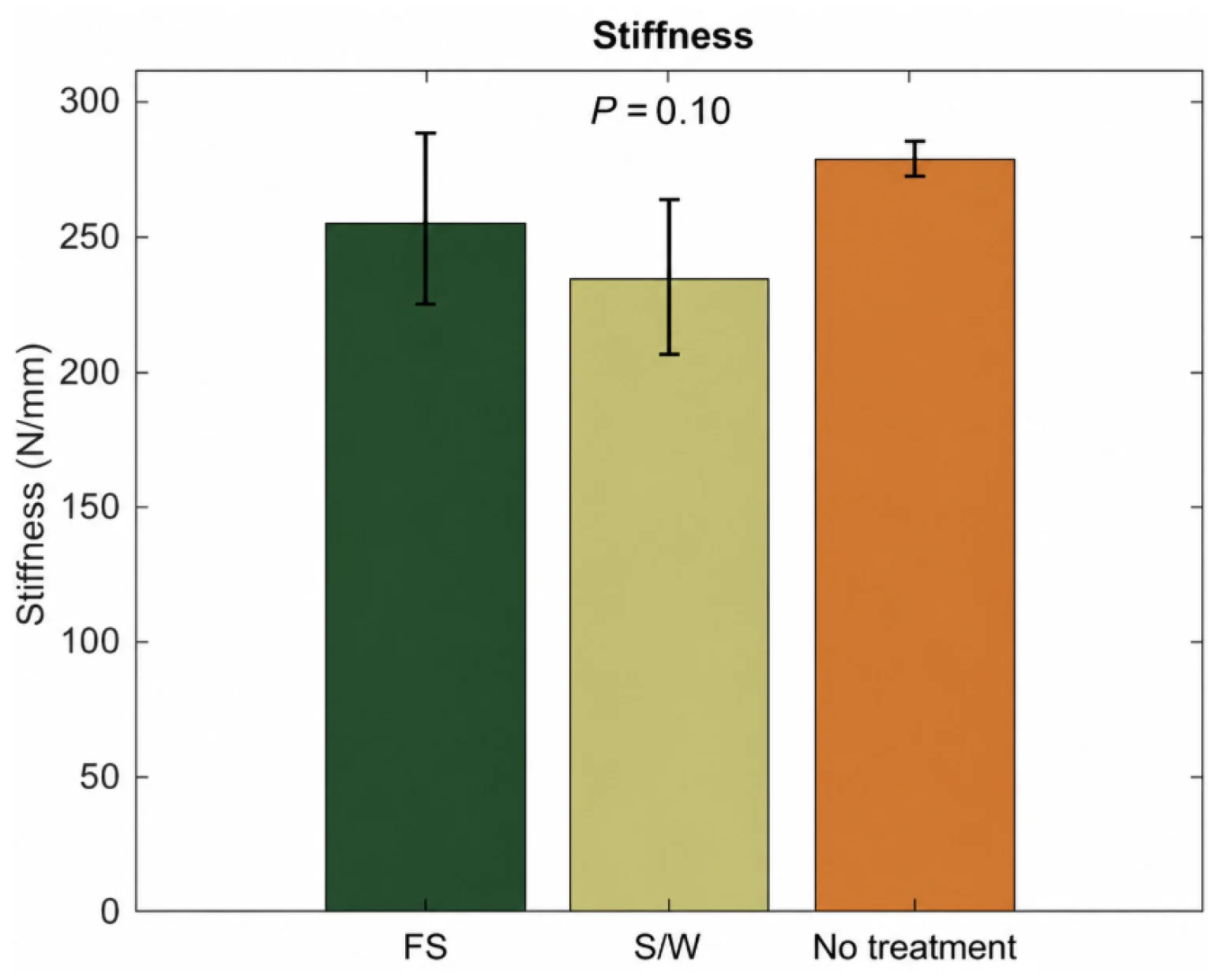
Biomechanical testing of tendon stiffness.

**Figure 7:**
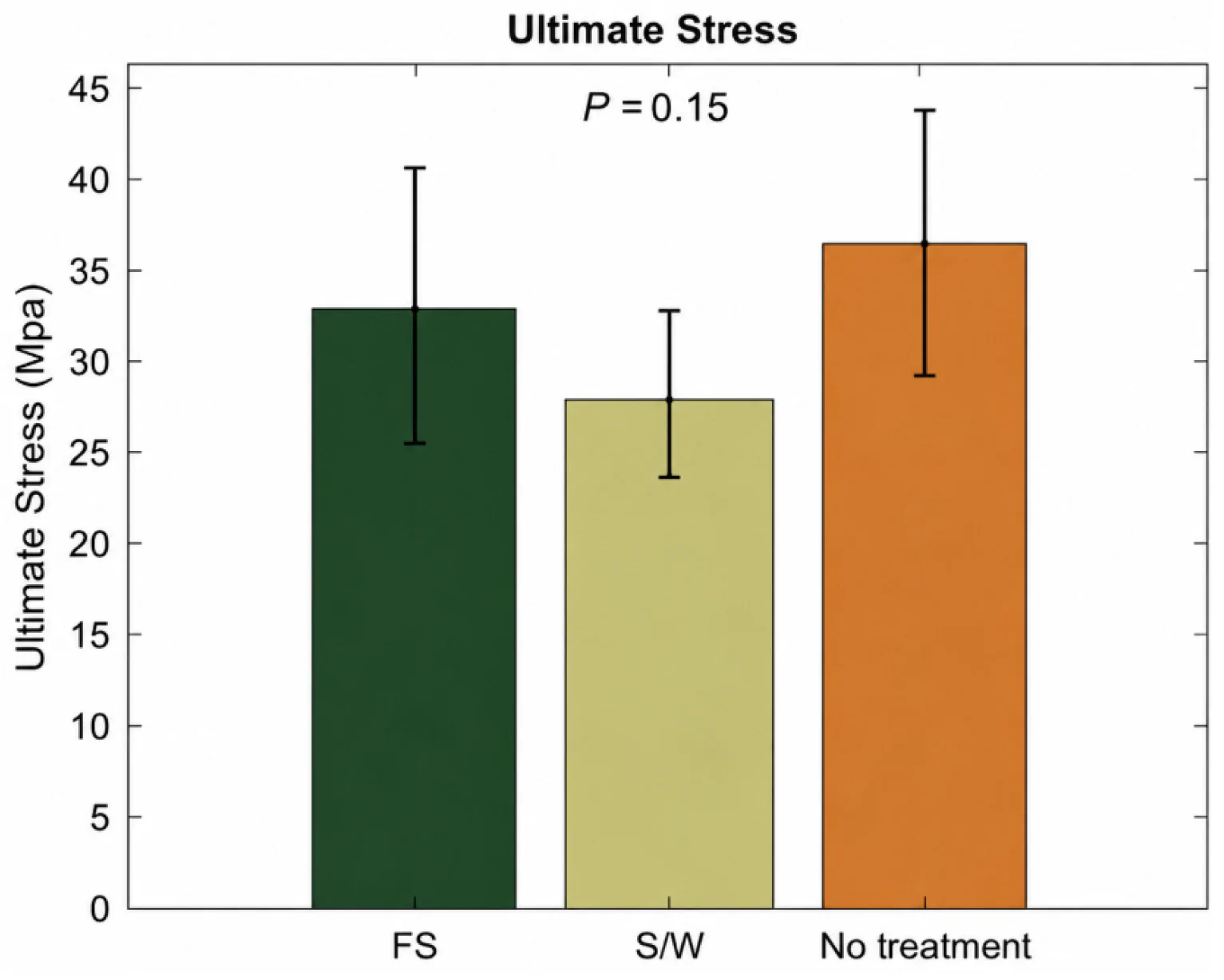
Biomechanical testing of tendon ultimate stress.

**Figure 8:**
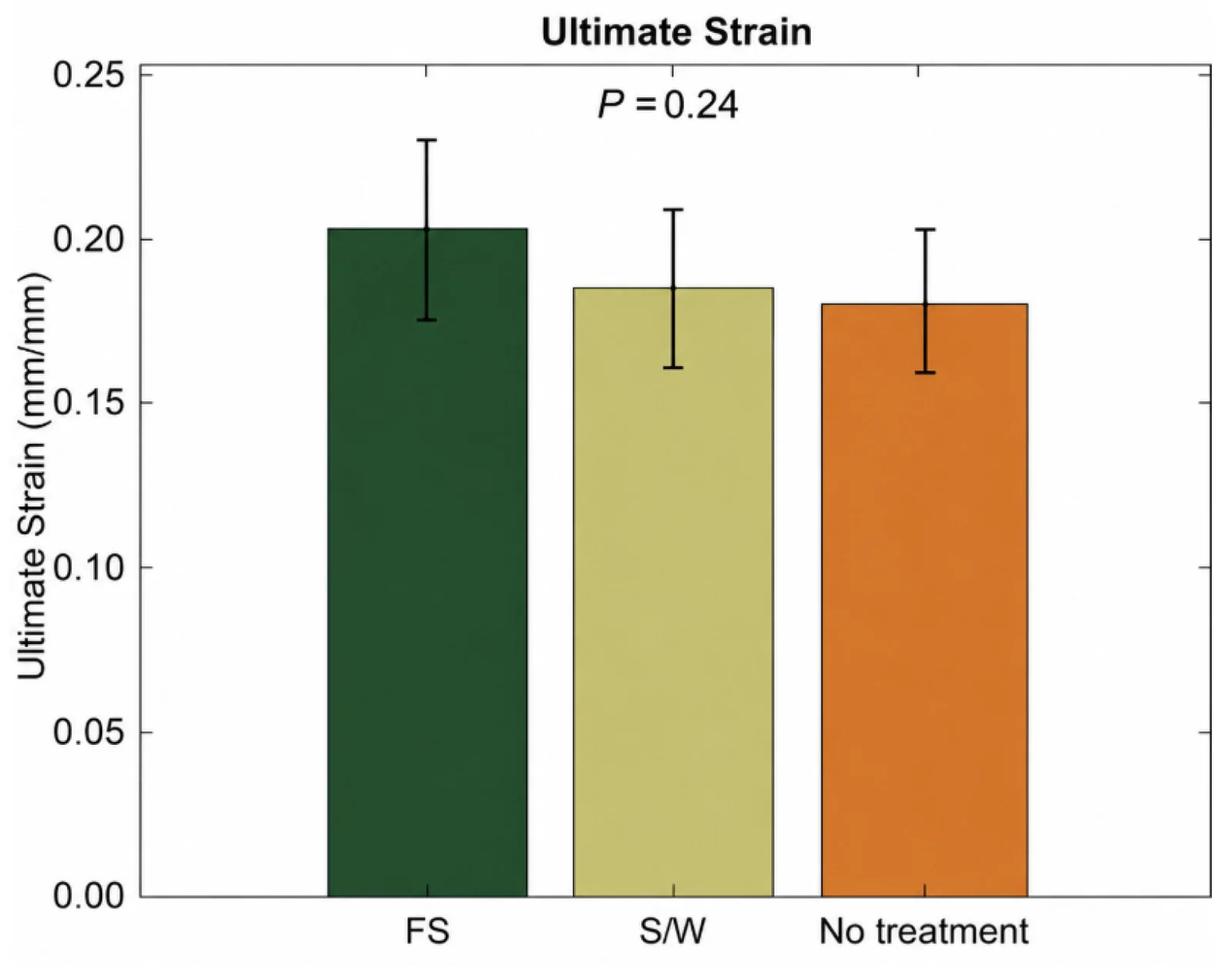
Biomechanical testing of tendon ultimate strain.

**Figure 9:**
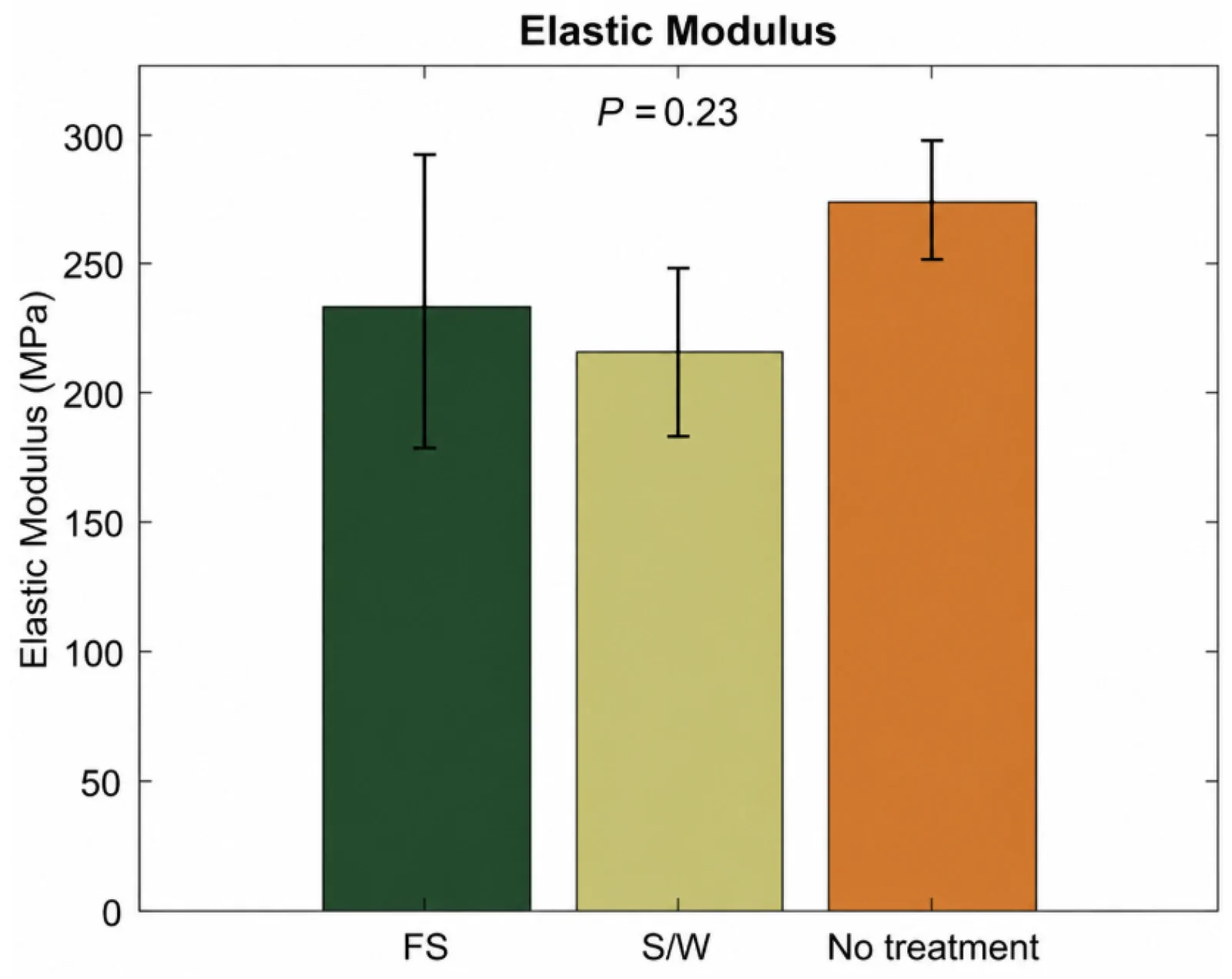
Biomechanical testing of tendon elastic modulus.

No significant differences were noted between the *fastener* (n = 10), screw-and-washer (n = 7), and empty (n = 3) groups in any biomechanical outcome parameter: cross-sectional area (*p* = 0.30), ultimate load (*p* = 0.36), ultimate displacement (*p* = 0.12), stiffness (*p* = 0.10), ultimate stress (*p* = 0.15), ultimate strain (*p* = 0.24), or elastic modulus (*p* = 0.23) (Table 2; Figure 3, Figure 4, Figure 5, Figure 6, Figure 7, Figure 8 and Figure 9). No specimen in either repair group failed at the repair site. Mid-substance tendon failure was the predominant mode of failure in all groups (*fastener* 7/10; screw-and-washer 5/7; empty 2/3); the remaining specimens failed in the tendon near the grip (*fastener* 3/10; screw-and-washer 2/7) or by bone avulsion (empty 1/3). One-way ANOVA testing was performed for cross-sectional area, load, displacement, stiffness, stress, strain, and elastic modulus, which demonstrated that there was no statistical difference among the *fastener*, screw-and-washer, or untreated groups.

### 3.2. Histological Testing Results

**Figure 10:**
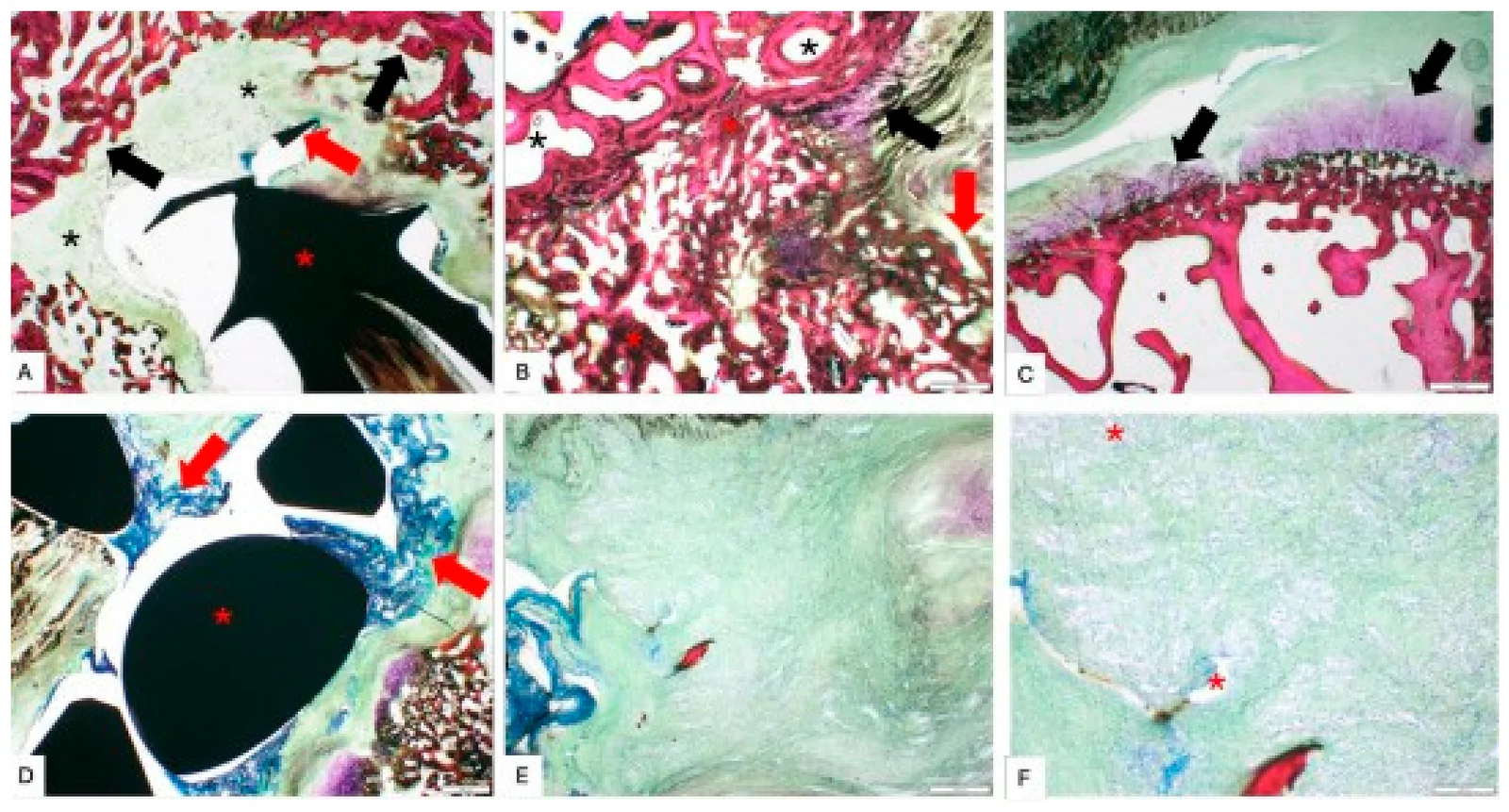
The 8-week timepoint, TOR01 left shoulder, screw-and-washer. (**A**) 2×; a sub-gross view of the screw-and-washer (red asterisk) associated with host bone (black arrow). Note the large cavitated space between the screw-and-washer and host bone that is filled with a cellular mesenchymal population (fibroplasia, black asterisk). The screw-and-washer is fragmented (red arrow). (**B**) 2×; a sub-gross view of mature cortical bone (black asterisk) and immature bony outcropping, enthesophyte (red asterisk), with variable degrees of fibrocartilaginous enthesis (black arrow) and fibrous enthesis attachment (red arrow). (**C**) 2×; sub-gross view of fibrocartilaginous enthesis attachment with well-delineated and mature cartilage anchoring tendon fibers to the humeral footprint (black arrow). (**D**) 4×; the head of the screw-and-washer (red asterisk). Surrounding the head of the screw-and-washer is a moderate amount of blue matrix (red arrow), which may be representative of foreign material. (**E**,**F**) 10 and 20×; surrounding the screw-and-washer are regions of increased tenocyte cellularity and disorganization of the collagenous fibers. Scattered through this cellular matrix are increased numbers of vascular profiles (red asterisk). Scale bars represent 50 µm (micrometers).

**Figure 11:**
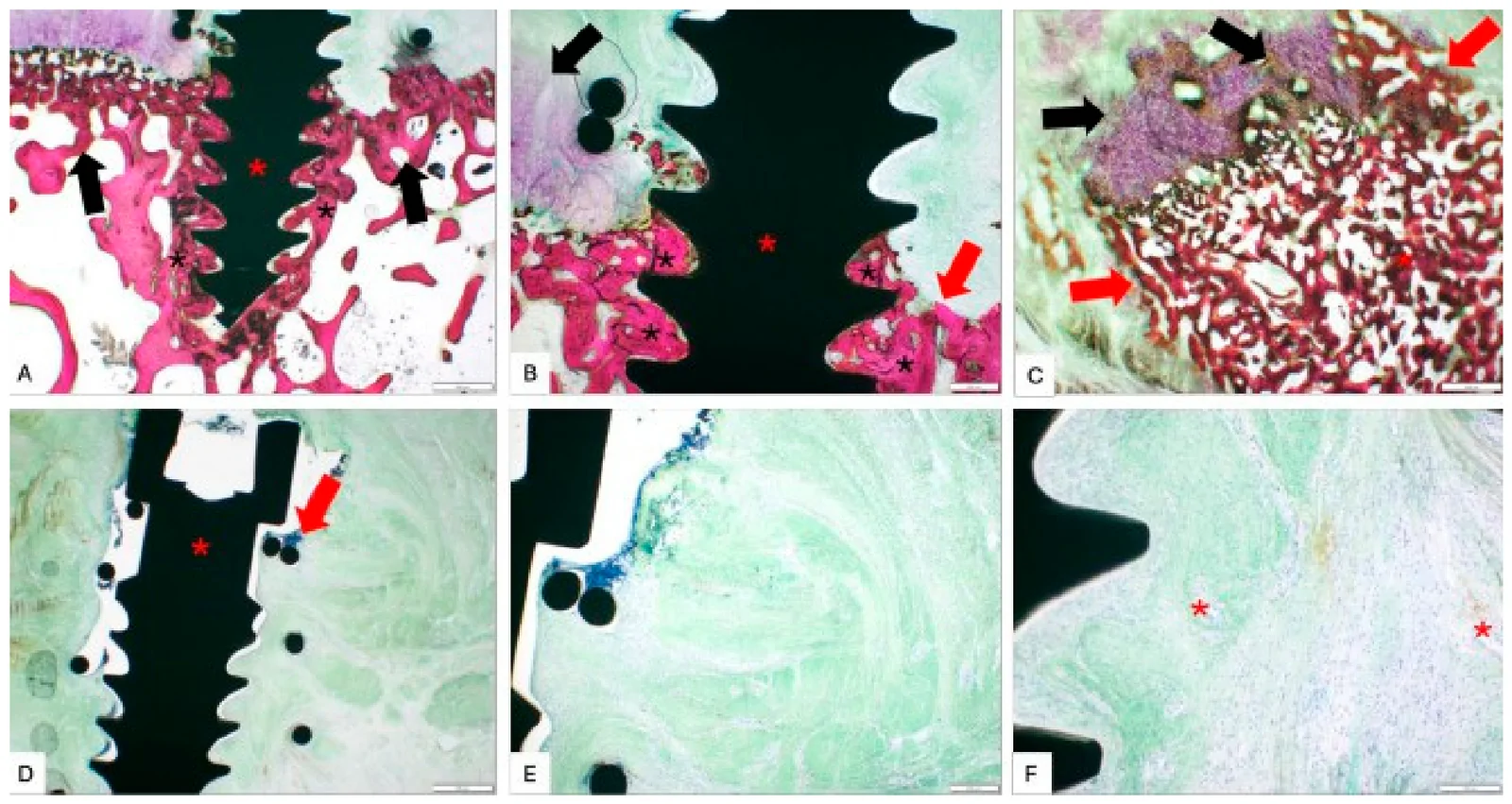
The 8-week timepoint, TOR01 right shoulder, fastener. (**A**) 2×; a sub-gross view of the fastener (red asterisk) associated with host bone (black arrow). Note the close apposition of the bone with the fastener and the bony sclerosis around the fastener region (black asterisk). (**B**) 4×; higher magnification demonstrating the intercalation of the fastener (red asterisk) with host bone (black asterisk). There is evidence of both fibrocartilaginous (black arrow) and fibrous enthesis attachment (red arrow). (**C**) 2×; a sub-gross view of an immature bony outcropping, enthesophyte (red asterisk), with variable degrees of fibrocartilaginous enthesis (black arrow) and fibrous enthesis attachment (red arrow). (**D**) 2×; the head of the fastener (red asterisk). Surrounding the head of the fastener is a scant amount of blue matrix (red arrow), which may be representative of foreign material. (**E**,**F**) 4 and 10×; surrounding the fastener are regions of increased tenocyte cellularity and disorganization of the collagenous fibers. Scattered through this cellular matrix are increased numbers of vascular profiles (red asterisk). Scale bars represent 50 µm (micrometers).

Both the *fastener* and screw-and-washer groups demonstrated comparable histology across the graded parameters regardless of the evaluated animal or sample. The type of sample preparation, ground tissue preparation, inhibited thorough evaluation of several key parameters of rotator-cuff repair such as corresponding inflammation and subtle bony remodeling such as active osteoblasts and aggregating osteoclasts, with both *fastener* and screw-and-washer groups demonstrating similar enthesis formation with the majority of samples having a portion of enthesis attachment characterized by direct fibrous attachment to the bone (<50%). There were several key differences in the parameters between *fastener* and screw-and-washer. Screw-and-washer tissues had increased collagenous disorganization and immaturity with increased tenocyte cellularity compared to the treatment group (Figure 10). This contrasts with the *fastener* tissues (Figure 11), which had apposition to host bone, with little to no cavity formation. This lack of cavity and close apposition led to prominent bony sclerosis circumscribing the *fastener*. Overall, these changes lead the treatment tissues to have improved tendon fiber alignment, cellularity, and neovascularization scores compared to screw-and-washer tissues (Table 3 and Table 4, Figure 10 and Figure 11). There was no significant influx in cell response, including polymorphonuclear cells, lymphocytes, plasma cells, macrophages, signs of implant degradation, or particulate debris within any of the groups (Table 3 and Table 4). Given the small number of specimens per group, histological data are presented descriptively, and no statistical comparisons were performed.

## 4. Discussion

Orthopedic literature commonly utilizes patient-reported outcome measures (PROMs) to compare the effectiveness of various treatments to alleviate the symptoms of rotator cuff tears [18,19,20,21]. While these models are efficacious in ascertaining clinical differences in patient outcomes, these models lack knowledge of the biomechanical differences among *fastener,* screw-and-washer, and conservative therapy. In the present study, the use of a well-defined animal model, that of the sheep infraspinatus, may assist in providing information regarding the biomechanical properties of treatment constructs that can be assessed alongside the novel *fastener*, a screw-and-washer, as well as an untreated group, as demonstrated in the previous literature [22,23,24]. The technique utilized to implant the novel *fastener* device was performed utilizing percutaneous methods under local imaging guidance, without the need for general anesthesia or formal presentation to an operating room. To our knowledge, this is the first biomechanical study evaluating the *fastener* device for the percutaneous management of rotator cuff pathology. Previous literature has investigated the utilization of the Wide-Awake, Local Anesthesia, No Tourniquet (WALANT) technique for the repair of rotator cuff tears; however, to date, there are no known devices with the ability to repair partial-thickness rotator cuff tears without direct visualization via an open or arthroscopic surgical approach [11].

Notably, the untreated group demonstrated an ultimate load similar to those of the *fastener* and screw-and-washer groups (2793 N vs. 2822 N and 2608 N). Partial transection involved less than 50% of the tendon width; most specimens failed mid-substance, and none failed at the repair site. This test reflected the strength of the intact tendon rather than the isolated strength of the fixation. These results show that neither device compromised the bone–tendon construct, but they do not establish fixation strength. This distinction is clinically important, as recurrent tears most commonly occur within the first 90 days after surgery [25]. Future studies using cyclic loading in a complete detachment model are needed to determine whether the *fastener* provides sufficient early fixation to permit accelerated rehabilitation.

When analyzing histological data, the significance of these findings, including enthesis organization, was difficult to quantify at a single timepoint. Additional timepoints are needed to assess differences in long-term progression of healing and resolution of joint injury. Overall, the most significant change between the *fastener* and the screw-and-washer tissues was the apposition of the host bone to the implant. There was poor apposition of host bone to the screw-and-washer constructs. In contrast, the *fastener* groups showed appropriate bony apposition. Whether these differences influence long-term fixation stability, bone remodeling, or enthesis healing cannot be determined from a single 8-week timepoint. Additional timepoints would be pertinent for further evaluation of the long-term resolution and healing of these sites. Notably, no inflammatory reaction was identified on histopathological analysis. This finding is likely attributable to the timing of the analysis. Following implantation of a biomaterial, the acute inflammatory response is characterized by a neutrophilic infiltrate and typically resolves within one week, while the subsequent chronic inflammatory phase, characterized by a mononuclear infiltrate, generally resolves within two weeks for biocompatible materials. Because histological analysis was performed at 8 weeks, both phases had likely resolved. An early inflammatory response likely did occur, as it is a necessary precursor to normal healing, and the well-established bone–implant interface observed around the *fastener* suggests that this healing cascade progressed appropriately [26]. However, definitive conclusions regarding the early inflammatory response cannot be drawn, as no analysis was performed within the first several weeks after implantation. Additionally, the section preparation used in this study limits the identification of inflammatory cells, so the absence of these cells should be interpreted with caution.

The present study is not without limitations. In the in vivo pilot, side allocation was fixed (screw-and-washer left, *fastener* right) to standardize surgical positioning. Although the within-animal design controls for between-animal variation, a side-related effect cannot be excluded with only three animals. The present model utilized the acute detachment and re-attachment of the ovine infraspinatus to simulate a rotator cuff tear but does not simulate the natural pathophysiology of rotator cuff tears, which typically result from repetitive microtrauma [27]. Previous literature has described a more robust inflammatory response in the ovine model when compared to human studies, and the effect this may have on translation to the human tendon healing model is not well understood [28]. Despite this, the discussed method has been previously described as a model to screen suture anchors, scaffolds, and other biologics [29,30]. It is well established that the proper biological environment is essential for proper tendon healing [31]. The present study did not evaluate the endogenous biological response to the *fastener* due to the biomechanical nature of the investigation, and the results should be appreciated in this context. Additionally, the histology was evaluated by a pathologist, and inter-rater reliability of the semiquantitative scoring was not assessed. Additionally, this was a pilot study to investigate the biomechanical properties of the *fastener* device, and therefore, the overall sample size was small; no control samples were obtained for histological comparisons. It cannot support conclusions regarding the safety or efficacy of the *fastener*. Furthermore, the ground-section histology used in this study limited the assessment of inflammation and subtle bone remodeling. Future studies should include thin-section histology and immunohistochemistry.

This study demonstrated that the *fastener* had similar fixation strength to that of traditional screw-and-washers across every biomechanical measure tested. The *fastener* also had a good bone-implant interface with no inflammatory reaction seen at 8 weeks. Translation to human studies is needed to evaluate the efficacy and safety of the *fastener* device for ultrasound-guided percutaneous and minimally invasive repair of partial-thickness rotator cuff tears.

## Figures and Tables

**Table 1:** Scoring system for histological evaluation of tendon–bone sections for inflammatory cell type and response.

	Score				
Cell Type/Response	0	1	2	3	4
Polymorphonuclear cells (PMNs)	None	Rare, 1–5/HPF	5–10/HPF	Heavy infiltrate	Packed
Lymphocytes	None	Rare, 1–5/HPF	5–10/HPF	Heavy infiltrate	Packed
Plasma cells	None	Rare, 1–5/HPF	5–10/HPF	Heavy infiltrate	Packed
Macrophages (Mφ)	None	Rare, 1–5/HPF	5–10/HPF	Heavy infiltrate	Packed
Giant cells	None	Rare, 1–2/HPF	3–5/HPF	Heavy infiltrate	Sheets
Signs of implant degradation	None	Minimal	Mild	Moderate	Severe
Particulate debris	None	Minimal	Mild	Moderate	Severe
Collagen arrangement	Uneven new collagen distribution. Areas with poorly organized fibrosis/fibroplasia mixed with areas with loose, disorganized collagen and/or gaps.	Abundant and continuous poorly organized fibrosis/fibroplast within transected tendon. Nonparallel arrangement of collagen fibers.	Roughly equal amounts of poorly organized fibrosis/fibroplast and well-organized polarized collagen. Weak demarcation of fiber bundles where collagen is well-organized.	Minimal amounts of poorly organized fibrosis/fibroplast Most collagen is well organized with characteristic wavy pattern. Maintenance of overall bundle architecture.	Collagen arranged in tightly cohesive way, organized in demarcated parallel bundles with a smooth, dense homogeneous polarization pattern with normal crimping

**Table 2:** Biomechanical testing results.

Group	Sample ID	Ultimate Load (N)	Ultimate Displacement (mm)	Stiffness (N/mm)	Cross-Sectional Area (mm^2^)	Ultimate Stress (MPa)	Ultimate Strain (mm/mm)	Elastic Modulus (MPa)	Mode of Failure
***Fastener***	TOR-CO1	2901	16.9	220.5	80.1	36.2	0.250	186.5	Tendon near grip
	TOR-CO2	2909	16.4	274.0	86.5	33.6	0.198	262.2	Tendon mid-substance
	TOR-CO3	3022	17.0	237.8	102.5	29.5	0.188	209.9	Tendon near grip
	TOR-CO4	2915	15.9	225.7	92.0	31.7	0.179	218.1	Tendon mid-substance
	TOR-CO5	2609	13.8	262.9	66.4	39.3	0.212	257.0	Tendon mid-substance
	TOR-CO6	2766	17.4	264.1	68.4	40.4	0.226	296.8	Tendon mid-substance
	TOR-CO7	2747	11.7	310.0	82.5	33.3	0.159	277.5	Tendon mid-substance
	TOR-CO8	2432	18.2	212.8	118.8	20.5	0.241	135.4	Tendon near grip
	TOR-CO9	3300	15.8	288.6	75.6	43.7	0.193	313.2	Tendon mid-substance
	TOR-C10	2620	15.9	227.6	127.1	20.6	0.184	154.6	Tendon mid-substance
	**Mean**	**2822**	**15.9**	**252.4**	**90.0**	**32.9**	**0.203**	**231.1**	
	**S.D.**	**245**	**1.9**	**32.4**	**20.4**	**7.8**	**0.029**	**60.1**	
**Screw-and-Washer**	TOR-C11	2959	14.9	278.5	88.4	33.5	0.188	249.6	Tendon mid-substance
	TOR-C12	3064	17.0	250.9	103.5	29.6	0.203	202.2	Tendon mid-substance
	TOR-C13	2472	16.7	229.0	100.4	24.6	0.190	200.4	Tendon mid-substance
	TOR-C14	2642	15.6	239.1	88.8	29.8	0.189	221.6	Tendon mid-substance
	TOR-C15	2072	17.8	195.7	105.3	19.7	0.215	153.8	Tendon near grip
	TOR-C16	2780	14.9	227.9	90.0	30.9	0.156	242.3	Tendon near grip
	TOR-C17	2270	14.0	208.5	91.4	24.8	0.147	217.8	Tendon mid-substance
	**Mean**	**2608**	**15.8**	**232.8**	**95.4**	**27.6**	**0.184**	**212.5**	
	**S.D.**	**361**	**1.4**	**27.3**	**7.4**	**4.7**	**0.024**	**31.8**	
**Empty**	TOR-C18	2533	13.2	271.9	76.1	33.3	0.172	274.5	Tendon mid-substance
	TOR-C19	2659	12.7	281.3	86.5	30.7	0.164	251.4	Bone avulsion
	TOR-C20	3188	14.9	278.5	71.0	44.9	0.201	291.2	Tendon mid-substance
	**Mean**	**2793**	**13.6**	**277.2**	**77.9**	36.3	**0.179**	**272.4**	
	**S.D.**	**348**	**1.2**	**4.8**	**7.9**	**7.6**	**0.020**	**20.0**	

**Table 3:** Histopathology scores, rotator cuff repair, week 8.

Animal ID	Time Point (Weeks)	Site	Sum Inflamm Score	Implant Degrade	Particulate Debris	Collagen Arrange	Cellularity (Tenocyte Reactivity/Stromal Cell Proliferation)	Neovascularization	Enthesis Type	Presence of Sharpey-like Fibers	Cumulative Score Based on Site
**TOR01**	8	Left	0	1	1	1	1	2	1	0	7
**TOR01**	8	Right	0	0	0	2	2	1	2	0	7
**TOR02**	8	Left	0	0	1	1	1	2	2	1	8
**TOR02**	8	Right	0	0	0	2	2	1	2	0	7
**TOR03**	8	Left	0	0	0	1	1	2	2	1	7
**TOR03**	8	Right	0	0	0	1	1	1	2	0	5
**Cumulative score** **based on parameter**			0	1	2	8	8	9	11	2	

**Table 4:** Test article-to-control categorization summary; 8-week timepoint.

**Treatment article**			
**Animal ID**	**TOR01**	**TOR02**	**TOR03**
**Site**	**Right**	**Right**	**Right**
**Tx**	***Fastener***	***Fastener***	***Fastener***
**PMNs**	0	0	0
**Lymphocytes**	0	0	0
**Plasma cells**	0	0	0
**M**φ	0	0	0
**Giant cells**	0	0	0
**Cumulative inflammation score**	0	0	0
**Subtotal A (x2)**	0	0	0
**Neovasc.**	1	1	1
**Total score**	1	1	1
**Group Total**	3		
**Avg. score per animal**	3/3 = 1		
**Control article**			
**Animal ID**	**TOR01**	**TOR02**	**TOR03**
**Site**	**Left**	**Left**	**Left**
**Tx**	**Screw-and-washer**	**Screw-and-washer**	**Screw-and-washer**
**PMNs**	0	0	0
**Lymphocytes**	0	0	0
**Plasma cells**	0	0	0
**M**φ	0	0	0
**Giant cells**	0	0	0
**Cumulative inflammation score**	0	0	0
**Subtotal A (x2)**	0	0	0
**Neovasc.**	2	2	2
**Total score**	2	2	2
**Group Total**	6		
**Avg. score per animal**	6/3 = 2		
**Test Article Score (Test score—Control score)**	1–2 = −1.0		
**Ranking**	Non-irritant (0.0–2.9)		

## Data Availability

The original contributions presented in this study are included in the article and Supplementary Materials. Further inquiries can be directed to the corresponding author.

## Supplementary Materials

The following supporting information can be downloaded at: https://www.mdpi.com/article/10.3390/jfb17100526/s1.
