# Supplementary material for: Evaluation of Tendon Fastener Designed for Percutaneous Placement in an Ovine Model for Repair of Articular-Sided Rotator Cuff Tears
Source: J Funct Biomater. 2026 Oct 17;17(10):526. doi: 10.3390/jfb17100526 (PMC13705963; doi:10.3390/jfb17100526)
Supplement: Supplementary file 1 [file jfb-17-00526-s001.zip › jfb-4500450-supplementary.pdf]

<table>
<tr><th>Specimen Info</th><th>Dorsal</th><th>Lateral</th></tr>
<tr><td rowspan="5">Sample ID:
TOR-CO1

Group:
Treatment

Timepoint:
Baseline</td><td>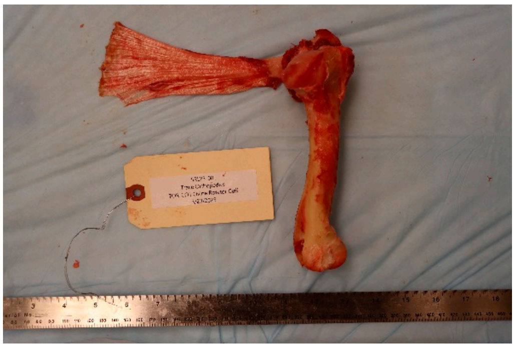</td><td>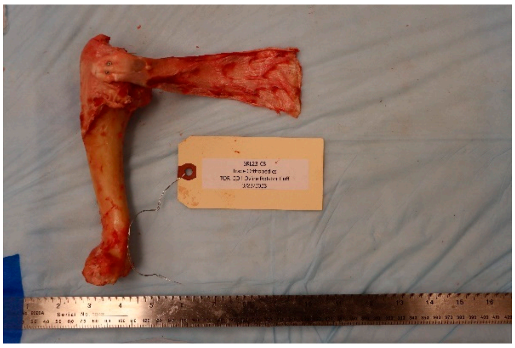</td></tr>
<tr><th>Ventral</th><th>Medial</th></tr>
<tr><td>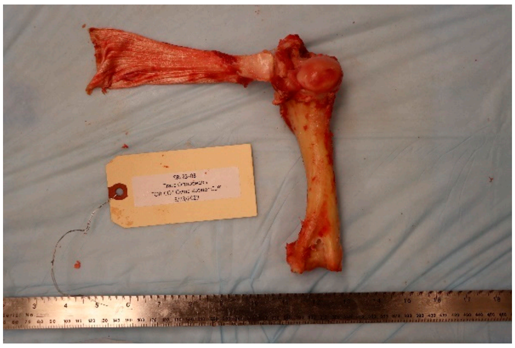</td><td>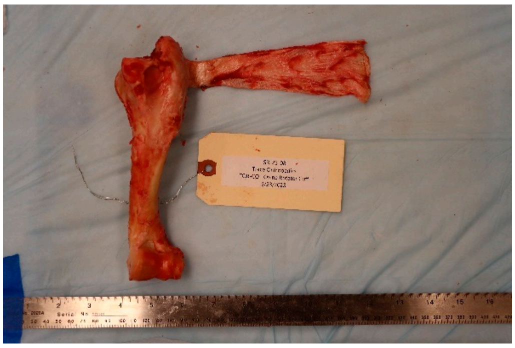</td></tr>
<tr><th colspan="2">Axial</th></tr>
<tr><td colspan="2">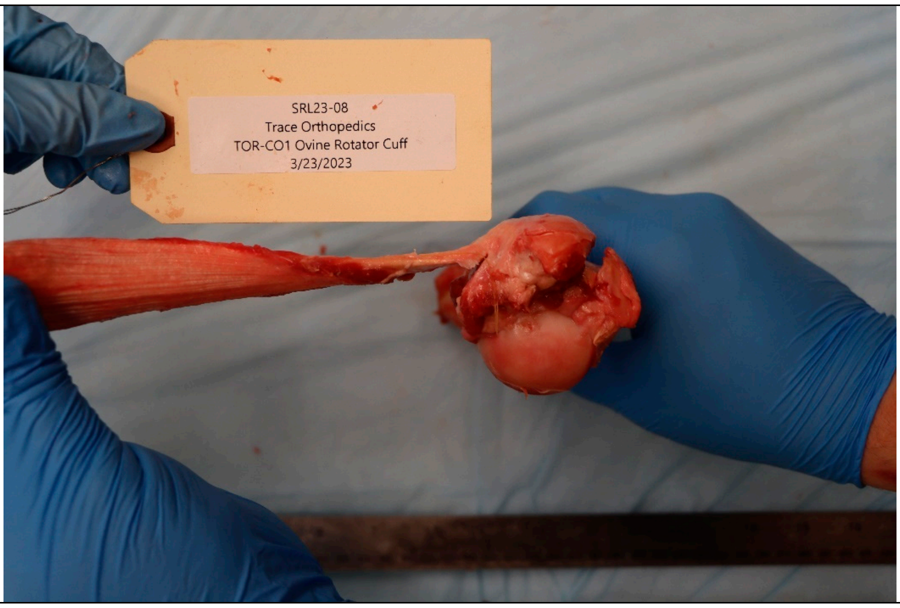
</td></tr>
</table>

<table>
<tr><th>Specimen Info</th><th>Dorsal</th><th>Lateral</th></tr>
<tr><td rowspan="5">Sample ID:
TOR-CO2

Group:
Treatment

Timepoint:
Baseline</td><td>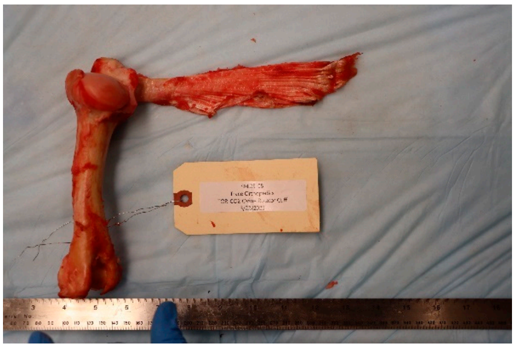</td><td>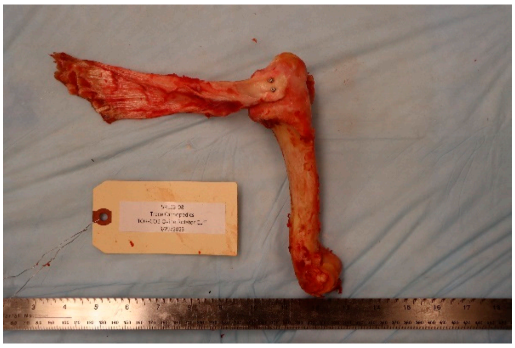</td></tr>
<tr><th>Ventral</th><th>Medial</th></tr>
<tr><td>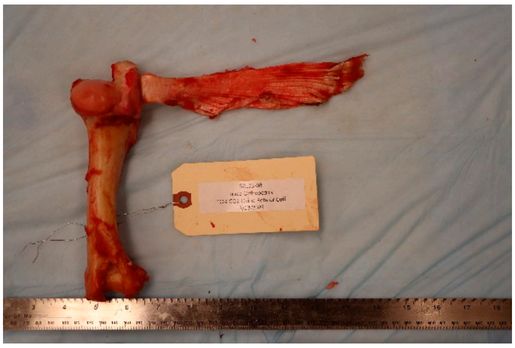</td><td>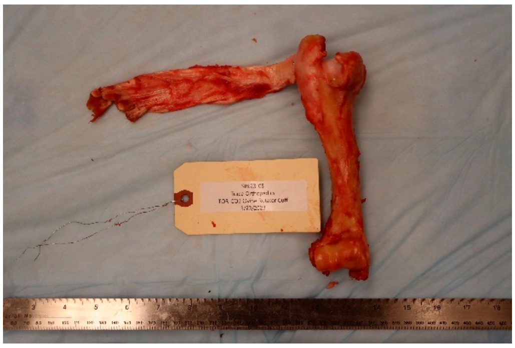</td></tr>
<tr><th colspan="2">Axial</th></tr>
<tr><td colspan="2">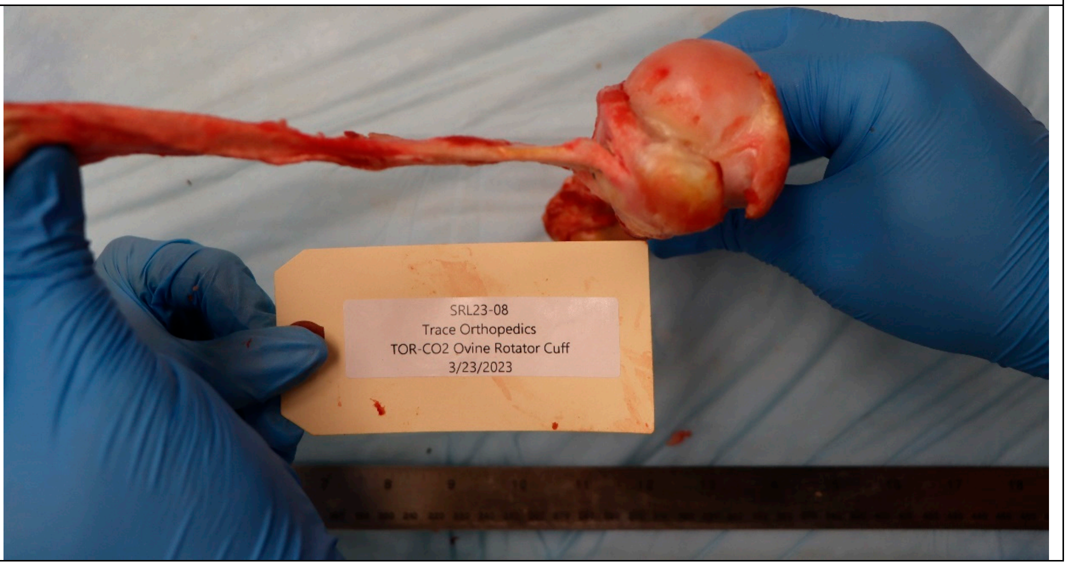
</td></tr>
</table>

<table>
<tr><th>Specimen Info</th><th>Dorsal</th><th>Lateral</th></tr>
<tr><td rowspan="5">Sample ID:
TOR-CO3

Group:
Treatment

Timepoint:
Baseline</td><td>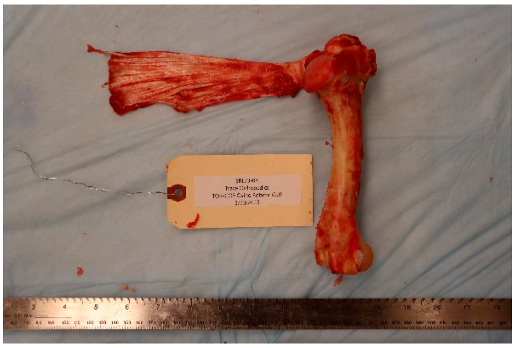</td><td>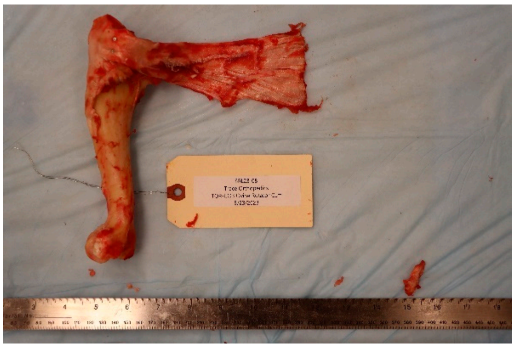</td></tr>
<tr><th>Ventral</th><th>Medial</th></tr>
<tr><td>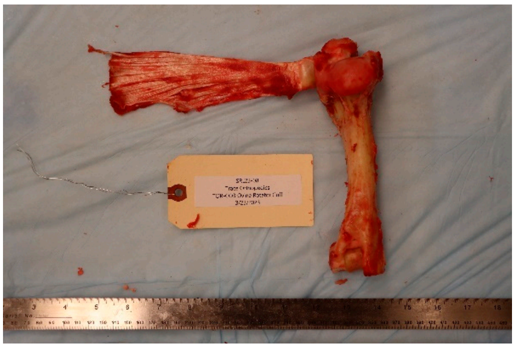</td><td>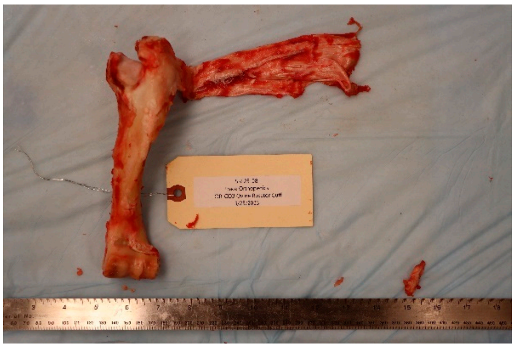</td></tr>
<tr><th colspan="2">Axial</th></tr>
<tr><td colspan="2">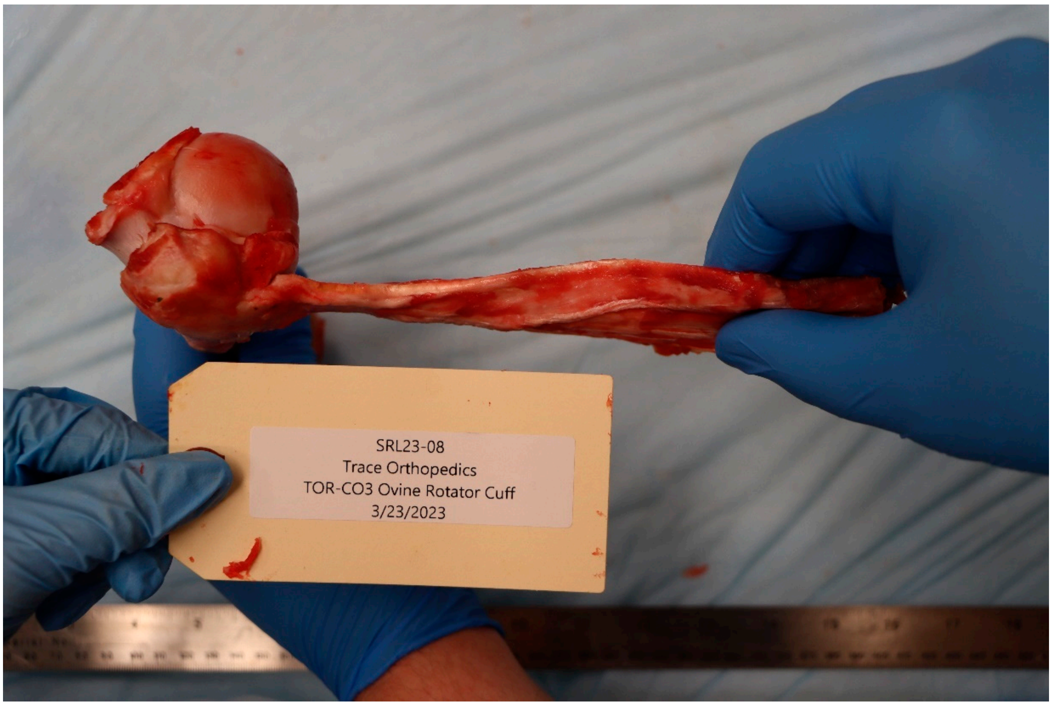
</td></tr>
</table>

<table>
<tr><th>Specimen Info</th><th>Dorsal</th><th>Lateral</th></tr>
<tr><td rowspan="5">Sample ID:
TOR-CO4

Group:
Treatment

Timepoint:
Baseline</td><td>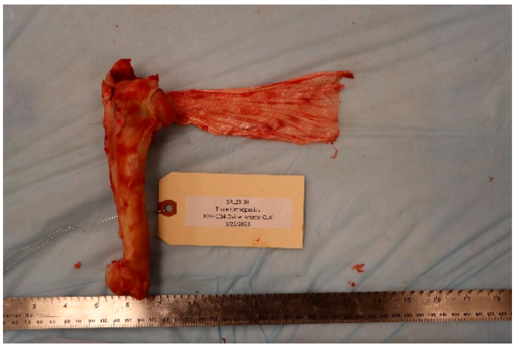</td><td>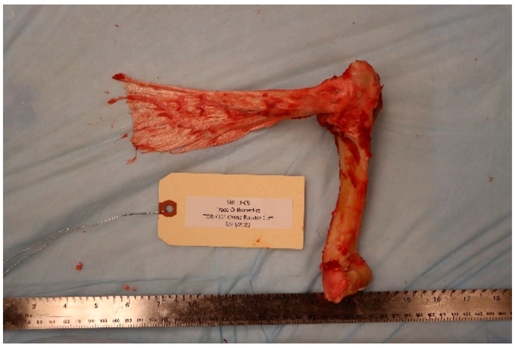</td></tr>
<tr><th>Ventral</th><th>Medial</th></tr>
<tr><td>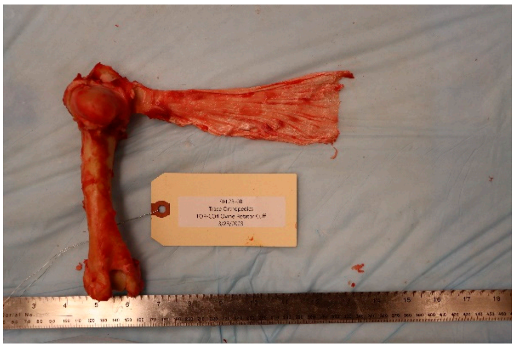</td><td>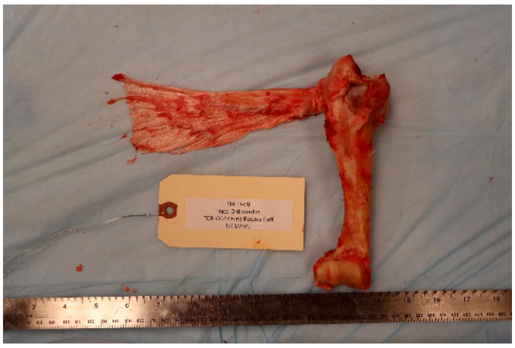</td></tr>
<tr><th colspan="2">Axial</th></tr>
<tr><td colspan="2">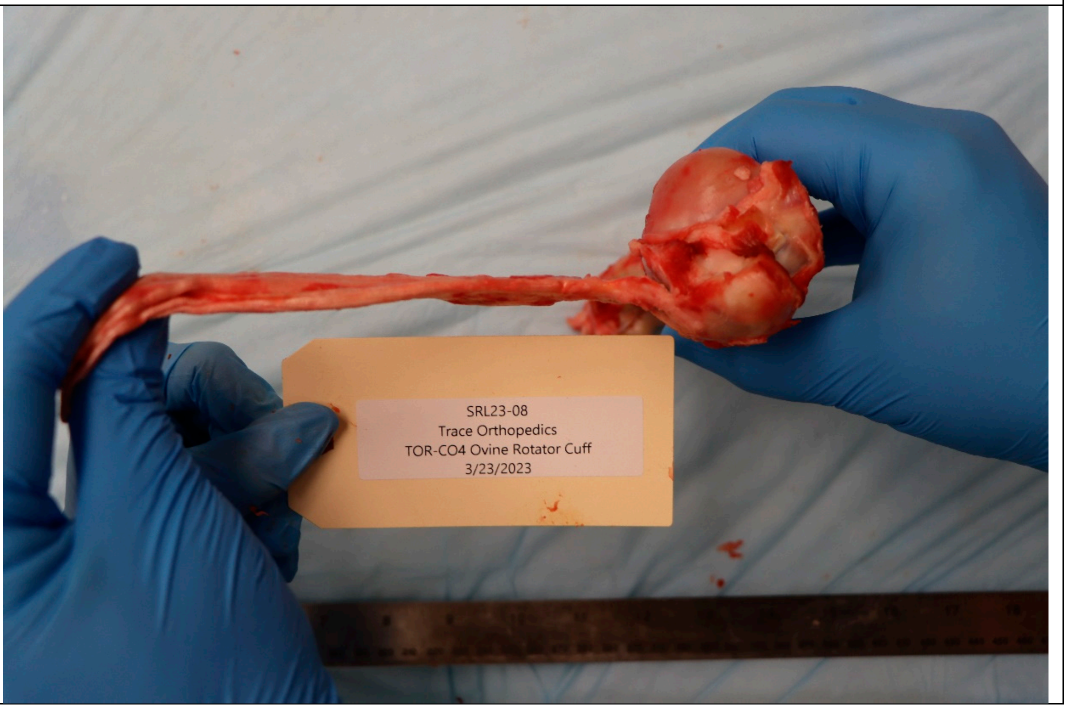
</td></tr>
</table>

<table>
<tr><th>Specimen Info</th><th>Dorsal</th><th>Lateral</th></tr>
<tr><td rowspan="5">**Sample ID:**
TOR-CO5

Group:
Treatment

Timepoint:
Baseline</td><td>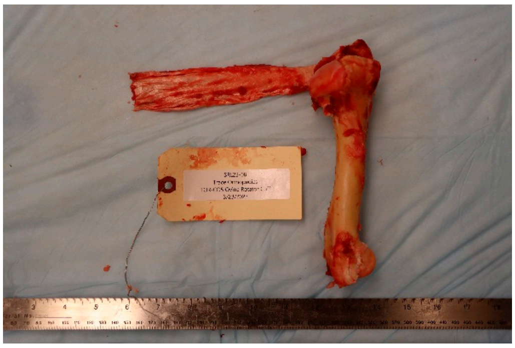</td><td>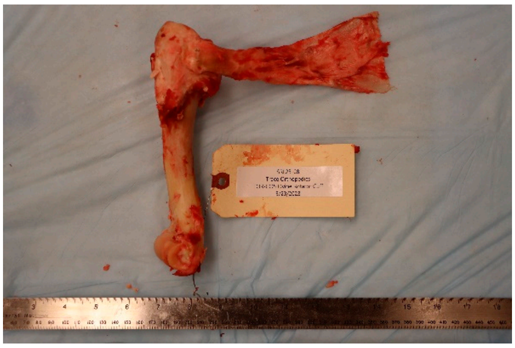</td></tr>
<tr><th>Ventral</th><th>Medial</th></tr>
<tr><td>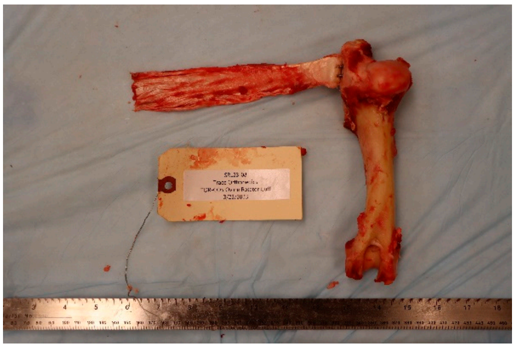</td><td>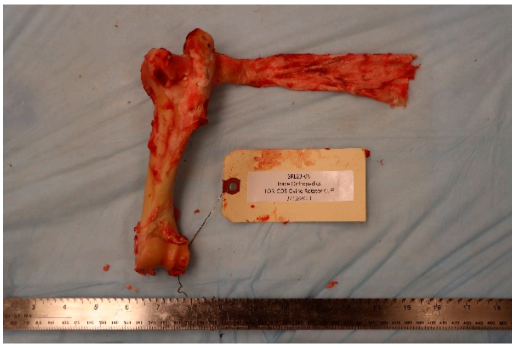</td></tr>
<tr><th colspan="2">Axial</th></tr>
<tr><td colspan="2">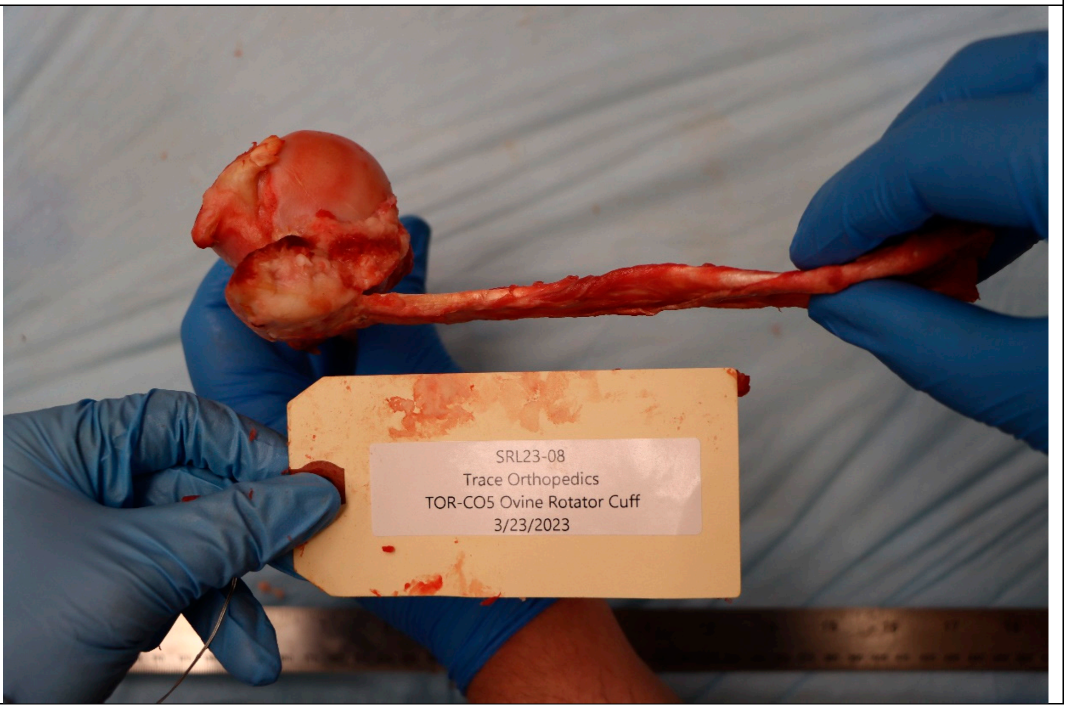
</td></tr>
</table>

<table>
<tr><th>Specimen Info</th><th>Dorsal</th><th>Lateral</th></tr>
<tr><td rowspan="5">Sample ID:
TOR-CO6

Group:
Treatment

Timepoint:
Baseline</td><td>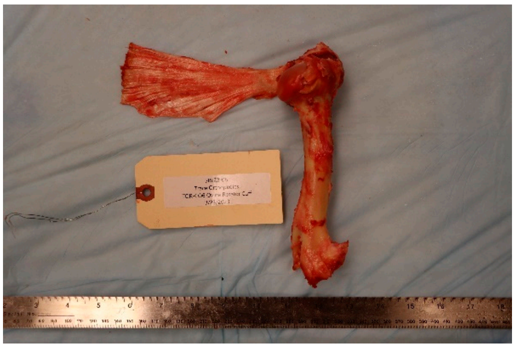</td><td>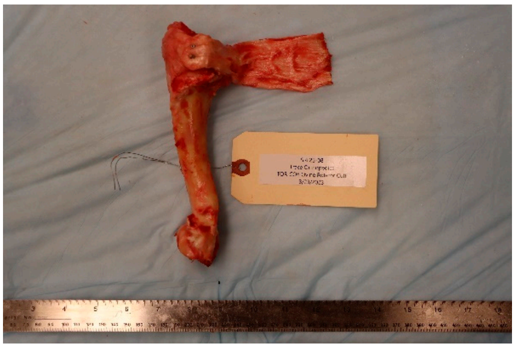</td></tr>
<tr><th>Ventral</th><th>Medial</th></tr>
<tr><td>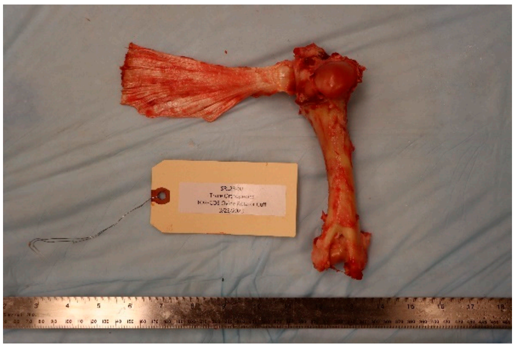</td><td>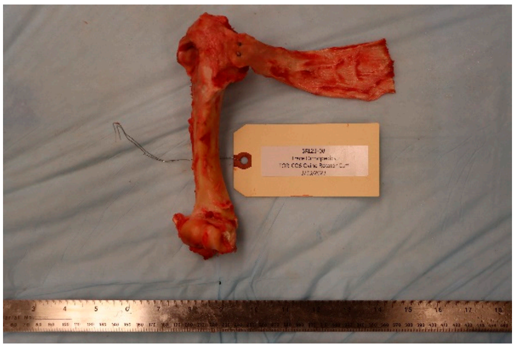</td></tr>
<tr><th colspan="2">Axial</th></tr>
<tr><td colspan="2">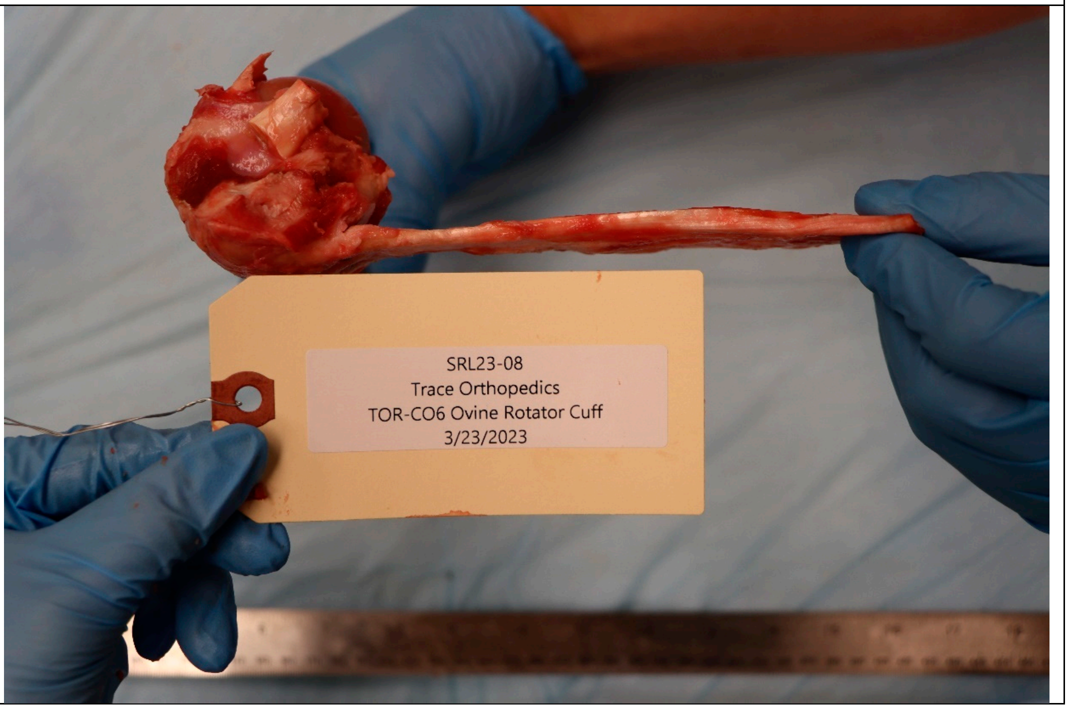
</td></tr>
</table>

<table>
<tr><th>Specimen Info</th><th>Dorsal</th><th>Lateral</th></tr>
<tr><td rowspan="5">Sample ID:
TOR-CO7

Group:
Treatment

Timepoint:
Baseline</td><td>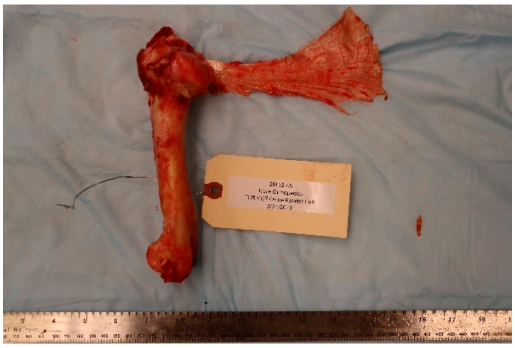</td><td>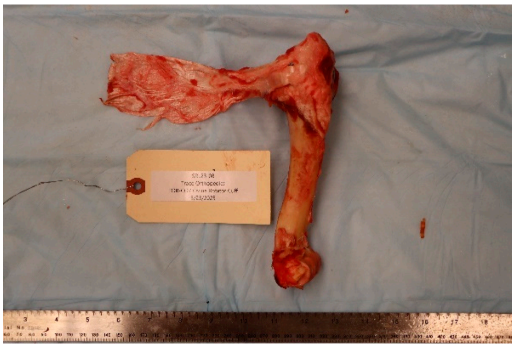</td></tr>
<tr><th>Ventral</th><th>Medial</th></tr>
<tr><td>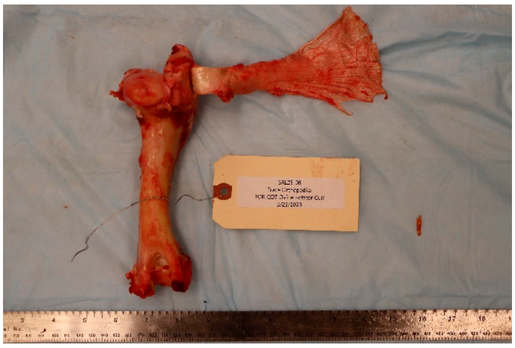</td><td>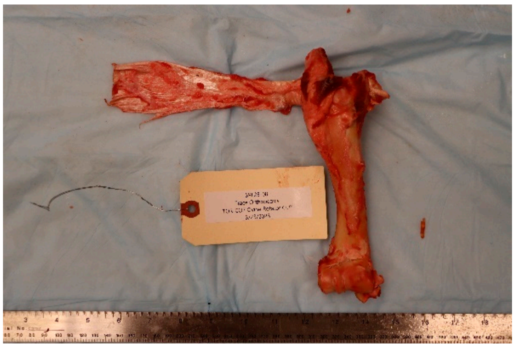</td></tr>
<tr><th colspan="2">Axial</th></tr>
<tr><td colspan="2">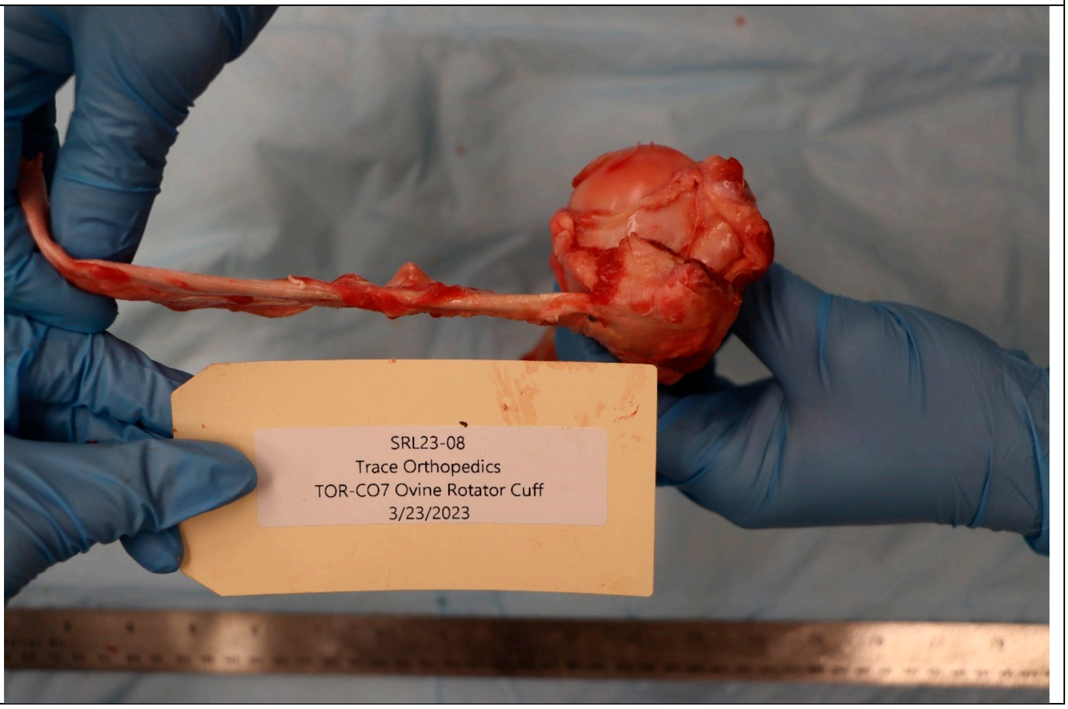
</td></tr>
</table>

<table>
<tr><th>Specimen Info</th><th>Dorsal</th><th>Lateral</th></tr>
<tr><td rowspan="5">**Sample ID:**
TOR-CO8

Group:
Treatment

Timepoint:
Baseline</td><td>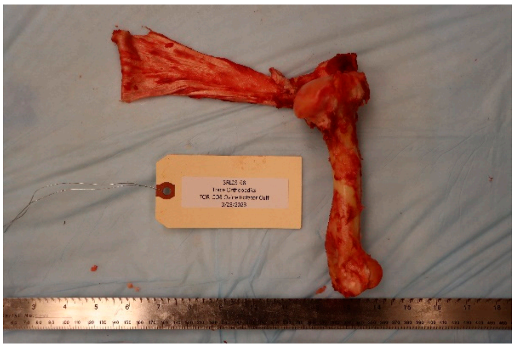</td><td>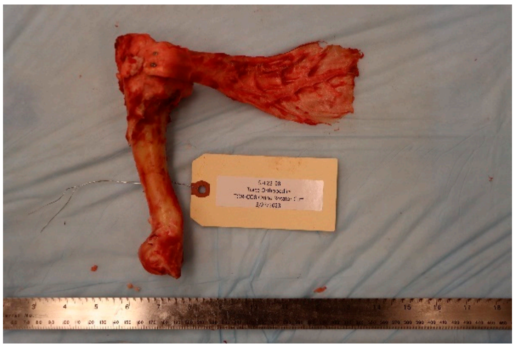</td></tr>
<tr><th>Ventral</th><th>Medial</th></tr>
<tr><td>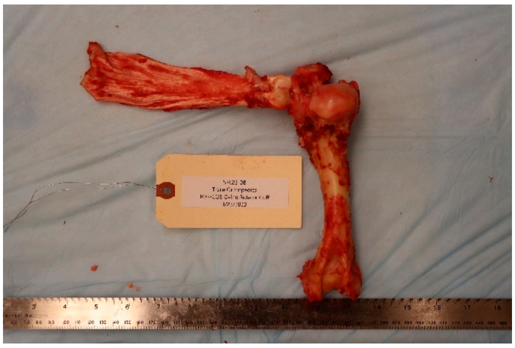</td><td>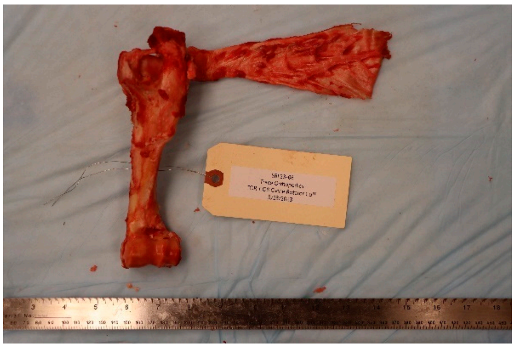</td></tr>
<tr><th colspan="2">Axial</th></tr>
<tr><td colspan="2">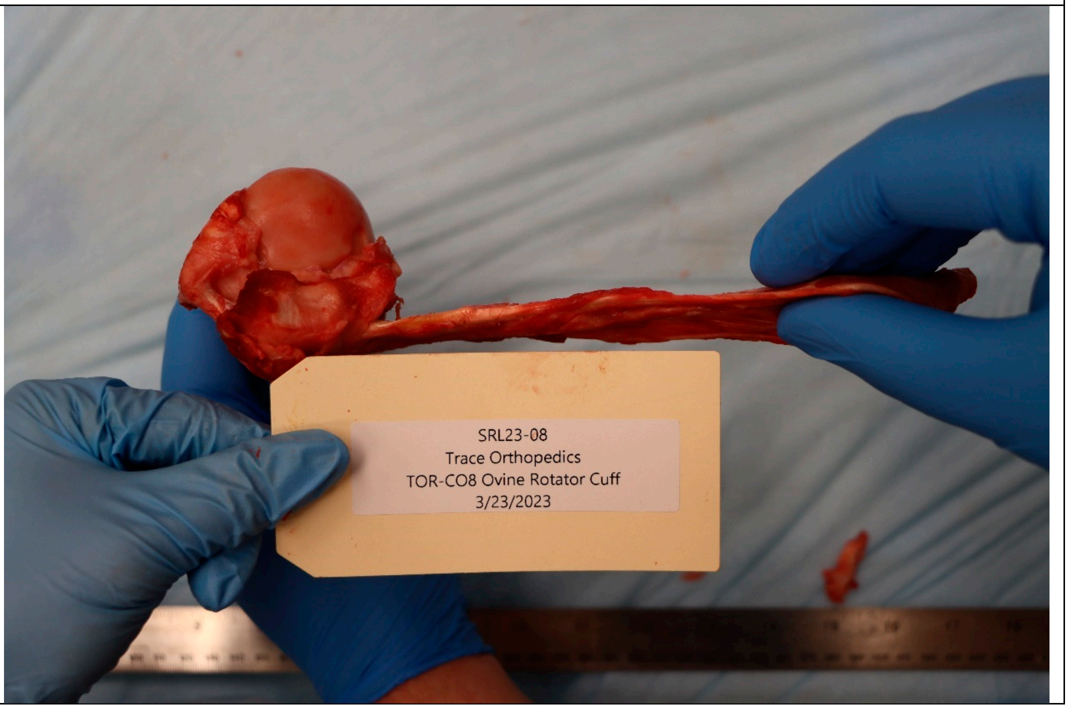
</td></tr>
</table>

<table>
<tr><th>Specimen Info</th><th>Dorsal</th><th>Lateral</th></tr>
<tr><td rowspan="5">Sample ID:
TOR-CO9

Group:
Treatment

Timepoint:
Baseline</td><td>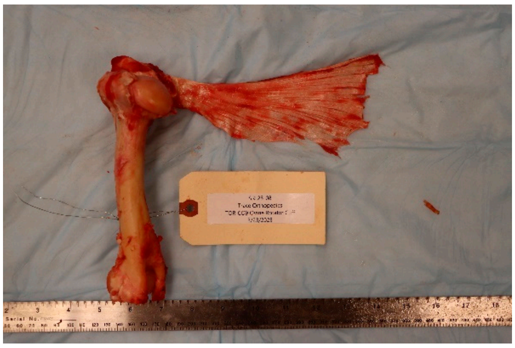</td><td>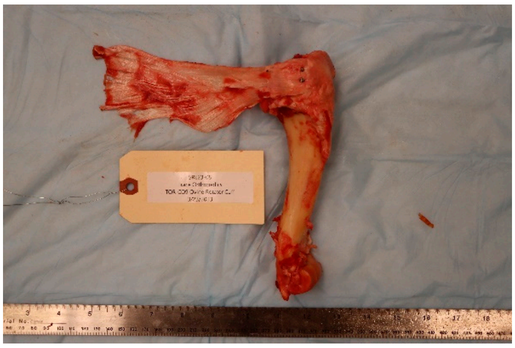</td></tr>
<tr><th>Ventral</th><th>Medial</th></tr>
<tr><td>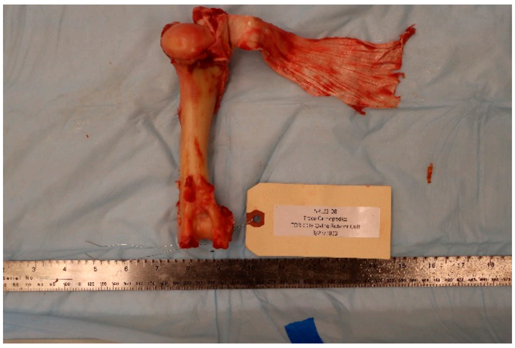</td><td>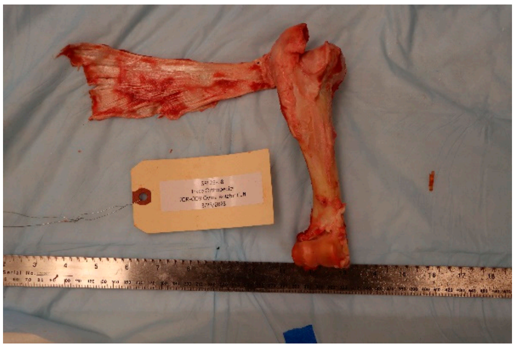</td></tr>
<tr><th colspan="2">Axial</th></tr>
<tr><td colspan="2">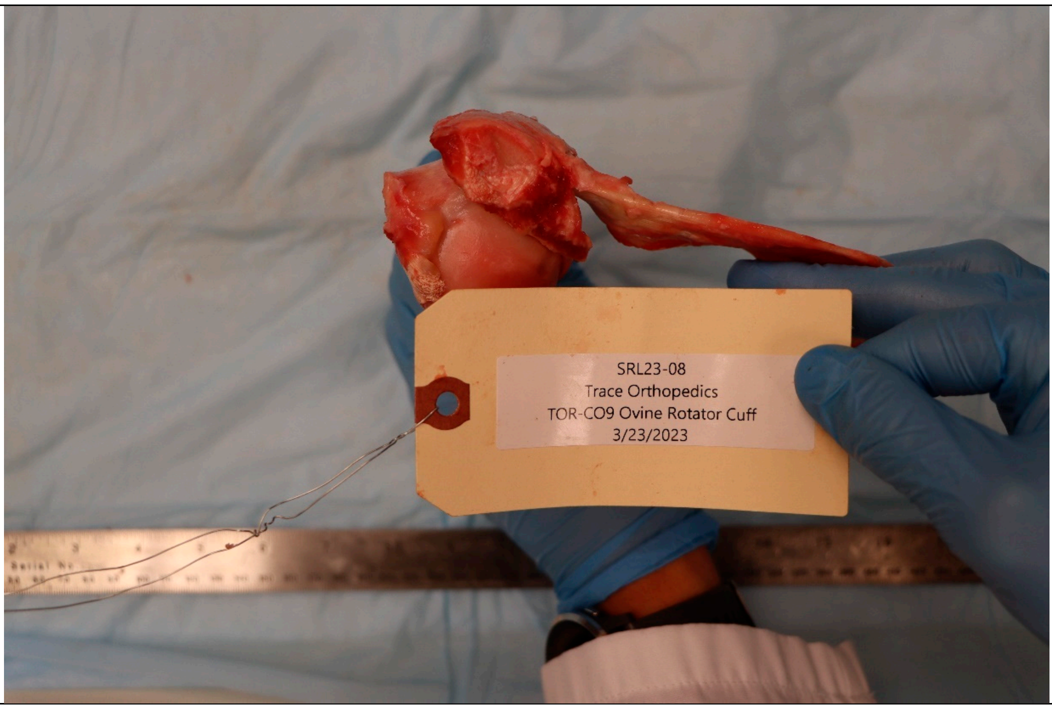
</td></tr>
</table>

<table>
<tr><th>Specimen Info</th><th>Dorsal</th><th>Lateral</th></tr>
<tr><td rowspan="5">Sample ID:
TOR-C10

Group:
Control

Timepoint:
Baseline</td><td>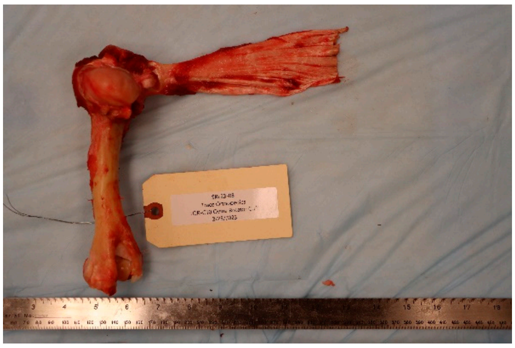</td><td>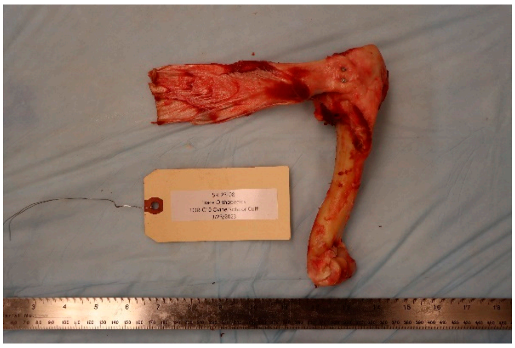</td></tr>
<tr><th>Ventral</th><th>Medial</th></tr>
<tr><td>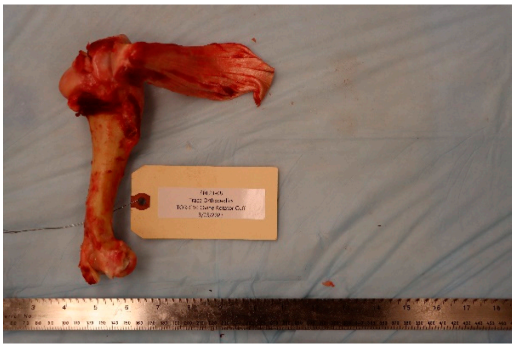</td><td>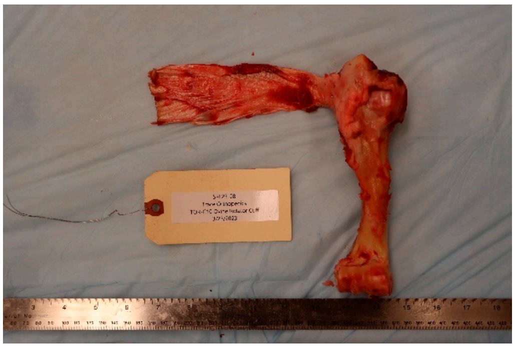</td></tr>
<tr><th colspan="2">Axial</th></tr>
<tr><td colspan="2">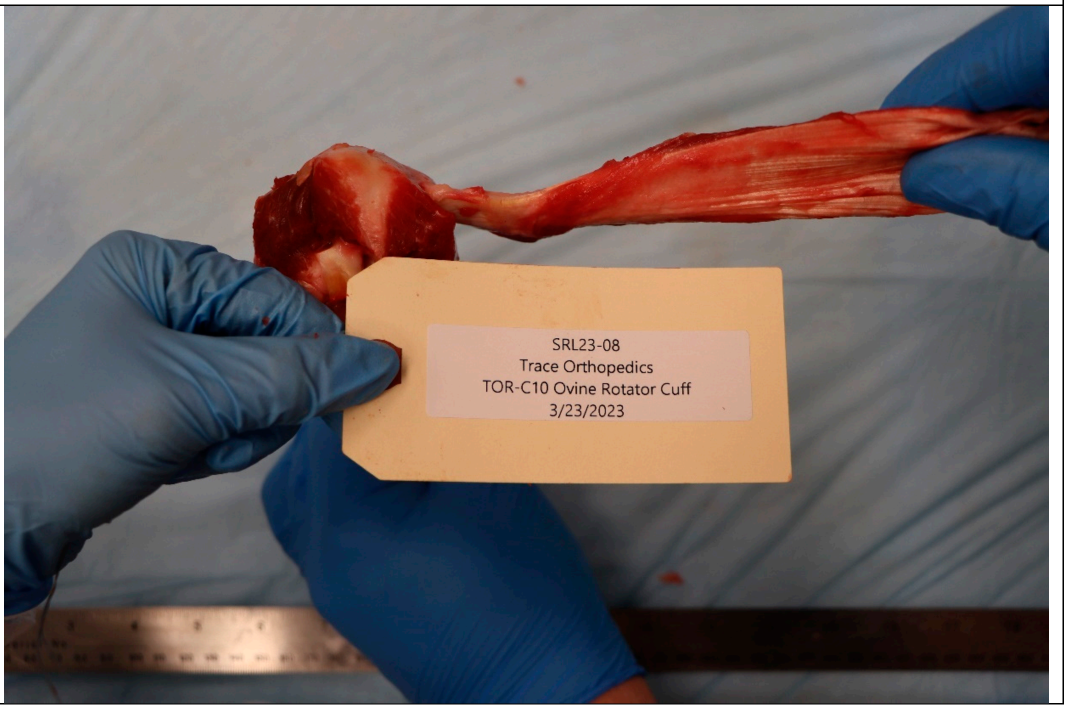
</td></tr>
</table>

<table>
<tr><th>Specimen Info</th><th>Dorsal</th><th>Lateral</th></tr>
<tr><td rowspan="5">Sample ID:
TOR-C11

Group:
Control

Timepoint:
Baseline</td><td>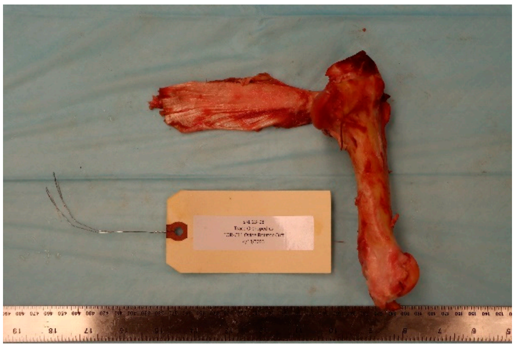</td><td>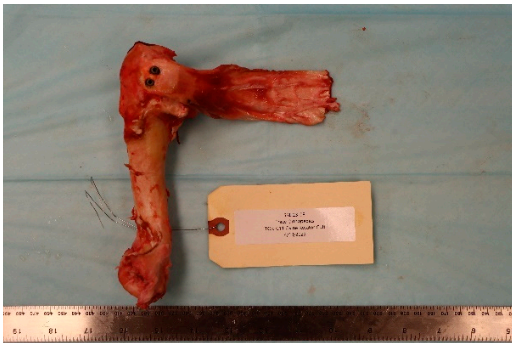</td></tr>
<tr><th>Ventral</th><th>Medial</th></tr>
<tr><td>No Image</td><td>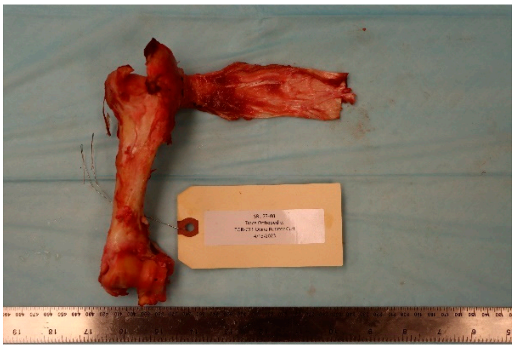</td></tr>
<tr><th colspan="2">Axial</th></tr>
<tr><td colspan="2">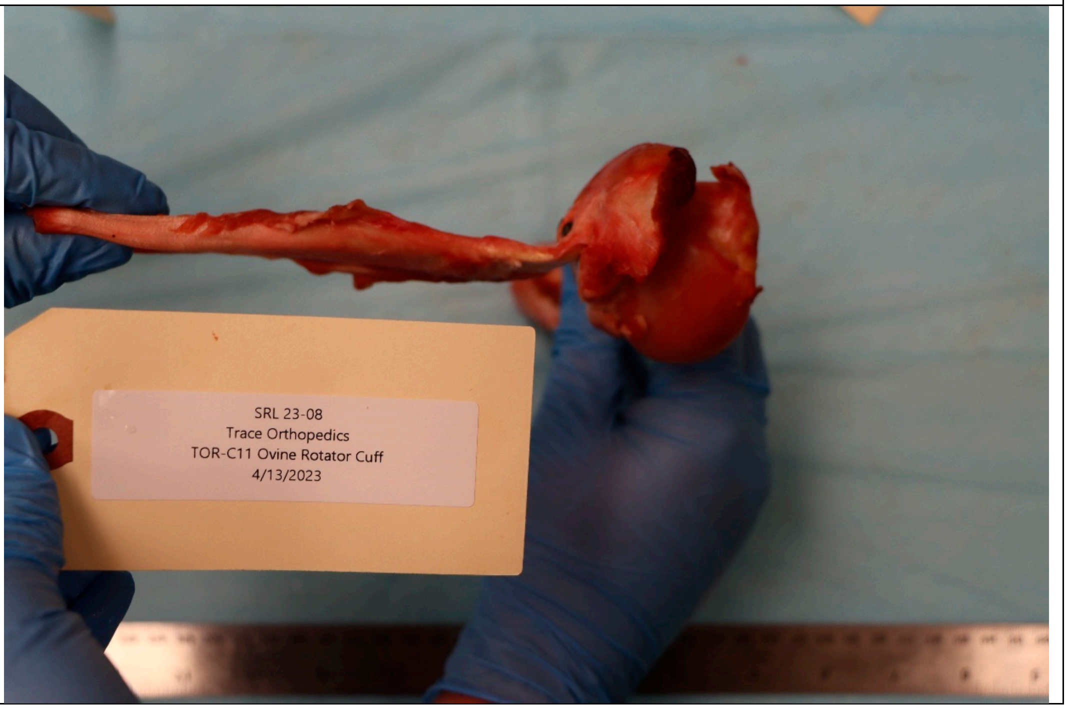
</td></tr>
</table>

<table>
<tr><th>Specimen Info</th><th colspan="2">Dorsal</th><th colspan="2">Lateral</th></tr>
<tr><td rowspan="5">**Sample ID:**
TOR-C12

Group:
Control

Timepoint:
Baseline</td><td colspan="2">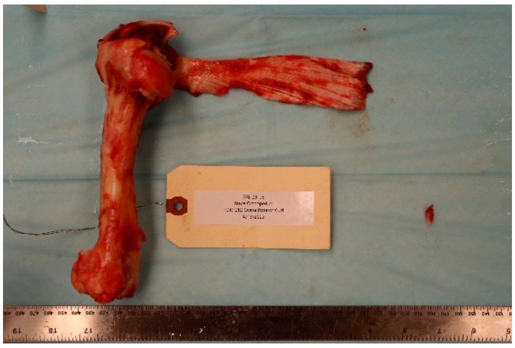</td><td colspan="2">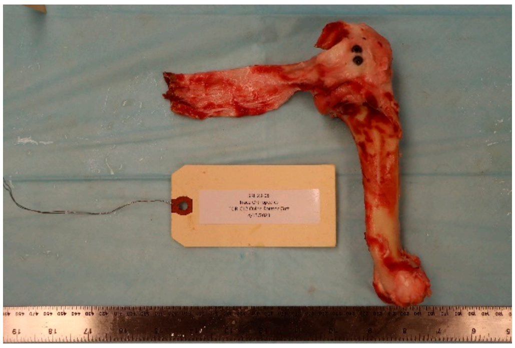</td></tr>
<tr><th colspan="2">Ventral</th><th colspan="2">Medial</th></tr>
<tr><td colspan="2">No Image</td><td colspan="2">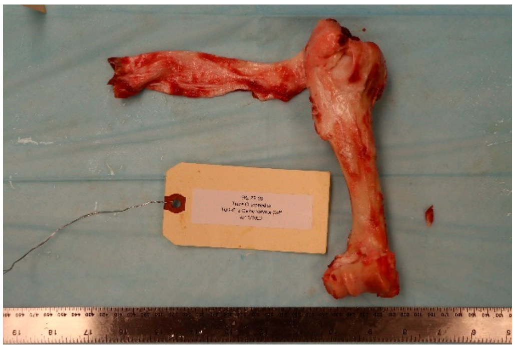</td></tr>
<tr><th colspan="4">Axial</th></tr>
<tr><td colspan="4">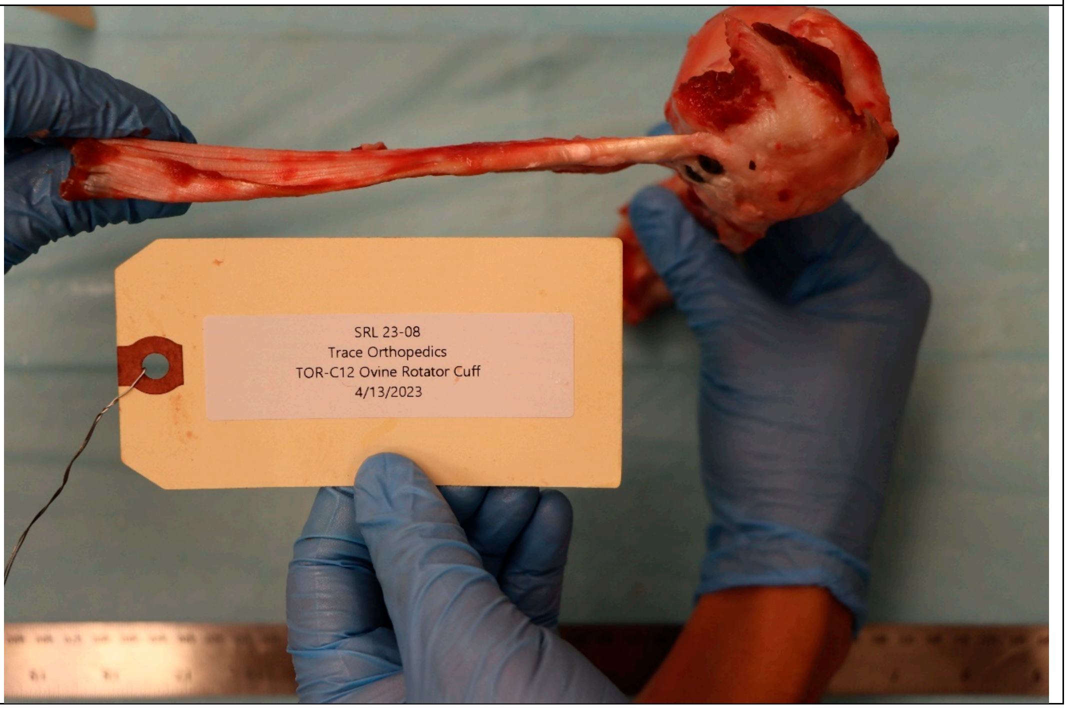
</td></tr>
</table>

<table>
<tr><th>Specimen Info</th><th>Dorsal</th><th>Lateral</th></tr>
<tr><td rowspan="5">Sample ID:
TOR-C13

Group:
Control

Timepoint:
Baseline</td><td>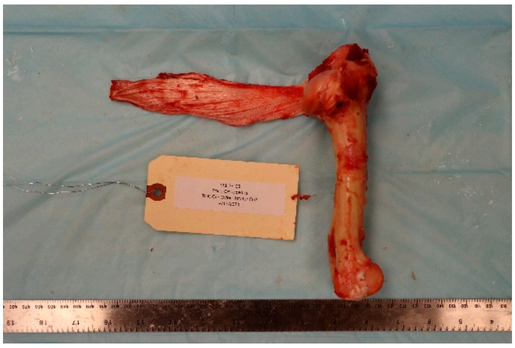</td><td>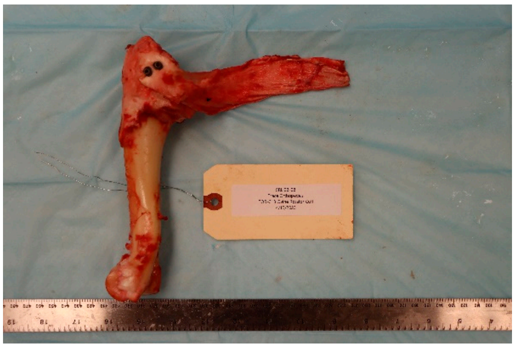</td></tr>
<tr><th>Ventral</th><th>Medial</th></tr>
<tr><td>No Image</td><td>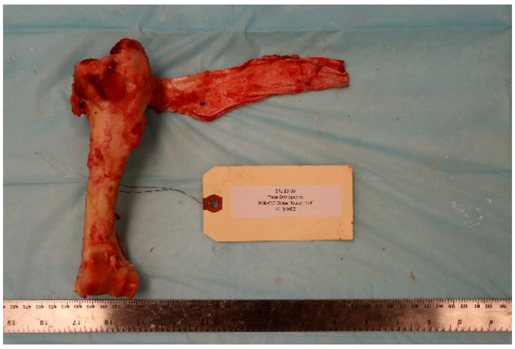</td></tr>
<tr><th colspan="2">Axial</th></tr>
<tr><td colspan="2">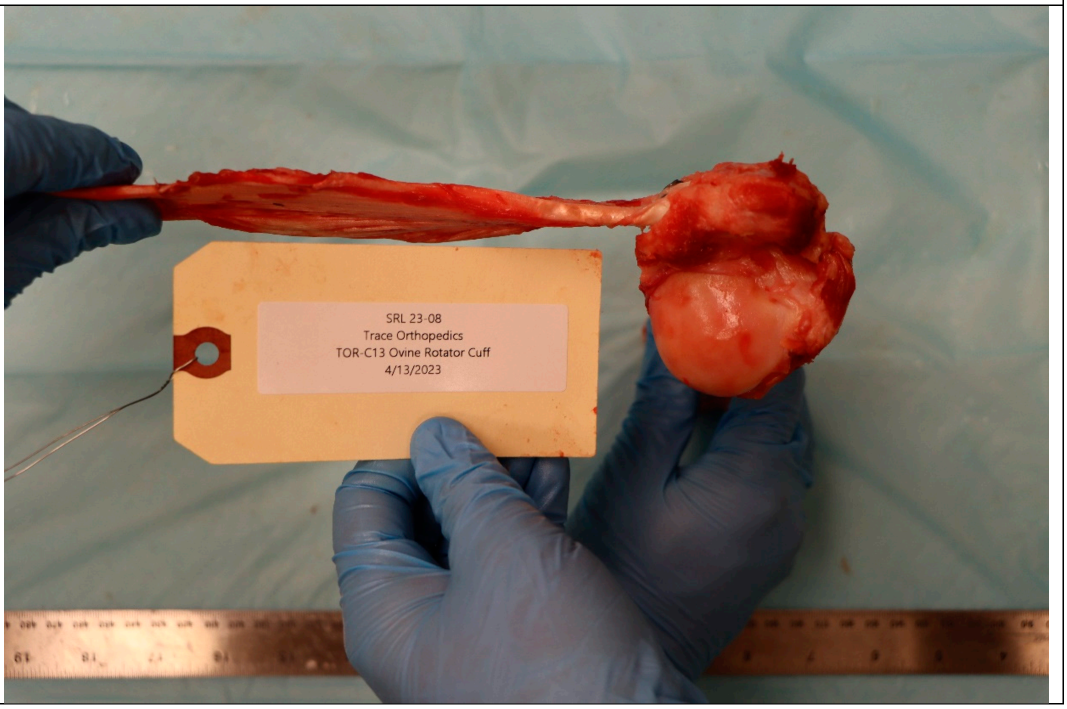
</td></tr>
</table>

<table>
<tr><th>Specimen Info</th><th>Dorsal</th><th>Lateral</th></tr>
<tr><td rowspan="5">Sample ID:
TOR-C14

Group:
Control

Timepoint:
Baseline</td><td>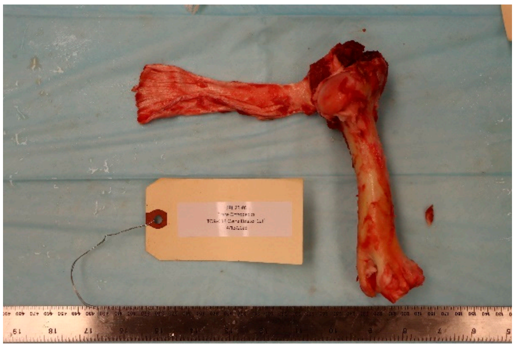</td><td>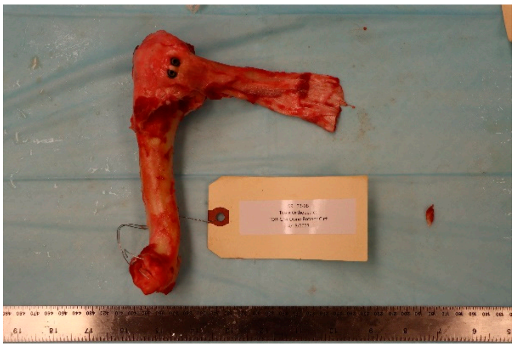</td></tr>
<tr><th>Ventral</th><th>Medial</th></tr>
<tr><td>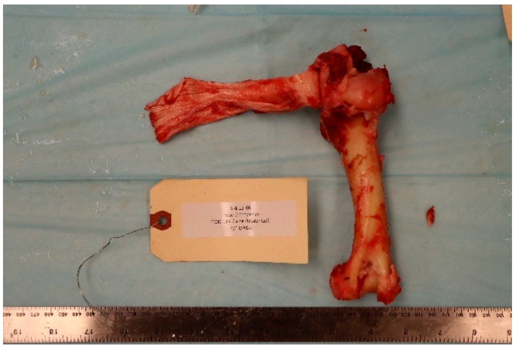</td><td>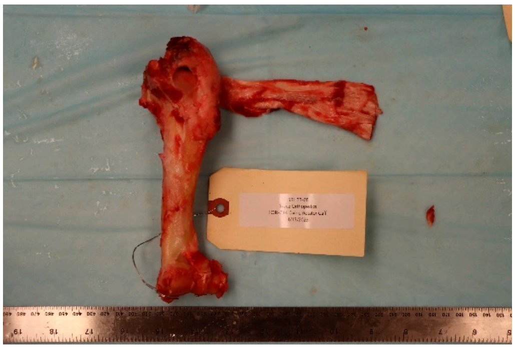</td></tr>
<tr><th colspan="2">Axial</th></tr>
<tr><td colspan="2">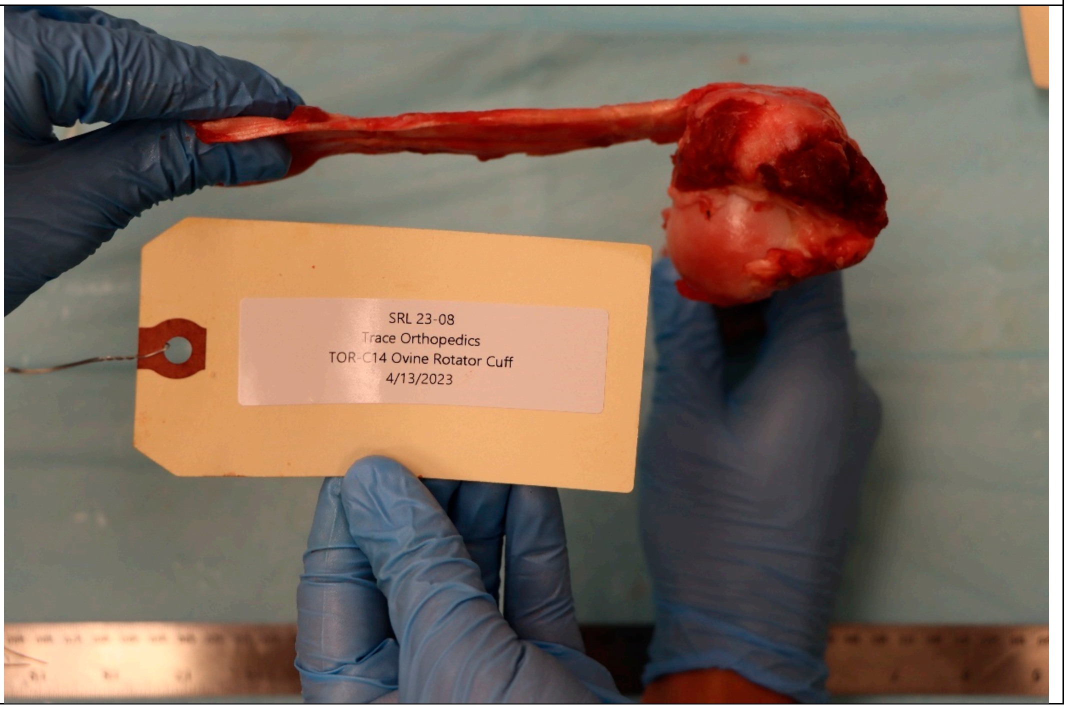
</td></tr>
</table>

Specimen Info	Dorsal	Lateral
	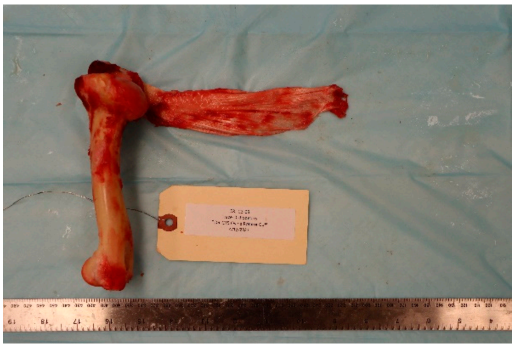	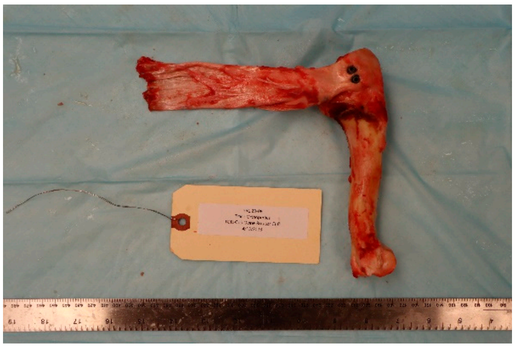
	**Ventral**	**Medial**
	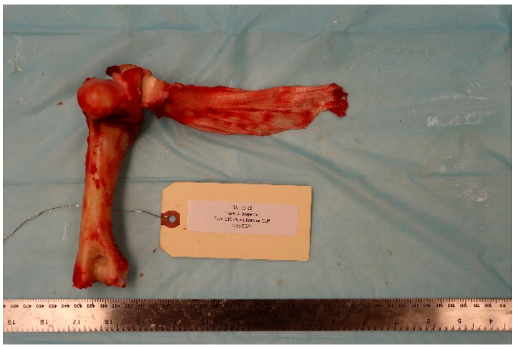	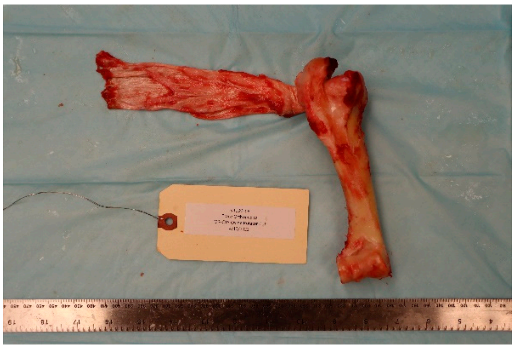
**Sample ID:**<br>TOR-C15<br><br>**Group:**<br>Control<br><br>**Timepoint:**<br>Baseline	**Axial**	
	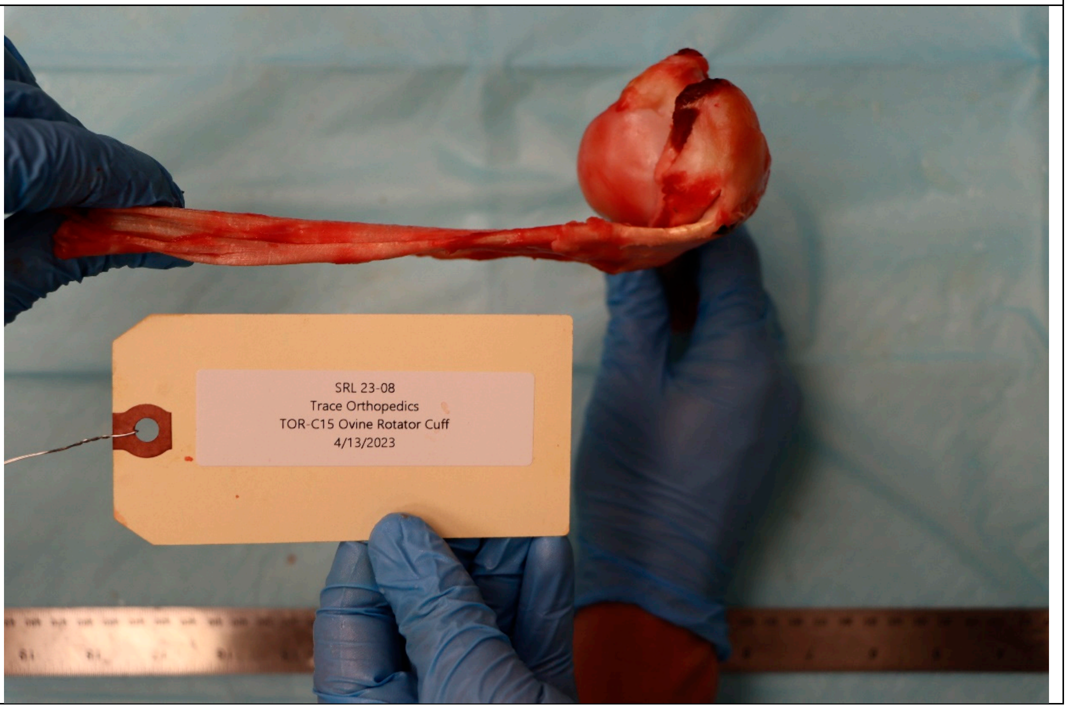	

<table>
<tr><th>Specimen Info</th><th>Dorsal</th><th>Lateral</th></tr>
<tr><td rowspan="5">Sample ID:
TOR-C16

Group:
Control

Timepoint:
Baseline</td><td>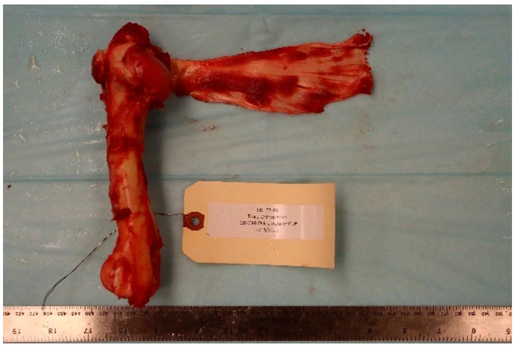</td><td>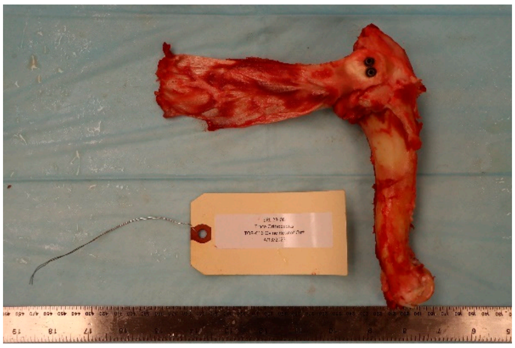</td></tr>
<tr><th>Ventral</th><th>Medial</th></tr>
<tr><td>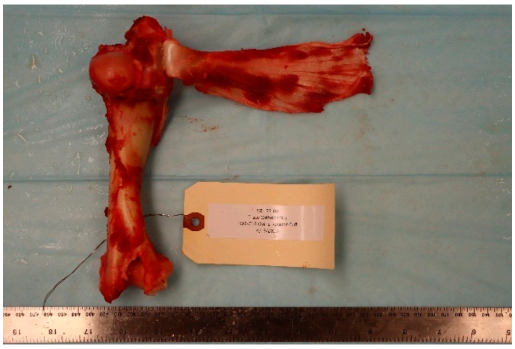</td><td>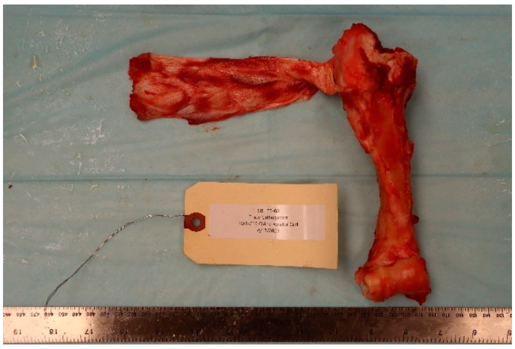</td></tr>
<tr><th colspan="2">Axial</th></tr>
<tr><td colspan="2">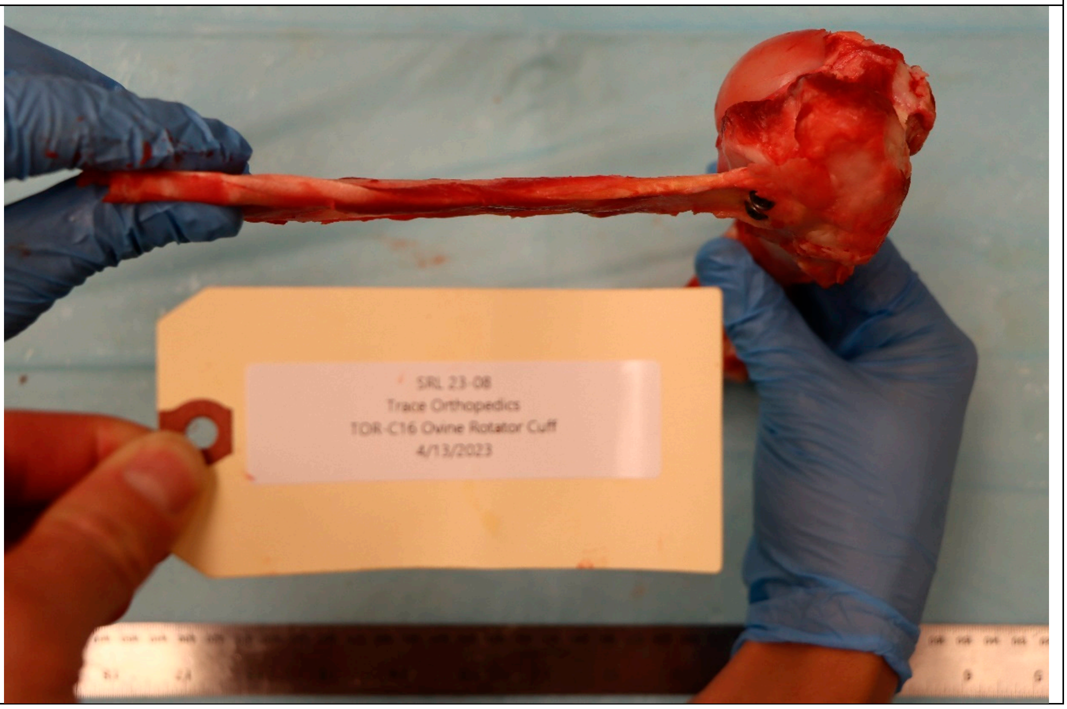
</td></tr>
</table>

Specimen Info	Dorsal	Lateral
	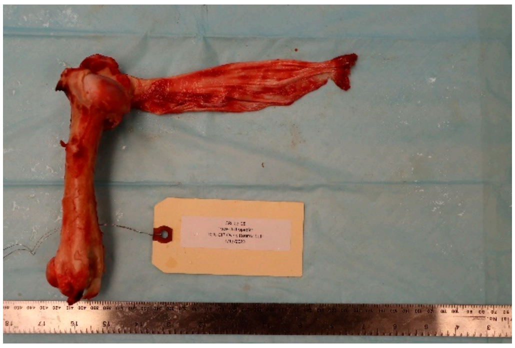	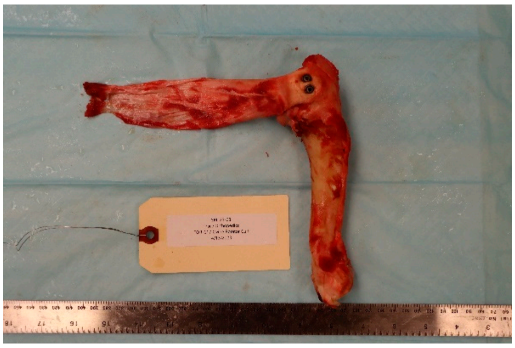
	**Ventral**	**Medial**
**Sample ID:**<br>TOR-C17<br><br>**Group:**<br>Control<br><br>**Timepoint:**<br>Baseline	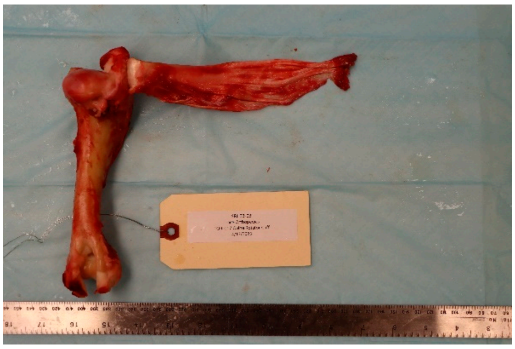	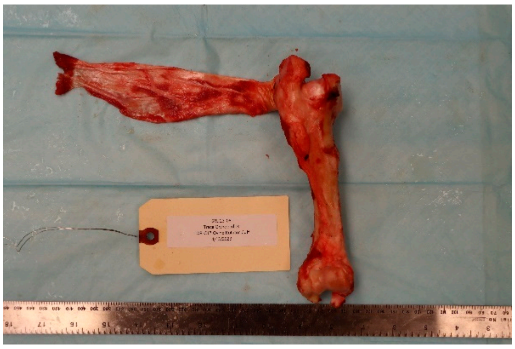
	**Axial**	
	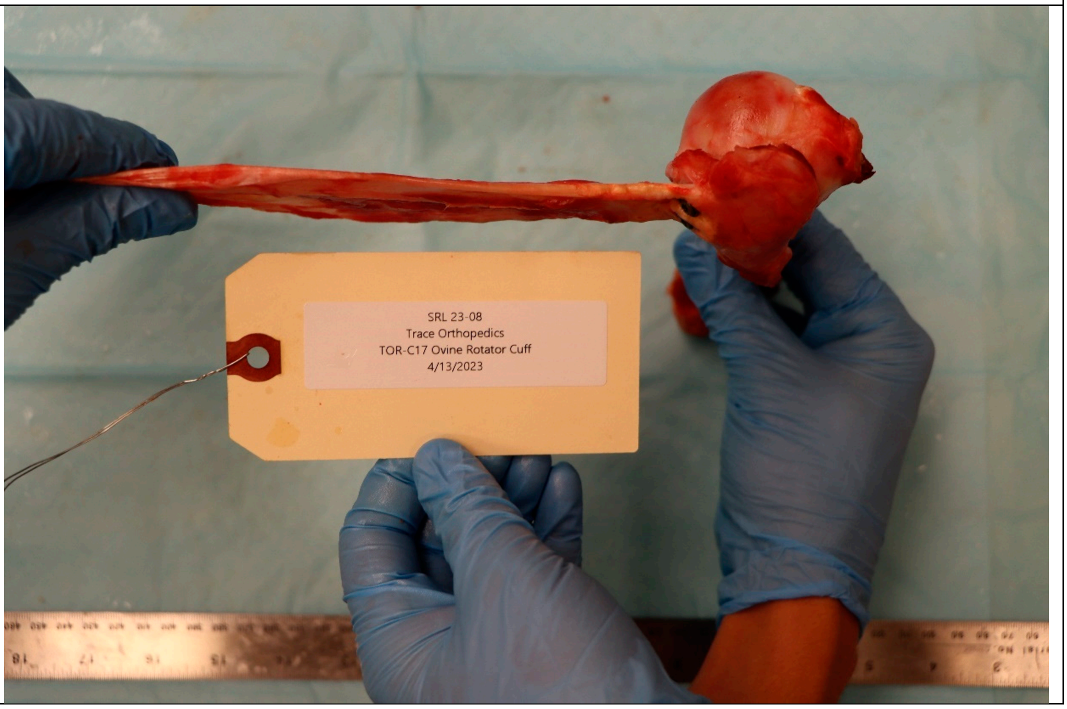	

Specimen Info	Dorsal	Lateral
	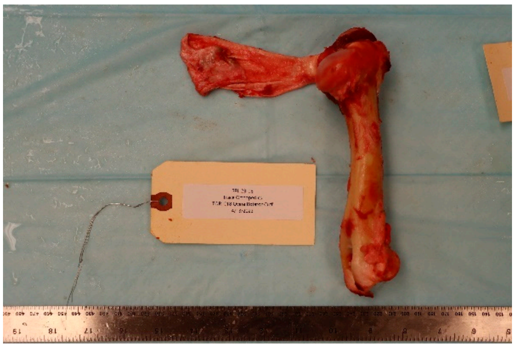	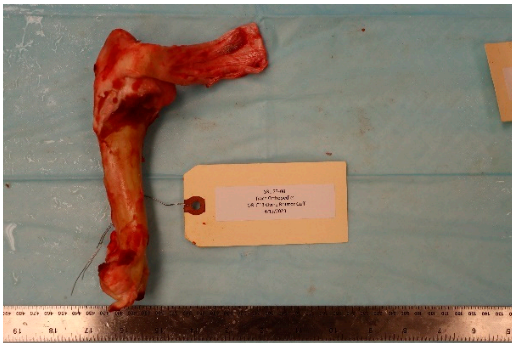
	**Ventral**	**Medial**
	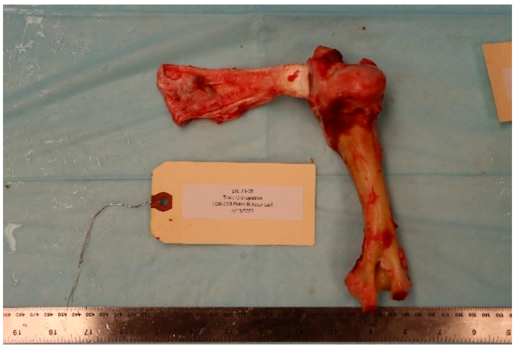	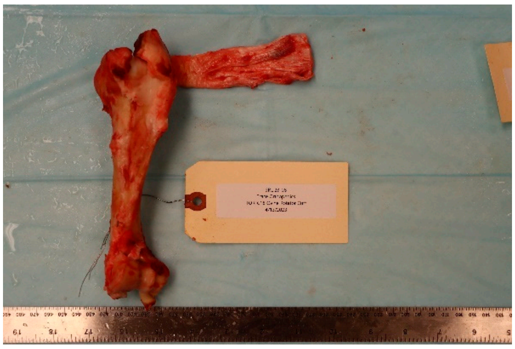
**Sample ID:**<br>TOR-C18<br><br>**Group:**<br>Control<br><br>**Timepoint:**<br>Baseline	**Axial**	

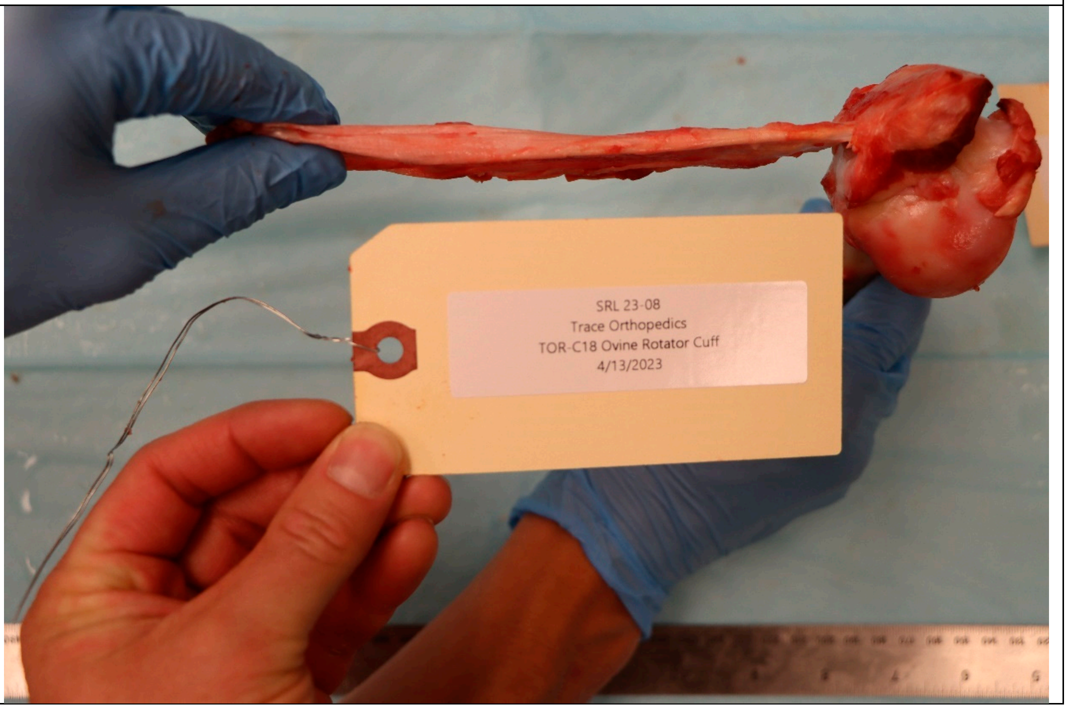

<table>
<tr><th>Specimen Info</th><th>Dorsal</th><th>Lateral</th></tr>
<tr><td rowspan="5">

Sample ID:
TOR-C19

Group:
Control

Timepoint:
Baseline

</td><td>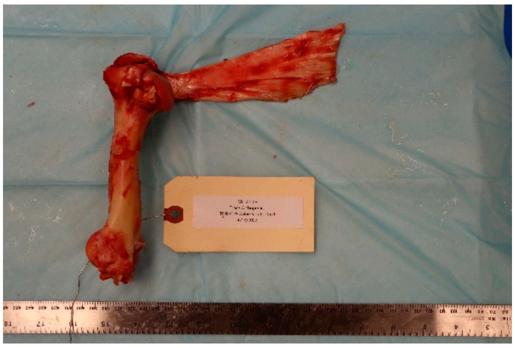</td><td>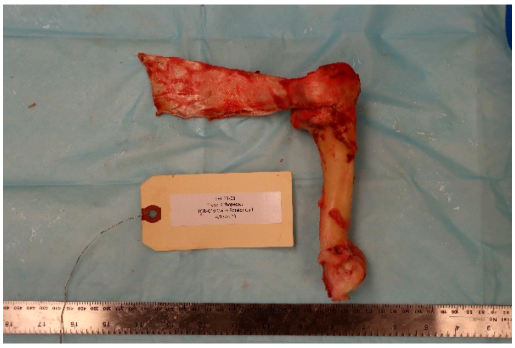</td></tr>
<tr><th>Ventral</th><th>Medial</th></tr>
<tr><td>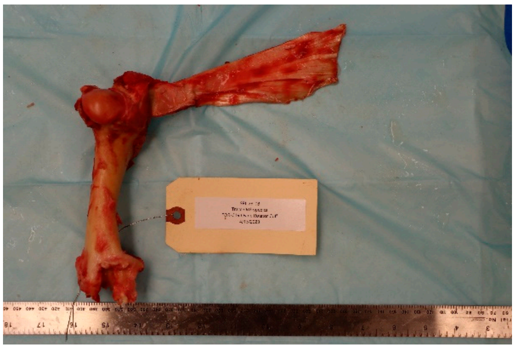</td><td>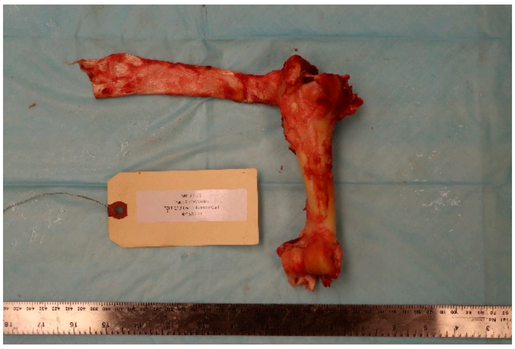</td></tr>
<tr><th colspan="2">Axial</th></tr>
<tr><td colspan="2">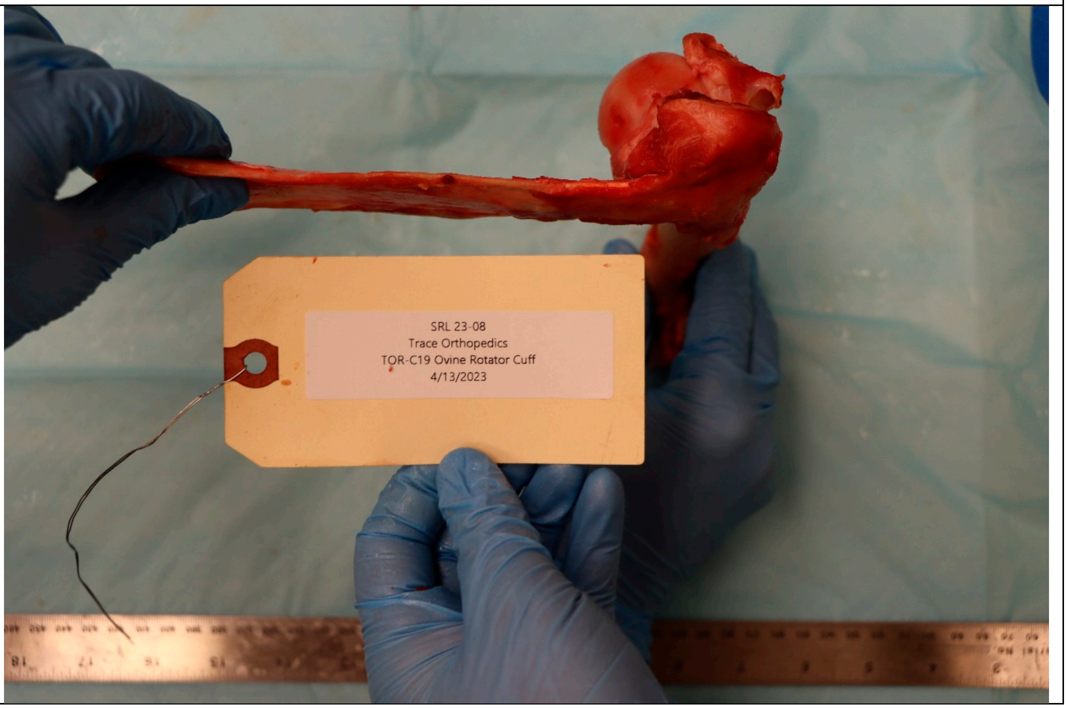
</td></tr>
</table>

<table>
<tr><th>Specimen Info</th><th>Dorsal</th><th>Lateral</th></tr>
<tr><td rowspan="5">Sample ID:
TOR-C20

Group:
Control

Timepoint:
Baseline</td><td>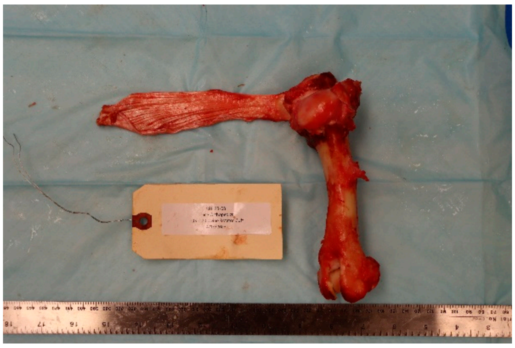</td><td>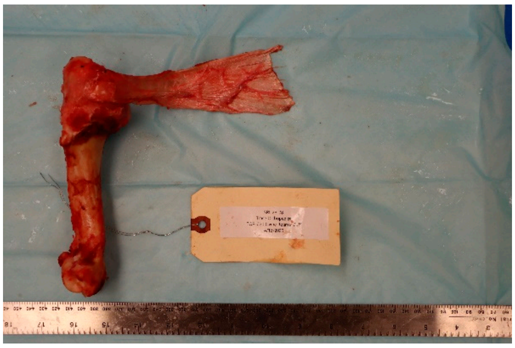</td></tr>
<tr><th>Ventral</th><th>Medial</th></tr>
<tr><td>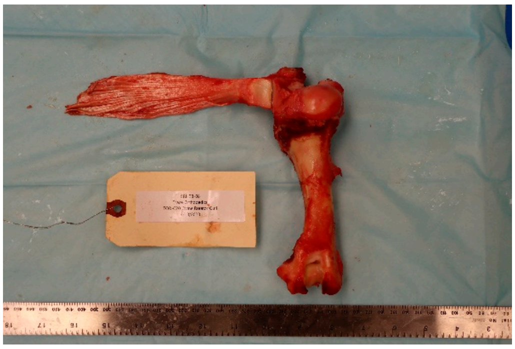</td><td>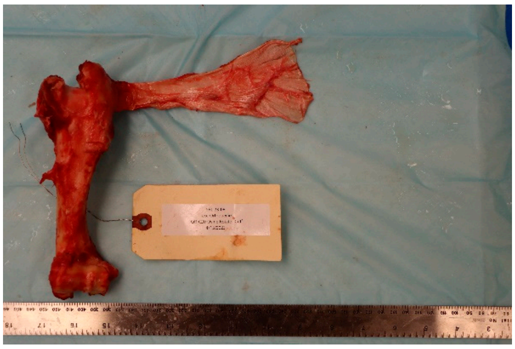</td></tr>
<tr><th colspan="2">Axial</th></tr>
<tr><td colspan="2">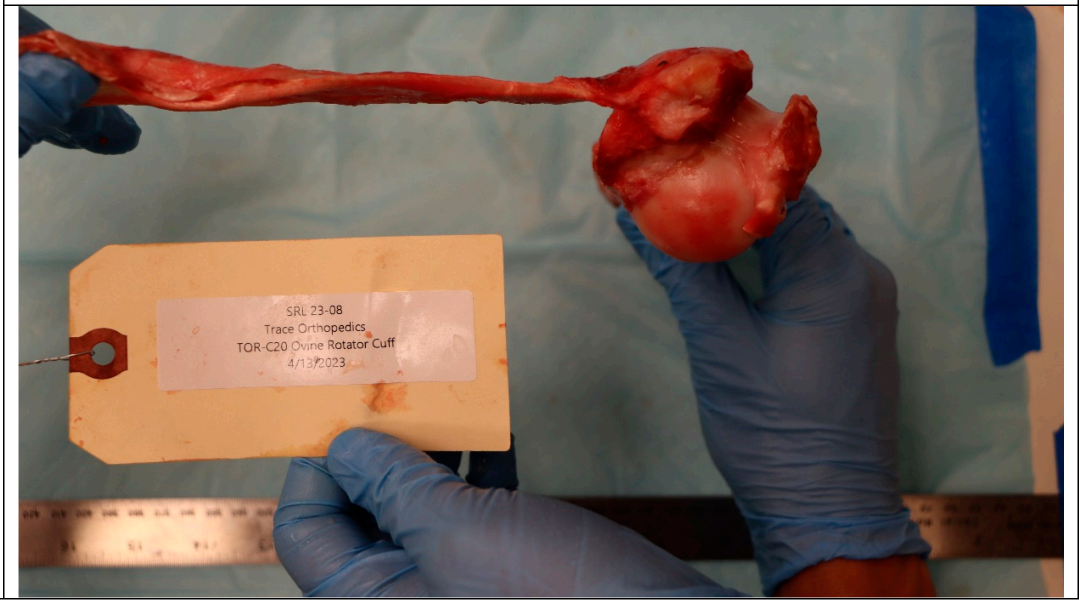
</td></tr>
</table>

Supplementary File B

<table>
<tr><th>Specimen Info</th><th colspan="2">Post-Testing</th></tr>
<tr><td rowspan="3">Sample ID:
TOR-CO1

Group:
Treatment

Timepoint:
Baseline</td><td colspan="2">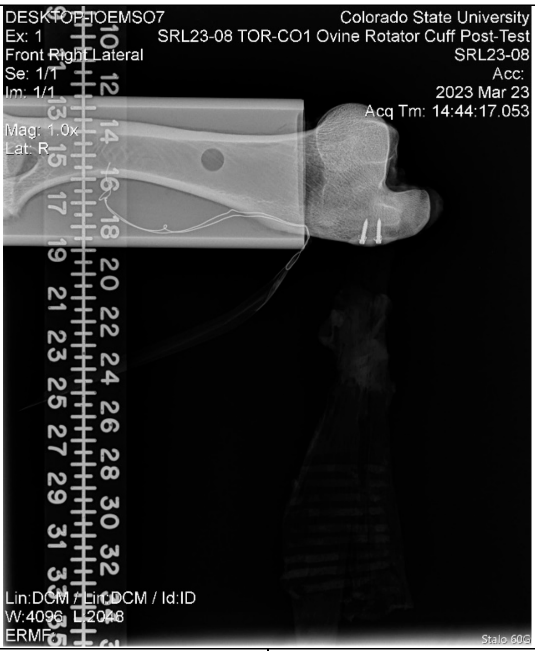
</td></tr>
<tr><th>Pre-Testing: M/L</th><th>Pre-Testing: D/V</th></tr>
<tr><td>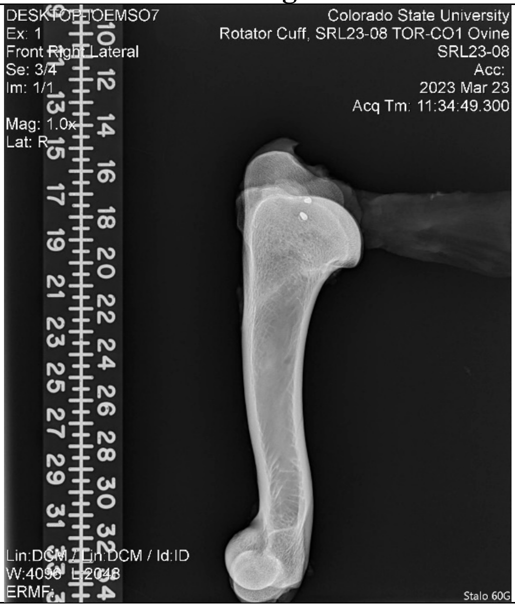
</td><td>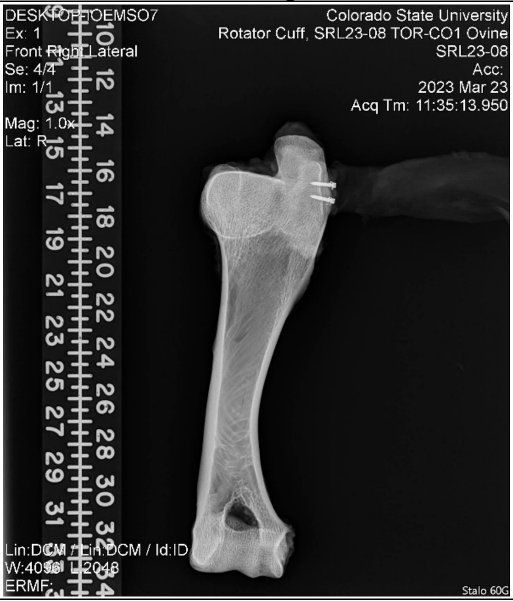
</td></tr>
</table>

<table>
<tr><th>Specimen Info</th><th colspan="2">Post-Testing</th></tr>
<tr><td rowspan="3">Sample ID:
TOR-CO2

Group:
Treatment

Timepoint:
Baseline</td><td colspan="2">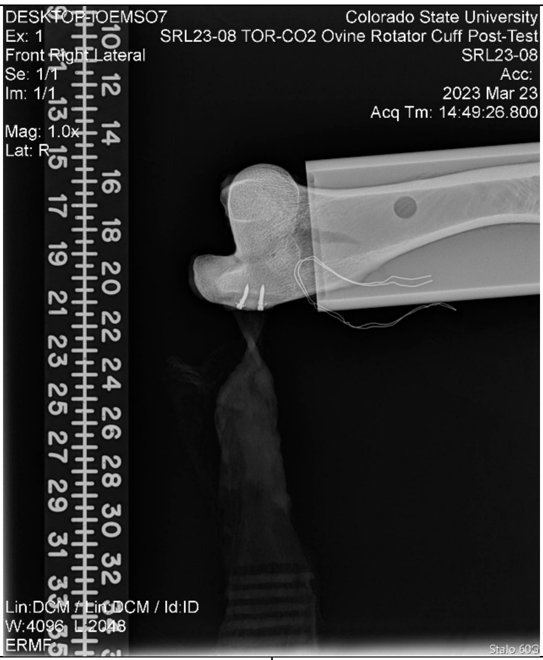
</td></tr>
<tr><th>Pre-Testing: M/L</th><th>Pre-Testing: D/V</th></tr>
<tr><td>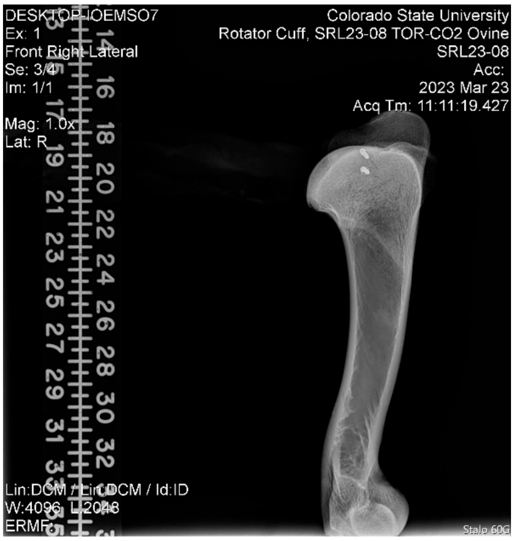
</td><td>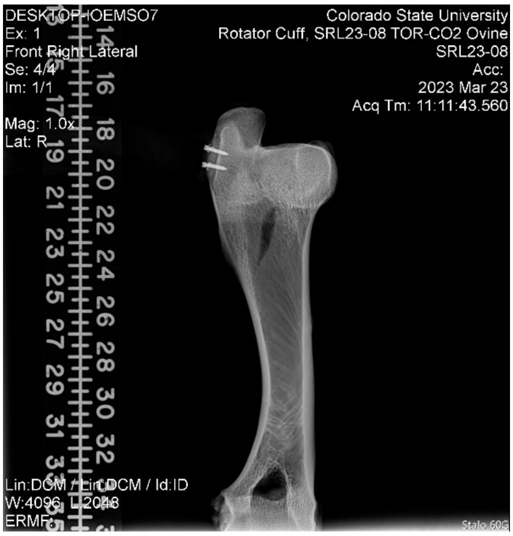
</td></tr>
</table>

<table>
<tr><th>Specimen Info</th><th colspan="2">Post-Testing</th></tr>
<tr><td rowspan="3">Sample ID:
TOR-CO3

Group:
Treatment

Timepoint:
Baseline</td><td colspan="2">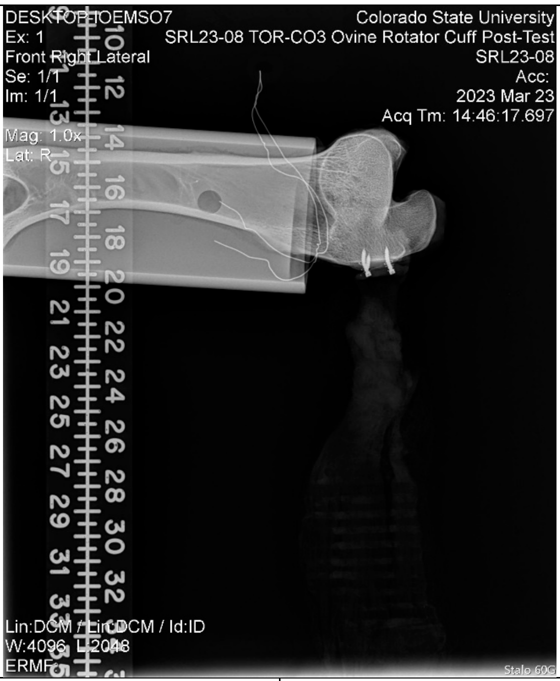
</td></tr>
<tr><th>Pre-Testing: M/L</th><th>Pre-Testing: D/V</th></tr>
<tr><td>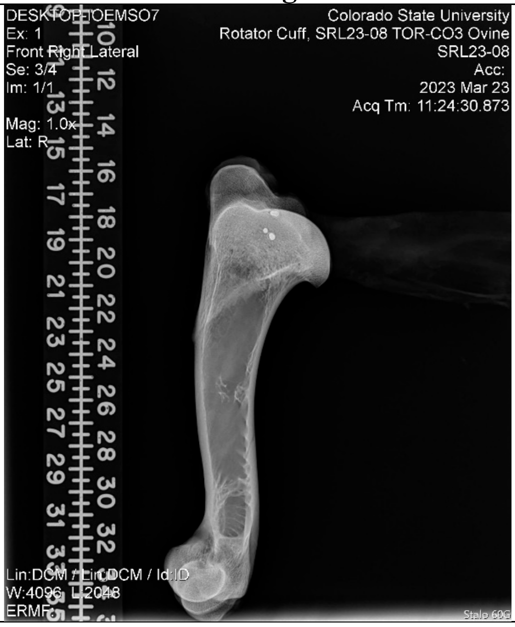
</td><td>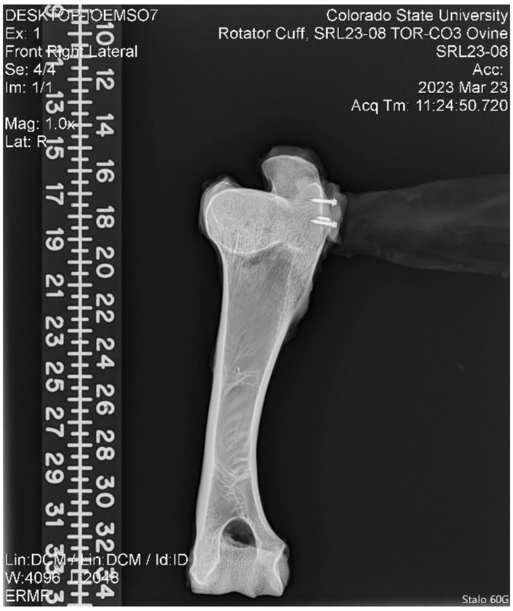
</td></tr>
</table>

<table>
<tr><th>Specimen Info</th><th colspan="2">Post-Testing</th></tr>
<tr><td rowspan="3">Sample ID:
TOR-CO4

Group:
Treatment

Timepoint:
Baseline</td><td colspan="2">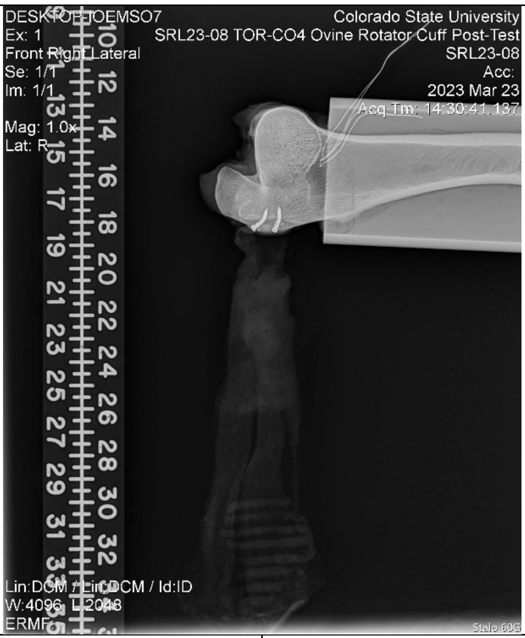
</td></tr>
<tr><th>Pre-Testing: M/L</th><th>Pre-Testing: D/V</th></tr>
<tr><td>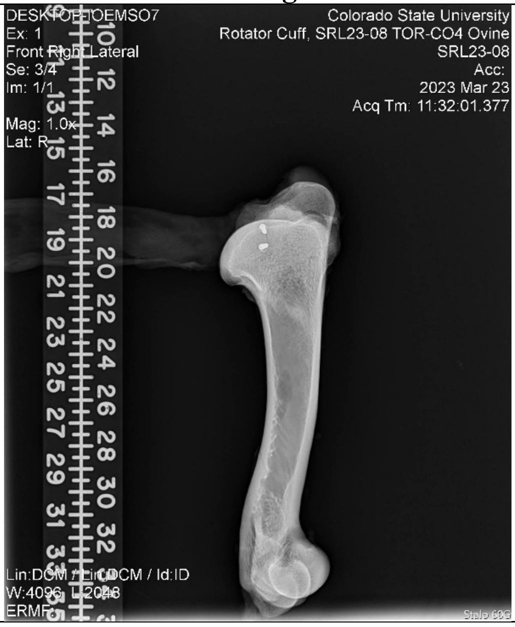
</td><td>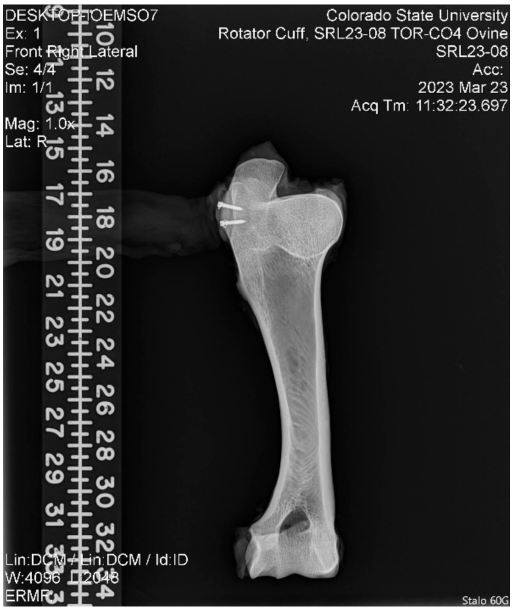
</td></tr>
</table>

<table>
<tr><th>Specimen Info</th><th colspan="2">Post-Testing</th></tr>
<tr><td rowspan="3">Sample ID:
TOR-CO5

Group:
Treatment

Timepoint:
Baseline</td><td colspan="2">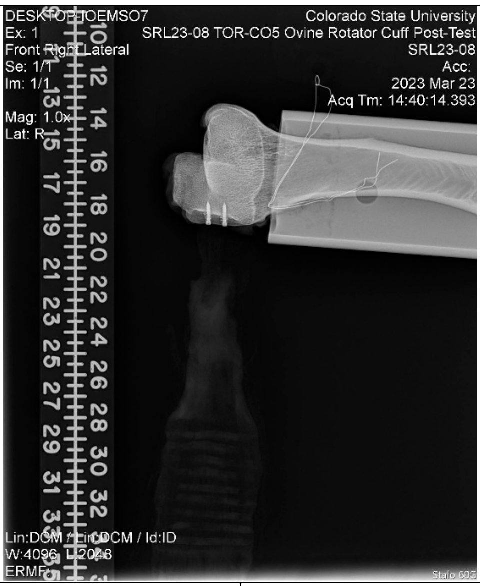
</td></tr>
<tr><th>Pre-Testing: M/L</th><th>Pre-Testing: D/V</th></tr>
<tr><td>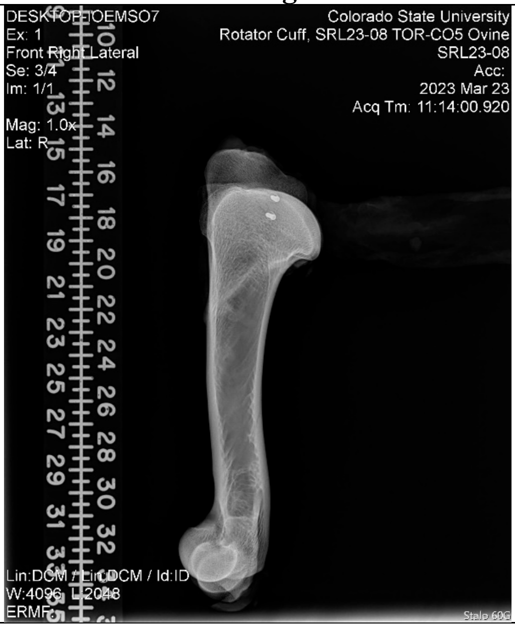
</td><td>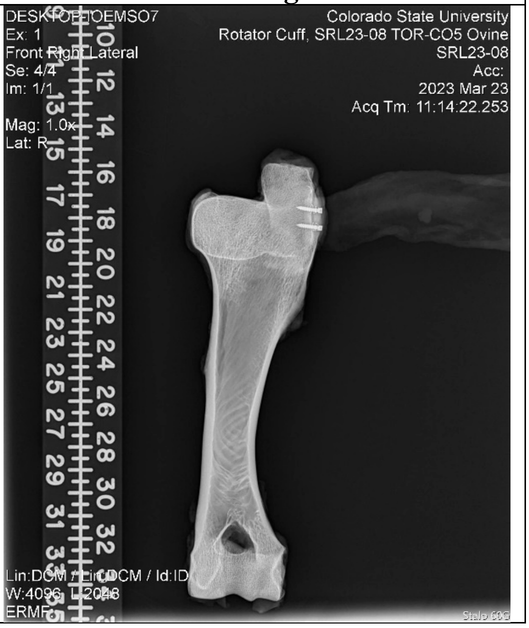
</td></tr>
</table>

<table>
<tr><th>Specimen Info</th><th colspan="2">Post-Testing</th></tr>
<tr><td rowspan="3">Sample ID:
TOR-CO6

Group:
Treatment

Timepoint:
Baseline</td><td colspan="2">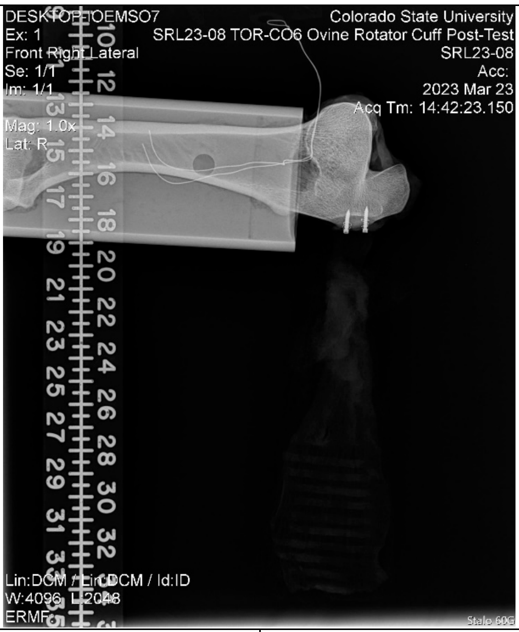
</td></tr>
<tr><th>Pre-Testing: M/L</th><th>Pre-Testing: D/V</th></tr>
<tr><td>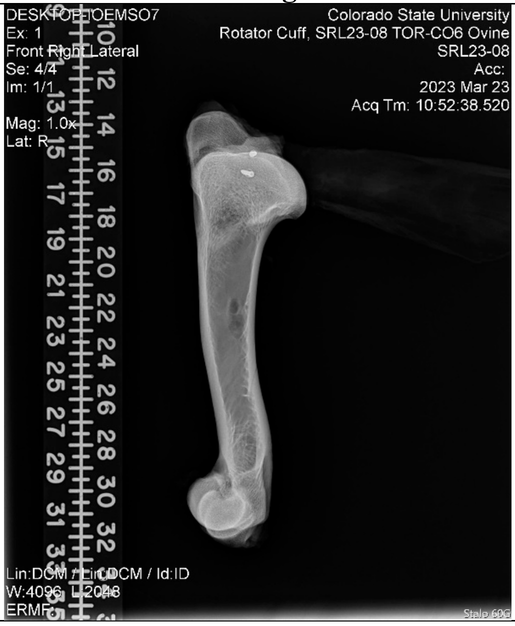
</td><td>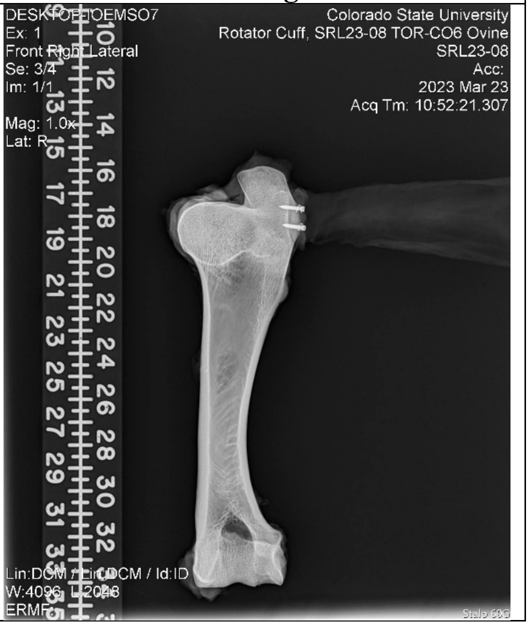
</td></tr>
</table>

Specimen Info	Post-Testing
**Sample ID:** TOR-CO7<br>**Group:** Treatment<br>**Timepoint:** Baseline	

Post-Testing

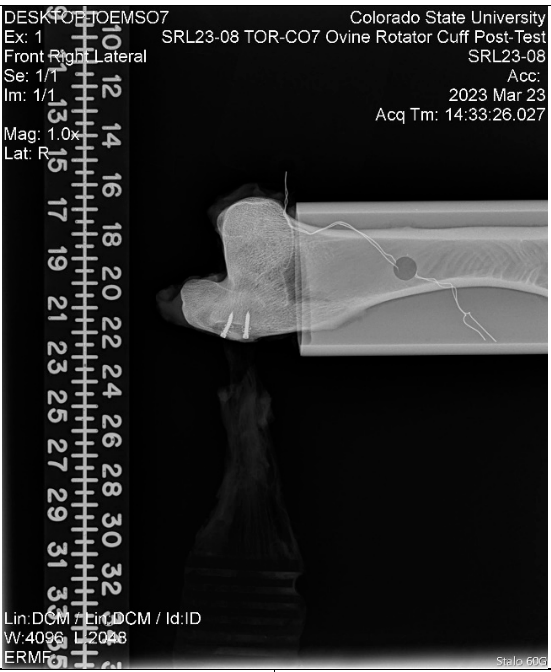

Pre-Testing: M/L

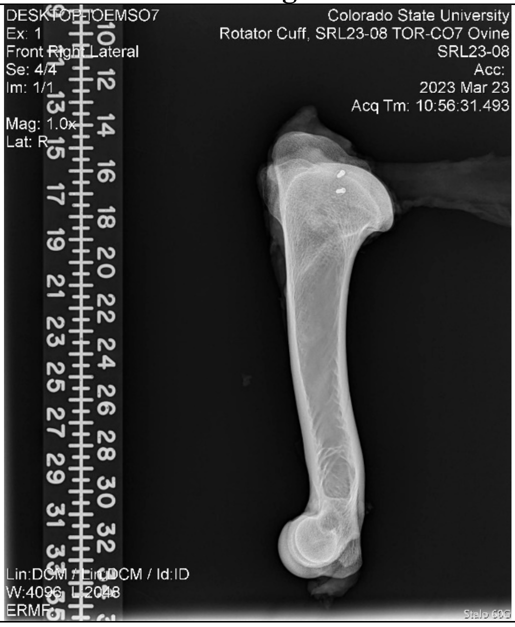

Pre-Testing: D/V

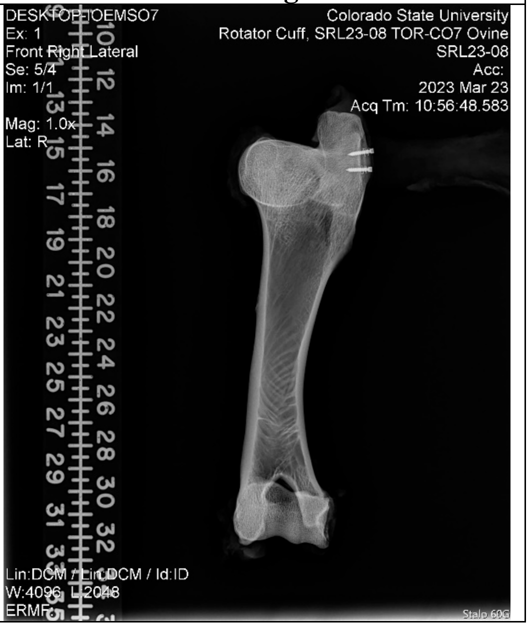

<table>
<tr><th>Specimen Info</th><th colspan="2">Post-Testing</th></tr>
<tr><td rowspan="3">Sample ID:
TOR-CO8

Group:
Treatment

Timepoint:
Baseline</td><td colspan="2">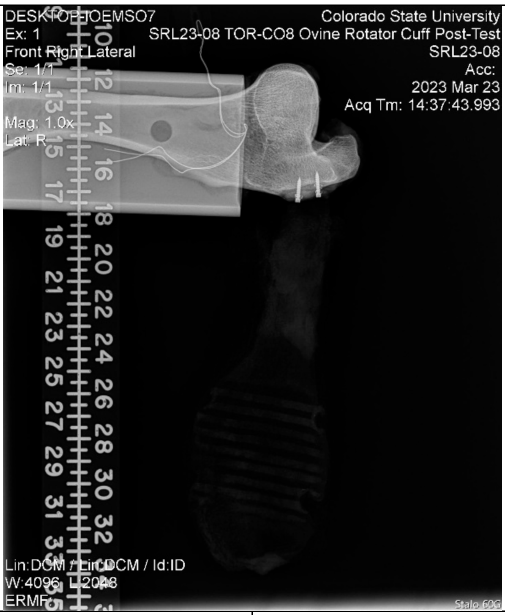
</td></tr>
<tr><th>Pre-Testing: M/L</th><th>Pre-Testing: D/V</th></tr>
<tr><td>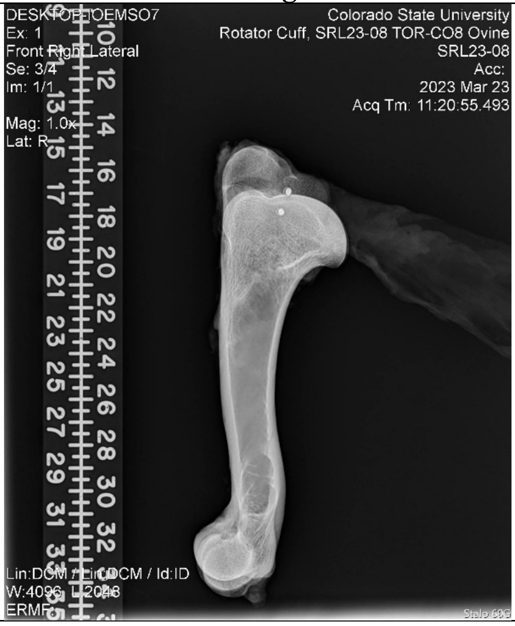
</td><td>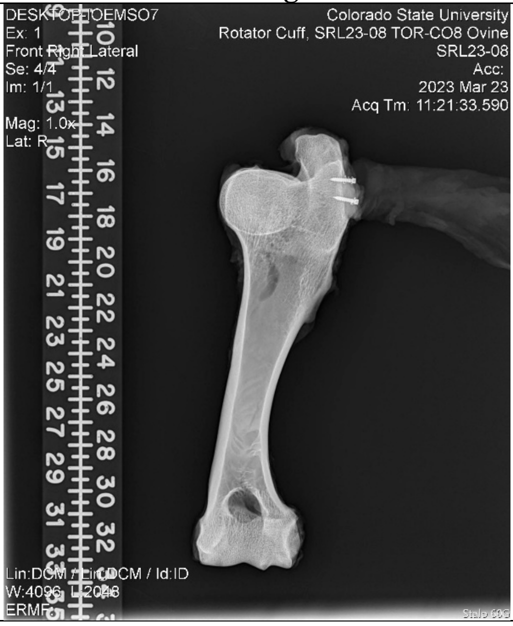
</td></tr>
</table>

<table>
<tr><th>Specimen Info</th><th colspan="2">Post-Testing</th></tr>
<tr><td rowspan="3">Sample ID:
TOR-CO9

Group:
Treatment

Timepoint:
Baseline</td><td colspan="2">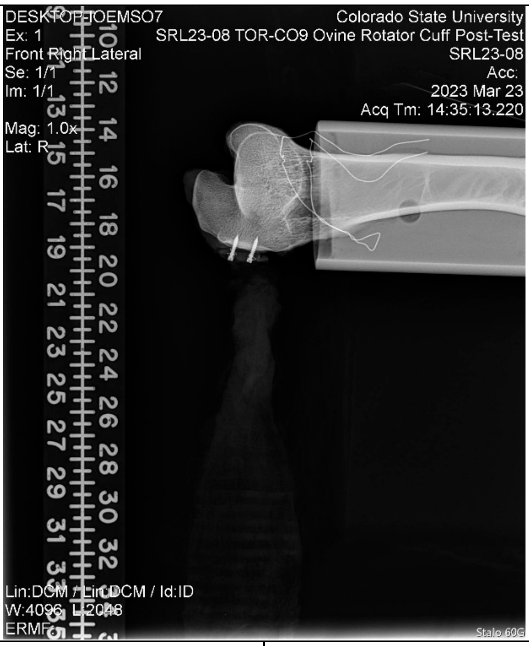
</td></tr>
<tr><th>Pre-Testing: M/L</th><th>Pre-Testing: D/V</th></tr>
<tr><td>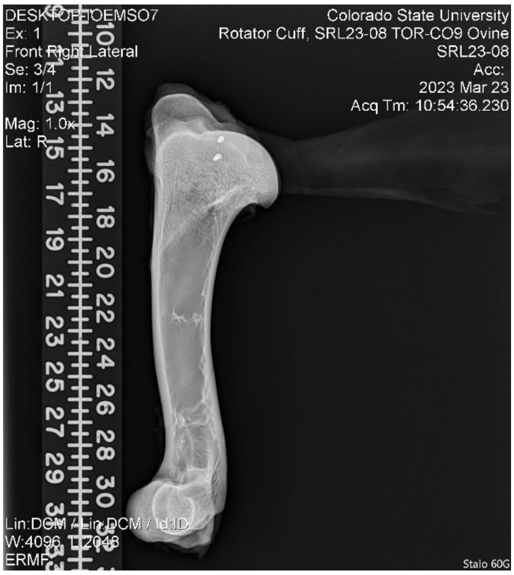
</td><td>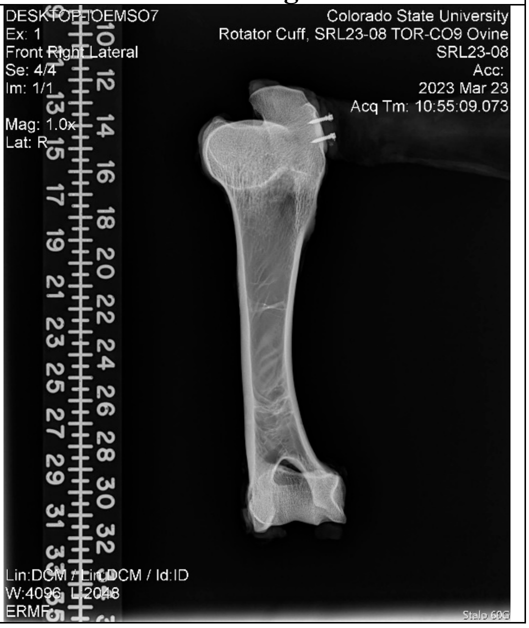
</td></tr>
</table>

<table>
<tr><th>Specimen Info</th><th colspan="2">Post-Testing</th></tr>
<tr><td rowspan="4">Sample ID:
TOR-C10

Group:
Treatment

Timepoint:
Control</td><td colspan="2">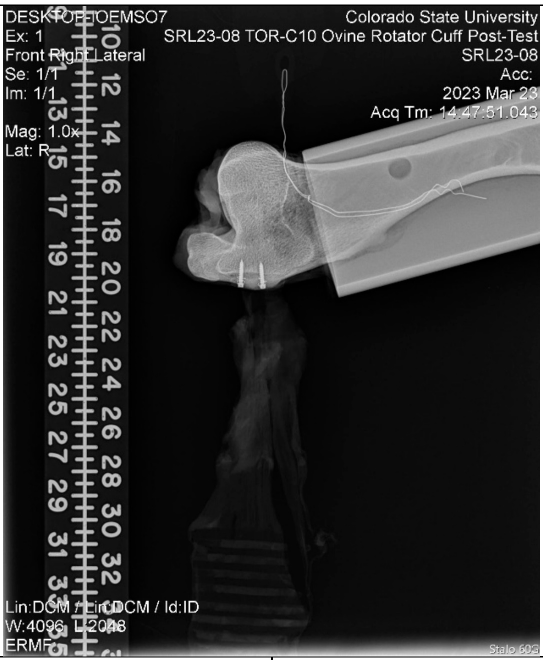
</td></tr>
<tr><th>Pre-Testing: M/L</th><th>Pre-Testing: D/V</th></tr>
<tr><td>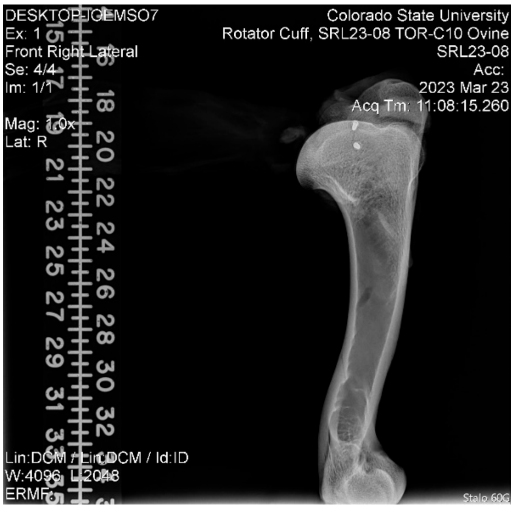
</td><td>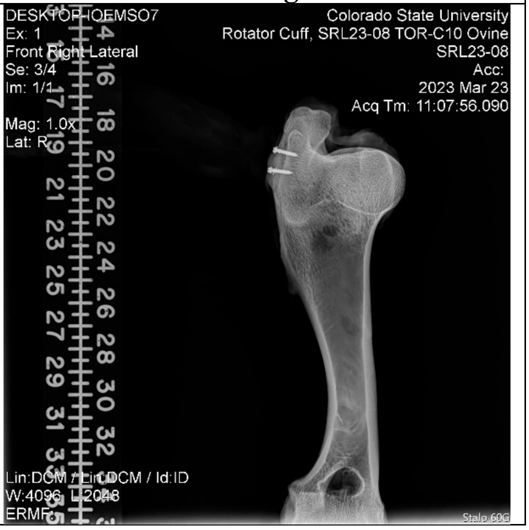
</td></tr>
</table>

<table>
<tr><th>Specimen Info</th><th colspan="2">Post-Testing</th></tr>
<tr><td rowspan="4">Sample ID:
TOR-C11

Group:
Treatment

Timepoint:
Control</td><td colspan="2">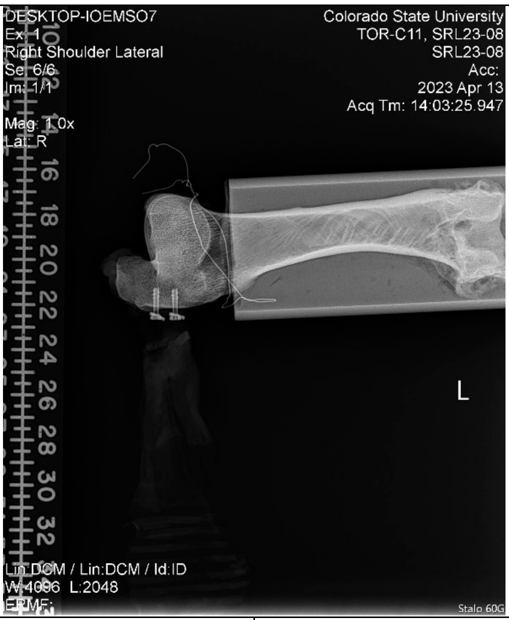
</td></tr>
<tr><th>Pre-Testing: M/L</th><th>Pre-Testing: D/V</th></tr>
<tr><td>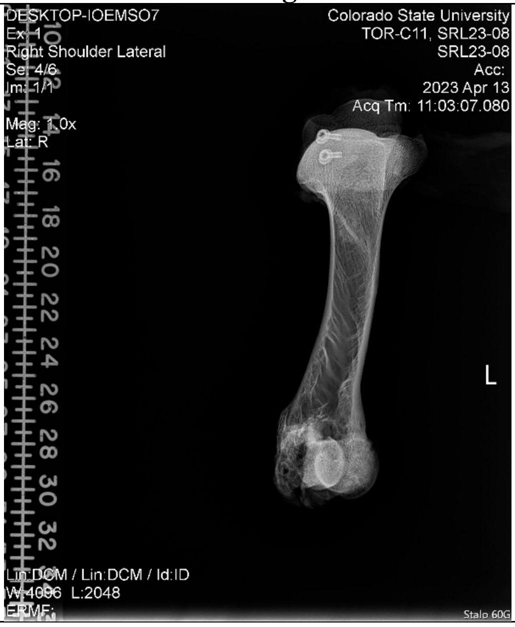
</td><td>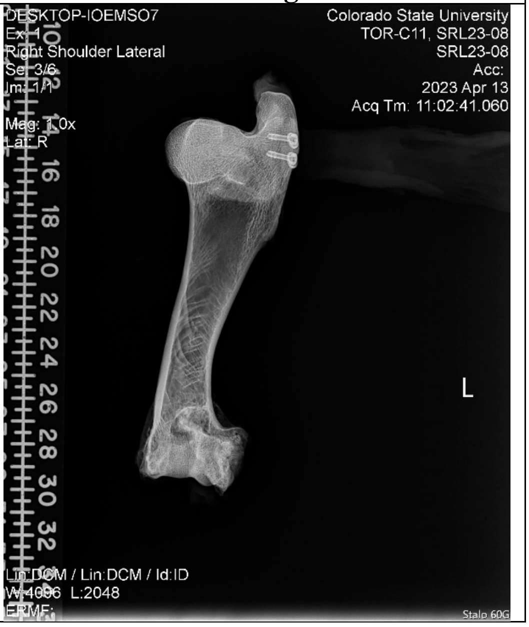
</td></tr>
</table>

Specimen Info	Post-Testing
**Sample ID:** TOR-C12<br>**Group:** Treatment<br>**Timepoint:** Control	See post-testing radiograph below

Specimen Info

Sample ID:
TOR-C12

Group:
Treatment

Timepoint:
Control

Post-Testing

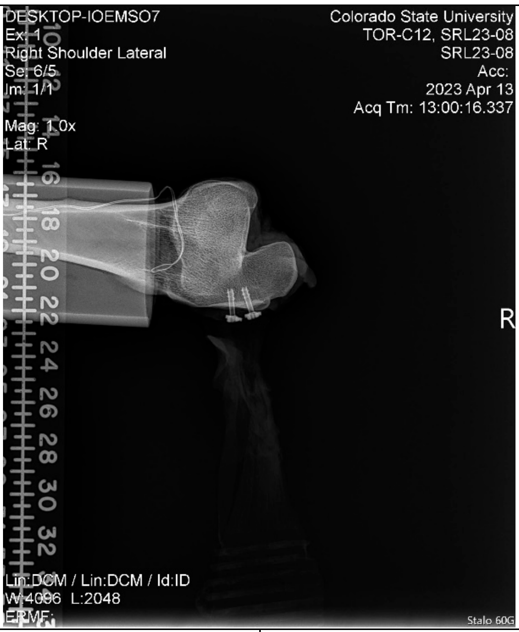

Pre-Testing: M/L

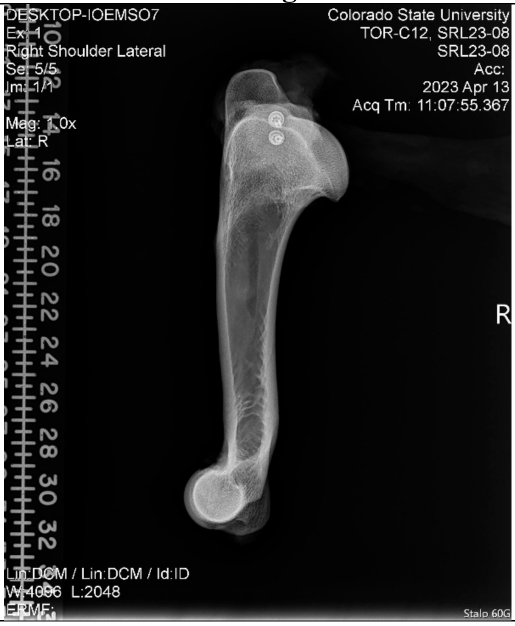

Pre-Testing: D/V

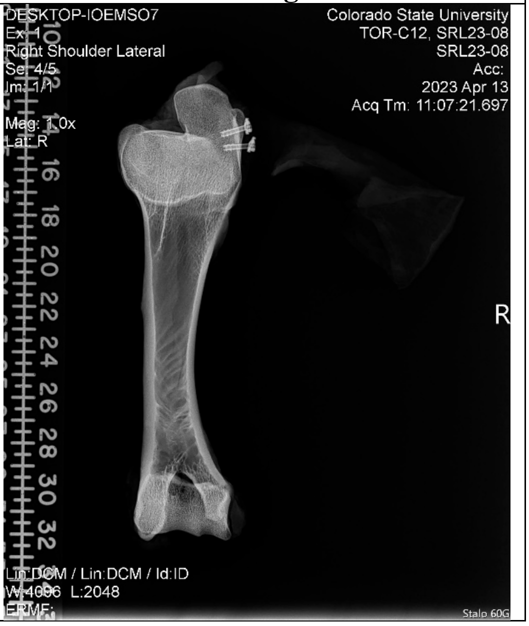

<table>
<tr><th>Specimen Info</th><th colspan="2">Post-Testing</th></tr>
<tr><td rowspan="3">Sample ID:
TOR-C13

Group:
Treatment

Timepoint:
Control</td><td colspan="2">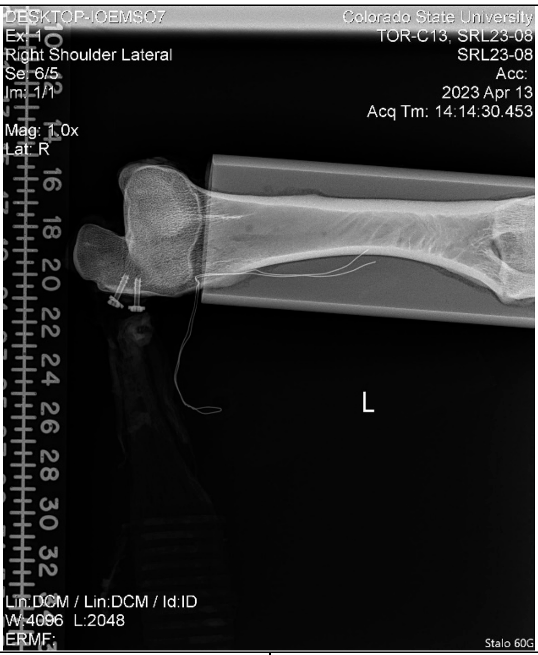
</td></tr>
<tr><th>Pre-Testing: M/L</th><th>Pre-Testing: D/V</th></tr>
<tr><td>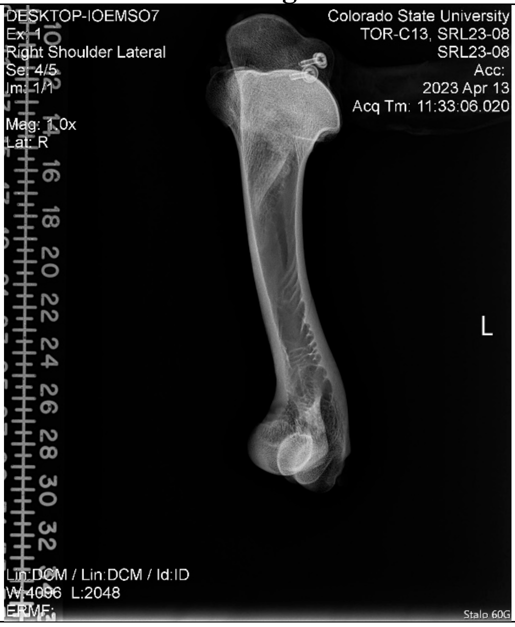
</td><td>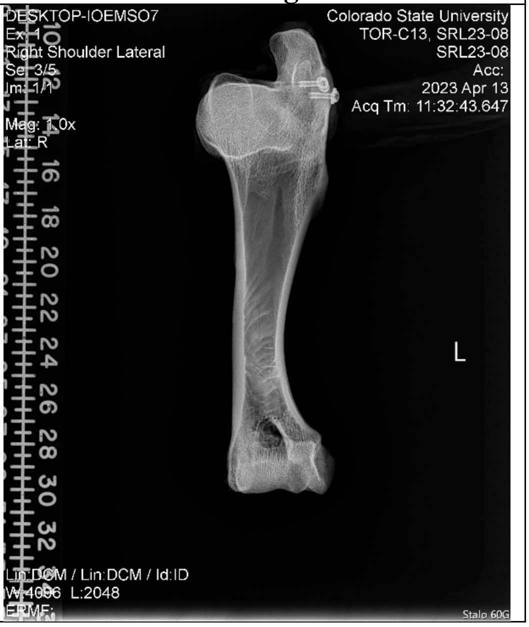
</td></tr>
</table>

<table>
<tr><th>Specimen Info</th><th colspan="2">Post-Testing</th></tr>
<tr><td rowspan="3">Sample ID:
TOR-C14

Group:
Treatment

Timepoint:
Control</td><td colspan="2">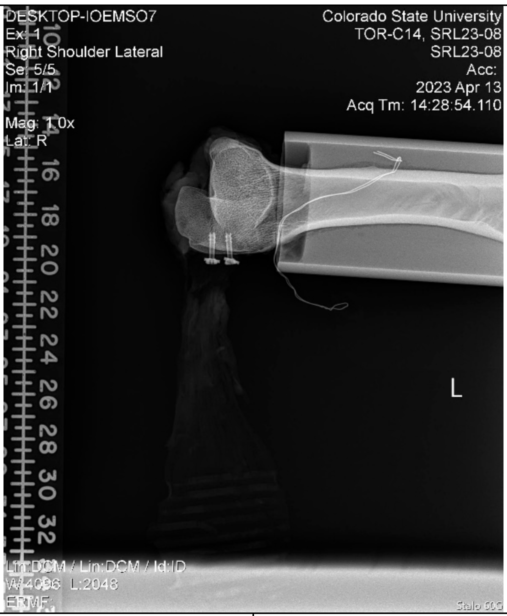
</td></tr>
<tr><th>Pre-Testing: M/L</th><th>Pre-Testing: D/V</th></tr>
<tr><td>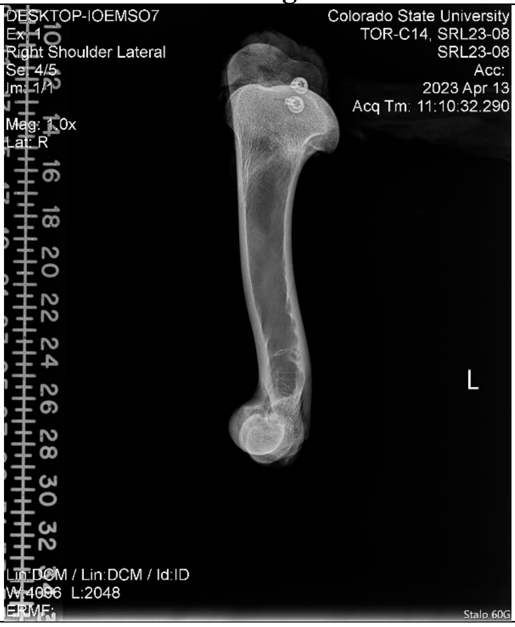
</td><td>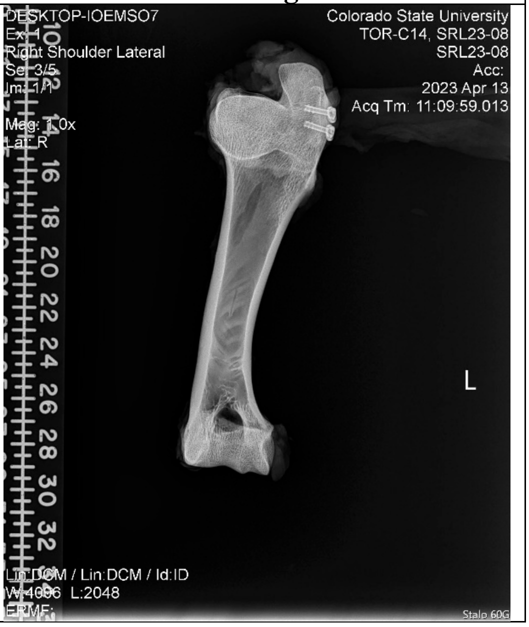
</td></tr>
</table>

<table>
<tr><th>Specimen Info</th><th colspan="2">Post-Testing</th></tr>
<tr><td rowspan="4">Sample ID:
TOR-C15

Group:
Treatment

Timepoint:
Control</td><td colspan="2">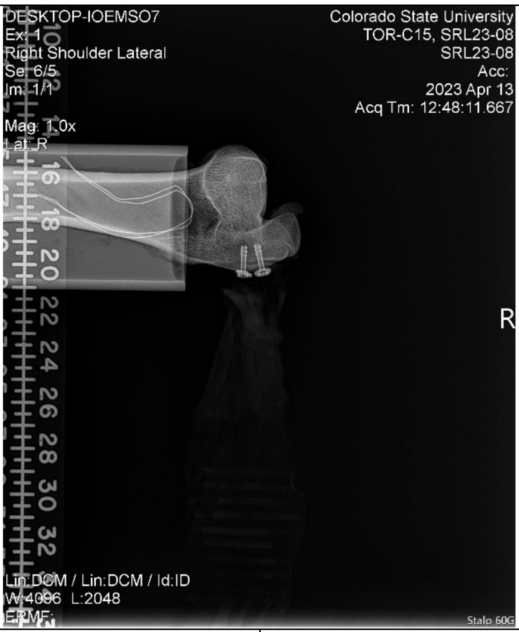
</td></tr>
<tr><th>Pre-Testing: M/L</th><th>Pre-Testing: D/V</th></tr>
<tr><td>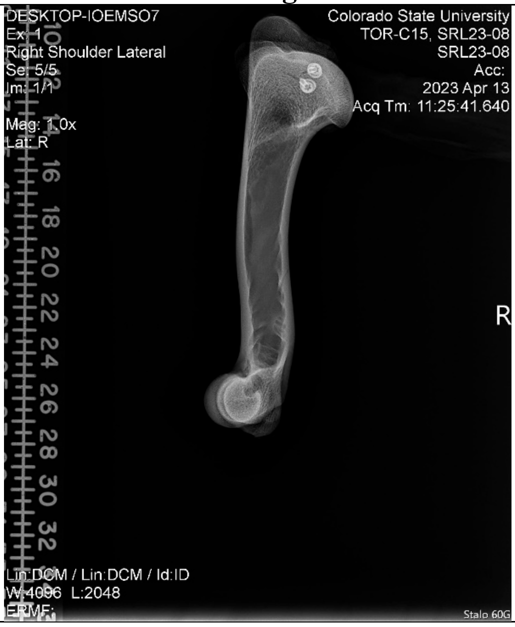
</td><td>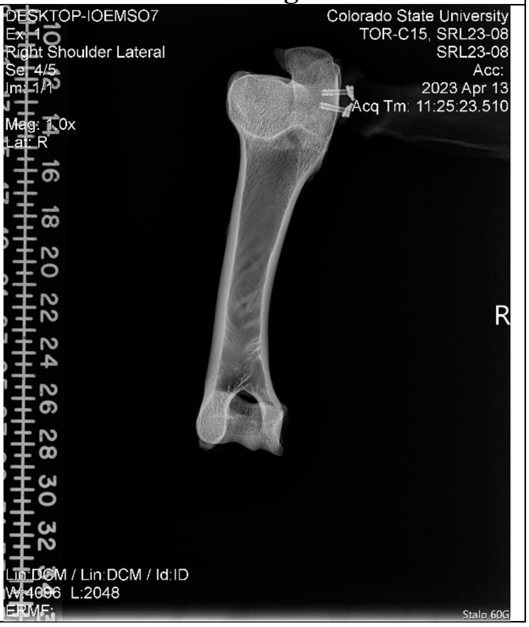
</td></tr>
</table>

<table>
<tr><th>Specimen Info</th><th colspan="2">Post-Testing</th></tr>
<tr><td rowspan="3">Sample ID:
TOR-C16

Group:
Treatment

Timepoint:
Control</td><td colspan="2">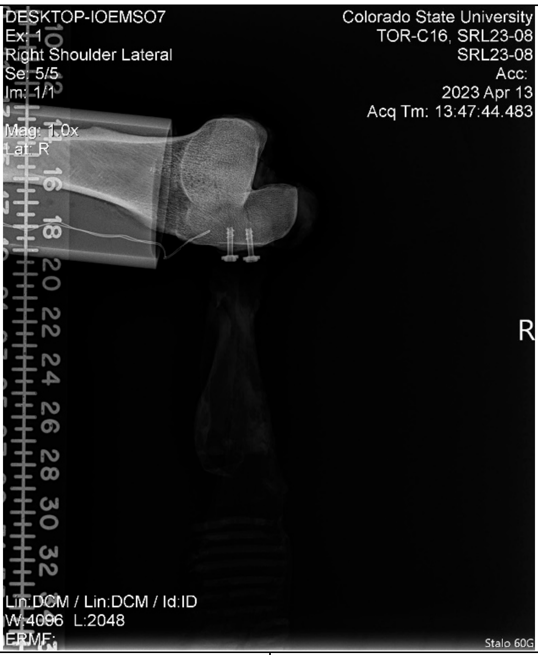</td></tr>
<tr><th>Pre-Testing: M/L</th><th>Pre-Testing: D/V</th></tr>
<tr><td>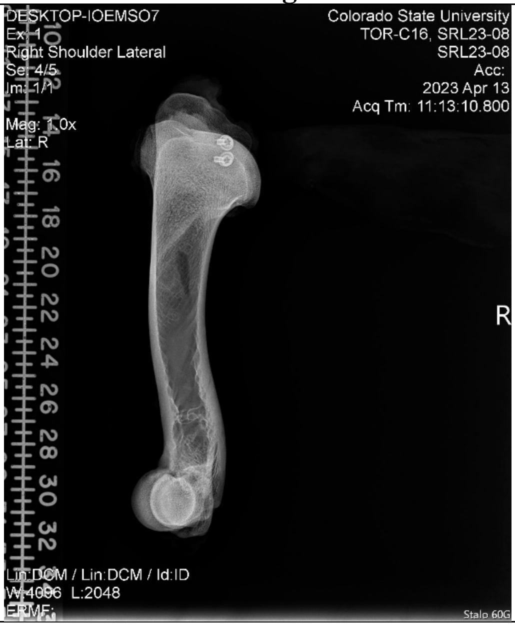</td><td>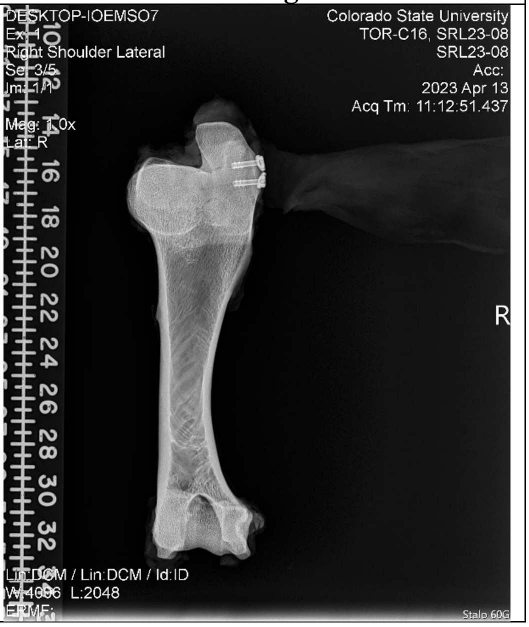</td></tr>
</table>

<table>
<tr><th>Specimen Info</th><th colspan="2">Post-Testing</th></tr>
<tr><td rowspan="4">Sample ID:
TOR-C17

Group:
Treatment

Timepoint:
Control</td><td colspan="2">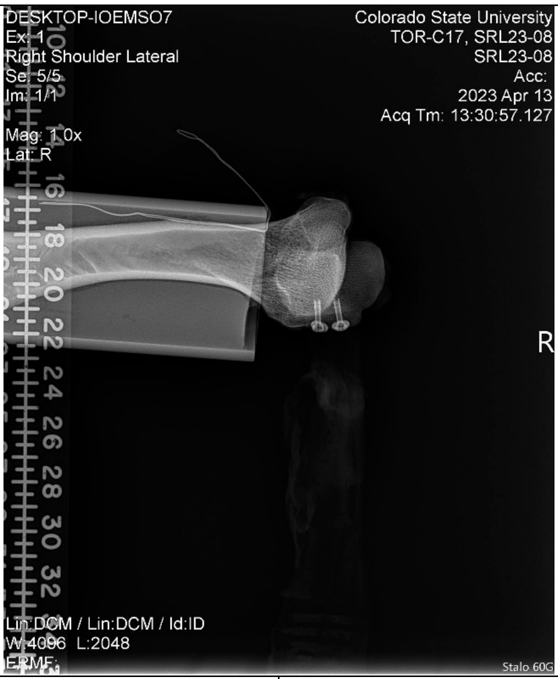
</td></tr>
<tr><th>Pre-Testing: M/L</th><th>Pre-Testing: D/V</th></tr>
<tr><td>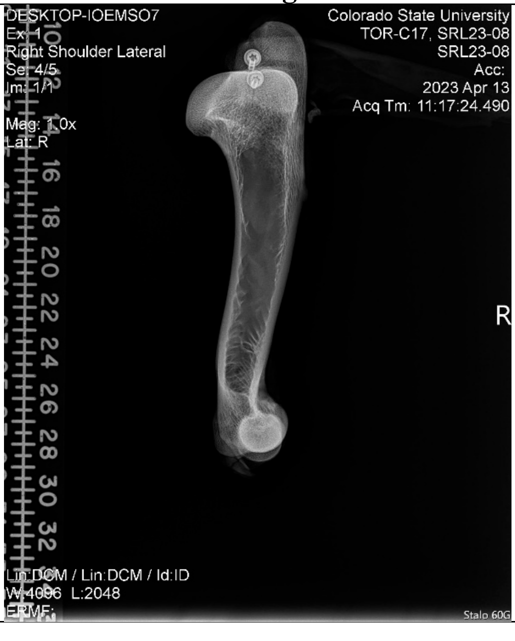
</td><td>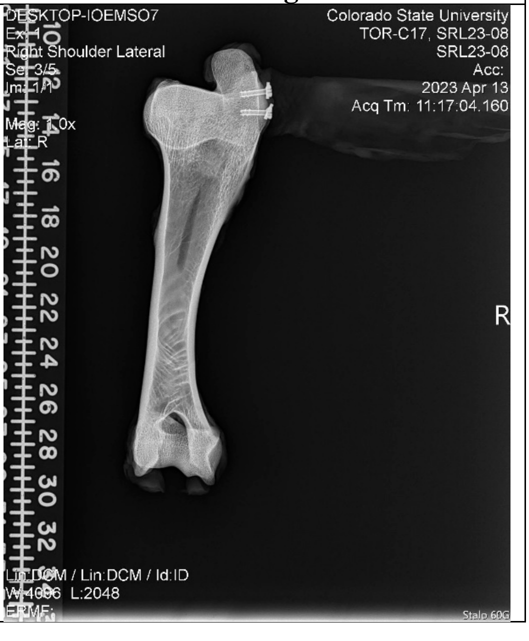
</td></tr>
</table>

<table>
<tr><th>Specimen Info</th><th colspan="2">Post-Testing</th></tr>
<tr><td rowspan="3">Sample ID:
TOR-C18

Group:
Treatment

Timepoint:
Control</td><td colspan="2">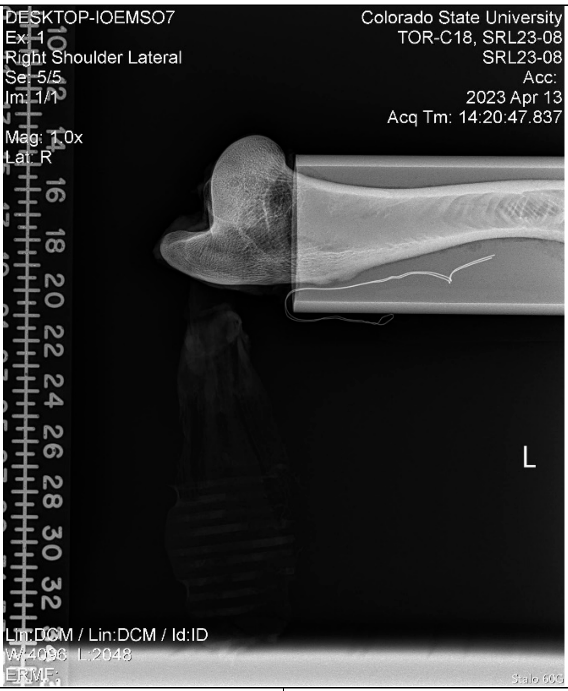
</td></tr>
<tr><th>Pre-Testing: M/L</th><th>Pre-Testing: D/V</th></tr>
<tr><td>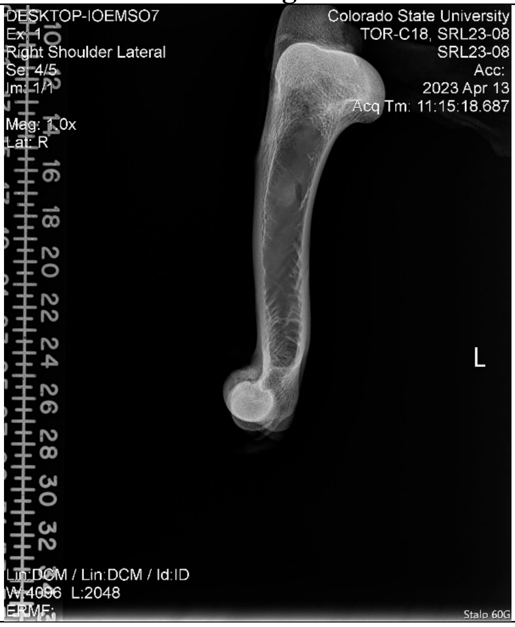
</td><td>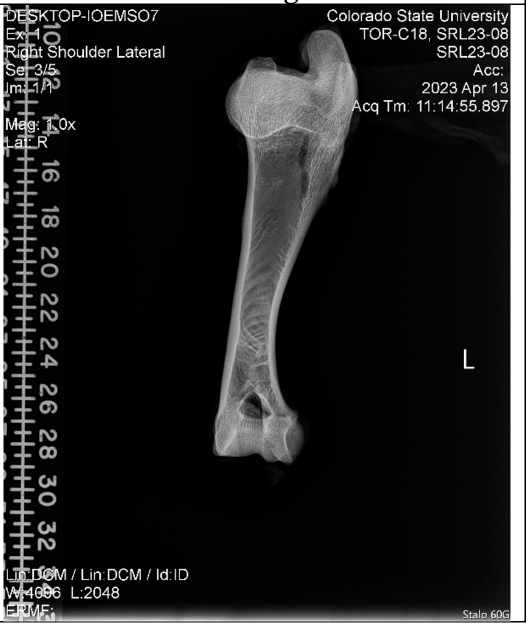
</td></tr>
</table>

<table>
<tr><th>Specimen Info</th><th colspan="2">Post-Testing</th></tr>
<tr><td rowspan="3">Sample ID:
TOR-C19

Group:
Empty

Timepoint:
Baseline</td><td colspan="2">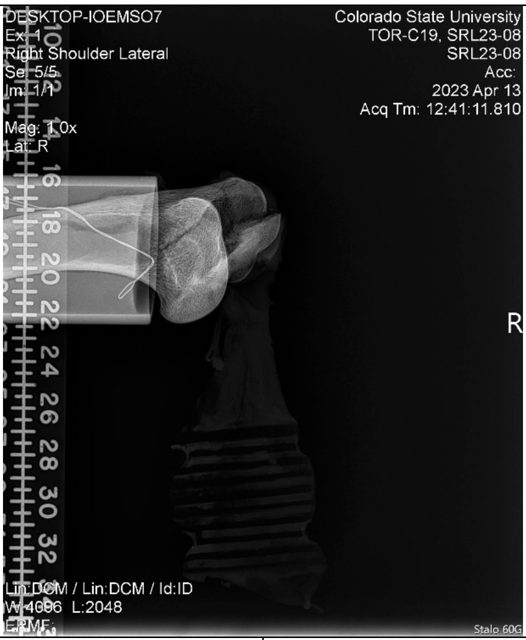
</td></tr>
<tr><th>Pre-Testing: M/L</th><th>Pre-Testing: D/V</th></tr>
<tr><td>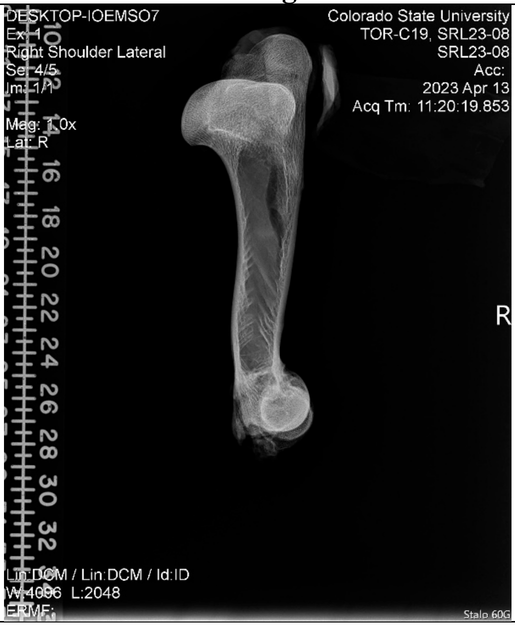
</td><td>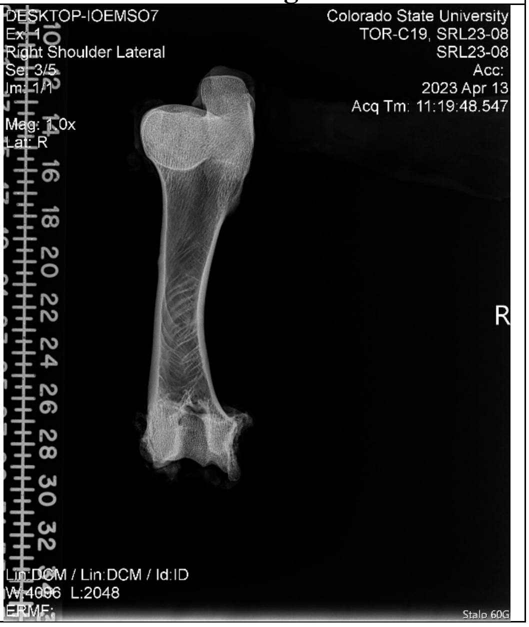
</td></tr>
</table>

<table>
<tr><th>Specimen Info</th><th colspan="2">Post-Testing</th></tr>
<tr><td rowspan="3">Sample ID:
TOR-C20

Group:
Empty

Timepoint:
Baseline</td><td colspan="2">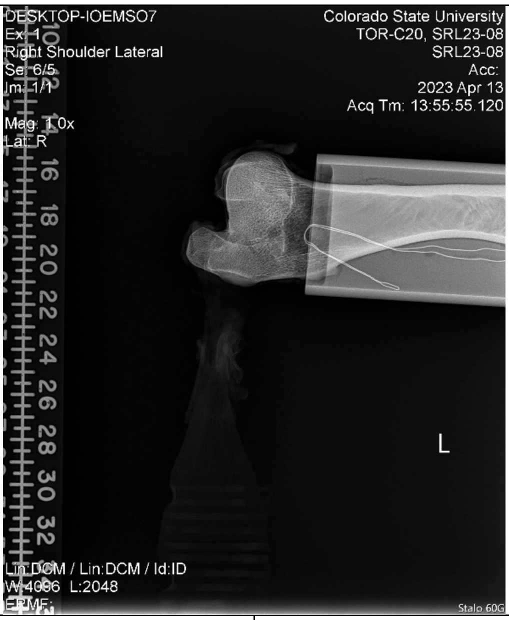
</td></tr>
<tr><th>Pre-Testing: M/L</th><th>Pre-Testing: D/V</th></tr>
<tr><td>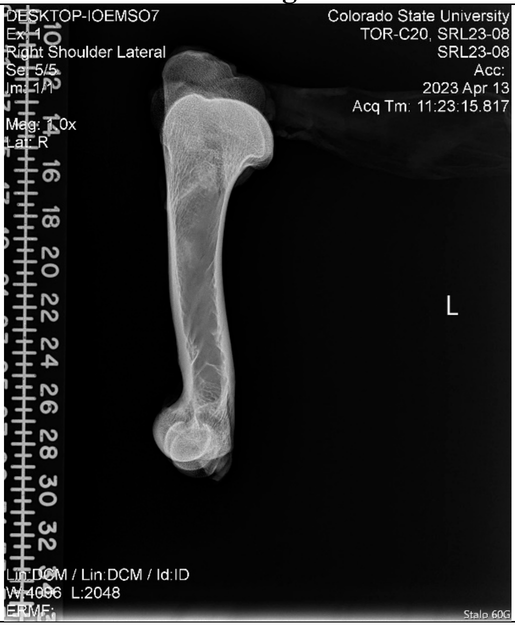
</td><td>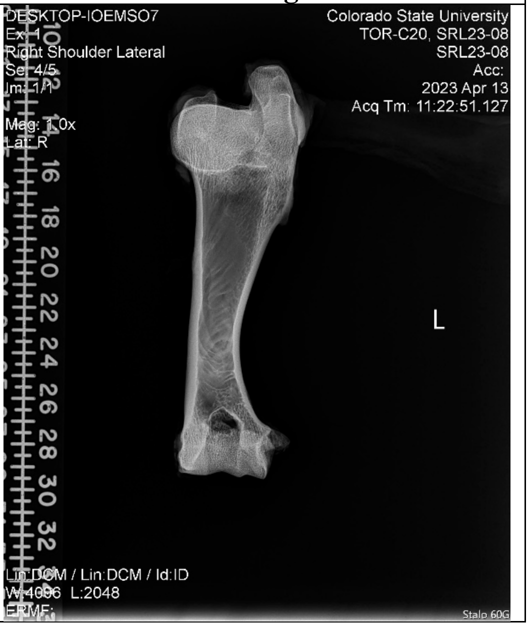
</td></tr>
</table>

Supplementary File C

APPENDIX C – IN VIVO POST DISSECTION IMAGES

<table>
<tr><th>Specimen Info</th><th>Dorsal</th><th>Lateral</th></tr>
<tr><td rowspan="5">Sample ID:
TOR01 L

Group:
Control

Timepoint:
8 weeks</td><td>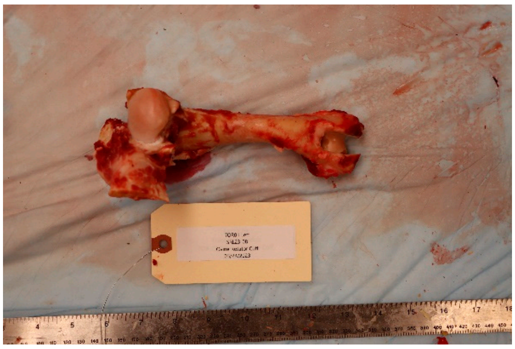</td><td>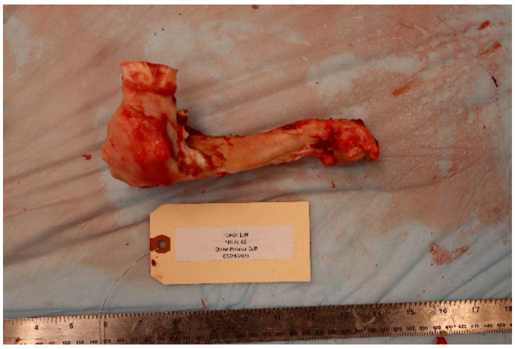</td></tr>
<tr><th>Ventral</th><th>Medial</th></tr>
<tr><td>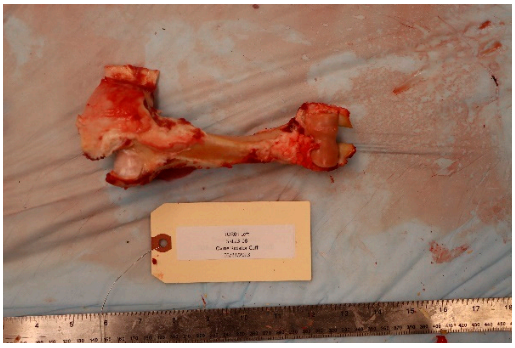</td><td>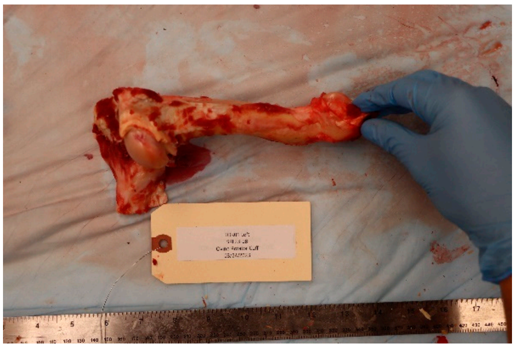</td></tr>
<tr><th colspan="2">Axial</th></tr>
<tr><td colspan="2">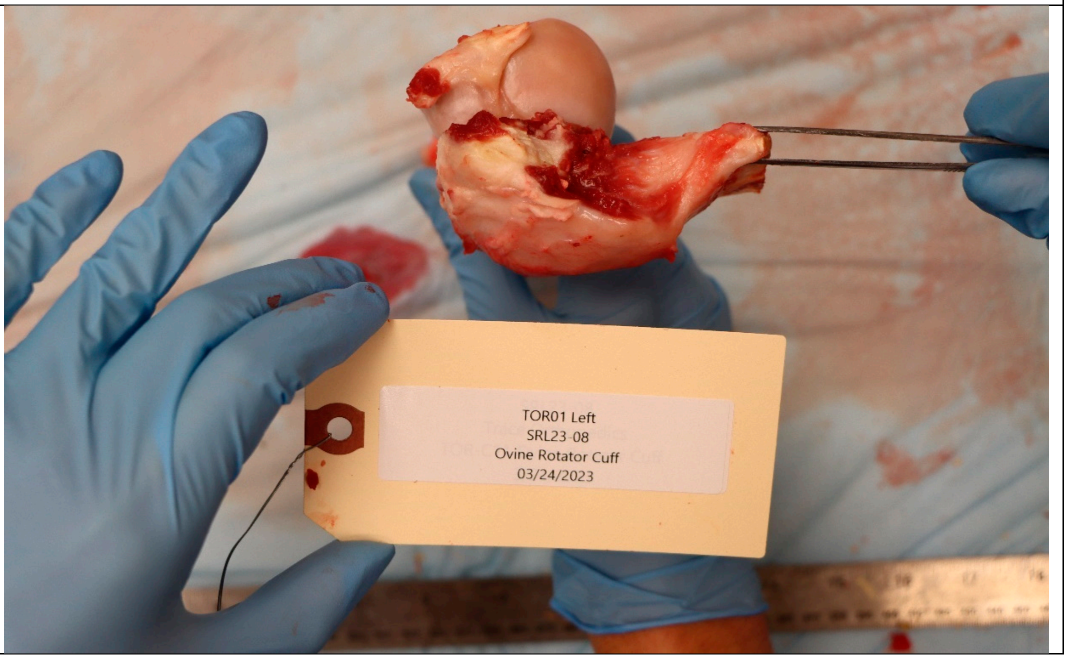
</td></tr>
</table>

<table>
<tr><th>Specimen Info</th><th>Dorsal</th><th>Lateral</th></tr>
<tr><td rowspan="5">Sample ID:
TOR01 R

Group:
Treatment

Timepoint:
8 weeks</td><td>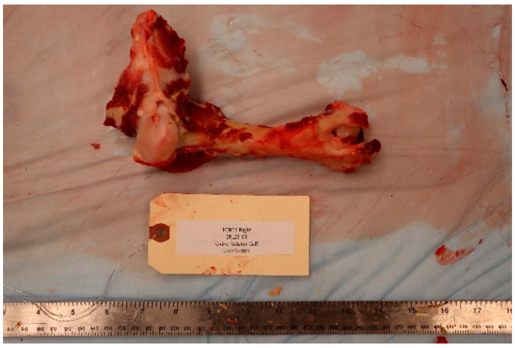</td><td>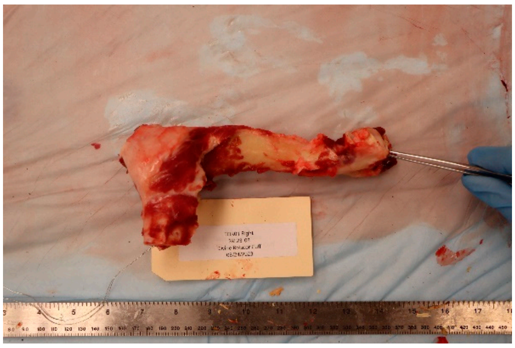</td></tr>
<tr><th>Ventral</th><th>Medial</th></tr>
<tr><td>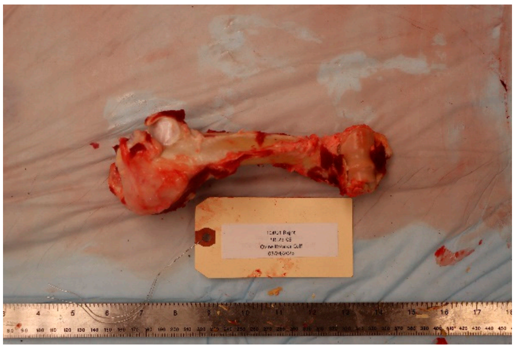</td><td>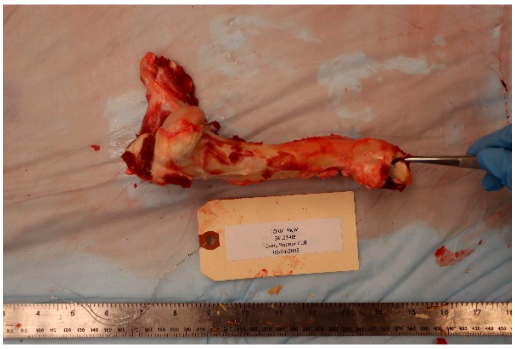</td></tr>
<tr><th colspan="2">Axial</th></tr>
<tr><td colspan="2">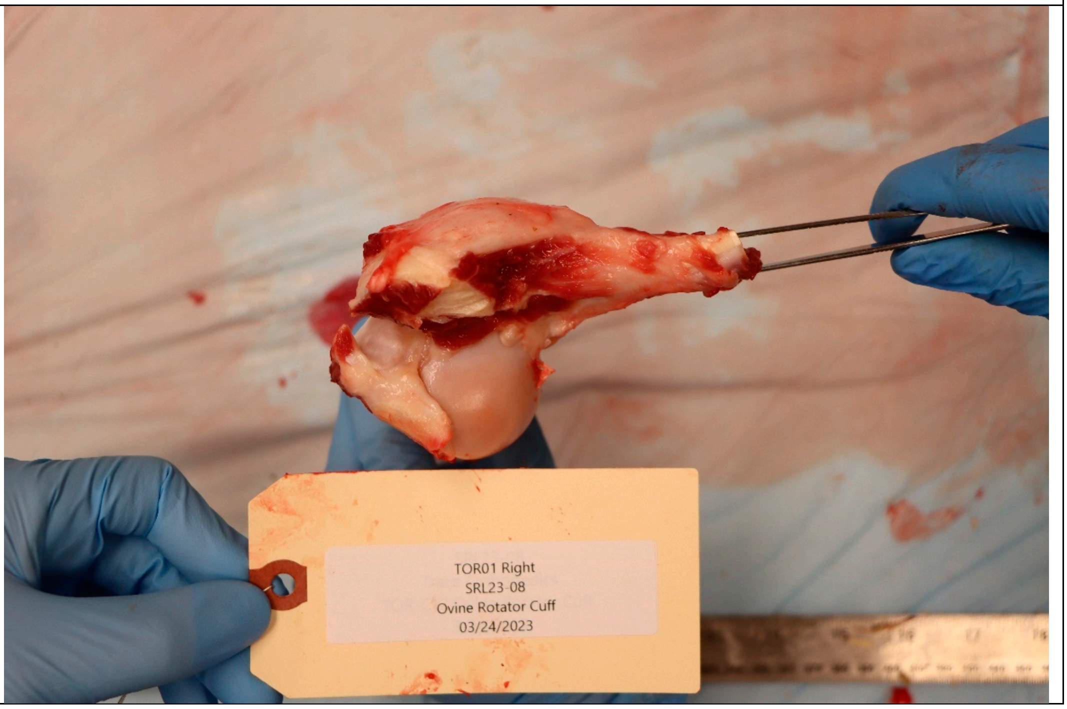
</td></tr>
</table>

<table>
<tr><th>Specimen Info</th><th>Dorsal</th><th>Lateral</th></tr>
<tr><td rowspan="5">Sample ID:
TOR02 L

Group:
Control

Timepoint:
8 weeks</td><td>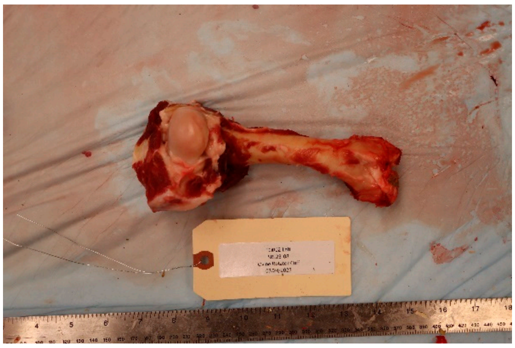</td><td>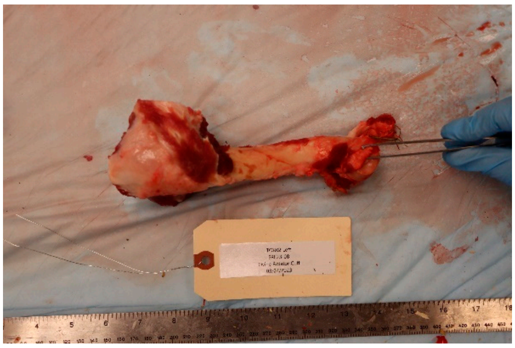</td></tr>
<tr><th>Ventral</th><th>Medial</th></tr>
<tr><td>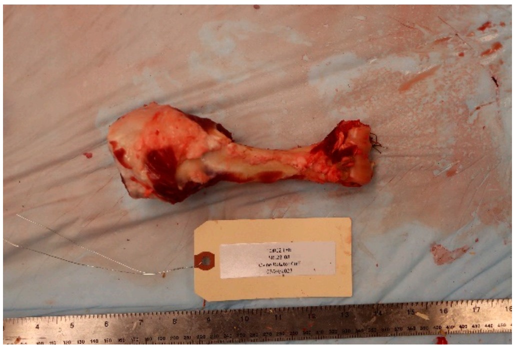</td><td>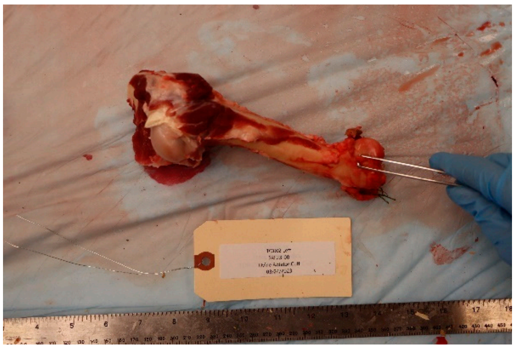</td></tr>
<tr><th colspan="2">Axial</th></tr>
<tr><td colspan="2">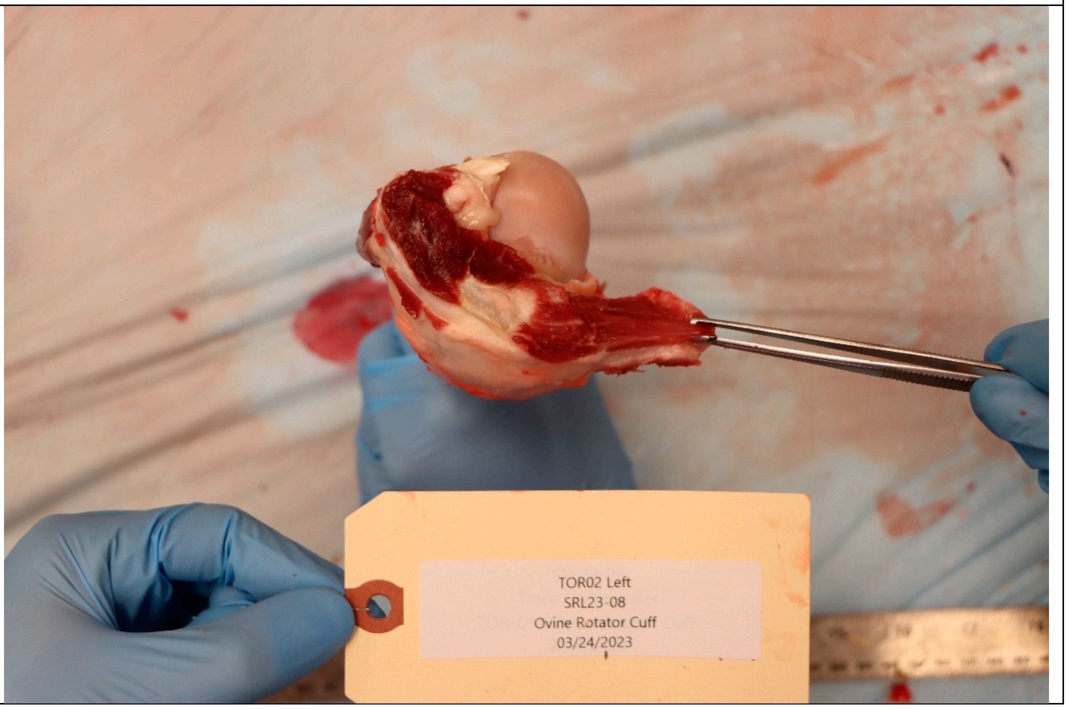
</td></tr>
</table>

<table>
<tr><th>Specimen Info</th><th>Dorsal</th><th>Lateral</th></tr>
<tr><td rowspan="5">Sample ID:
TOR02 R

Group:
Treatment

Timepoint:
8 weeks</td><td>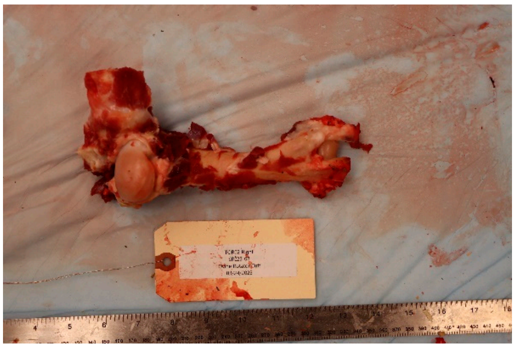</td><td>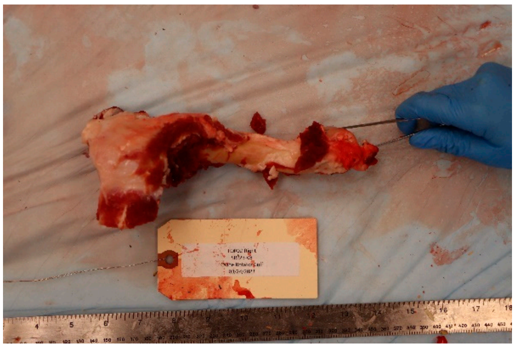</td></tr>
<tr><th>Ventral</th><th>Medial</th></tr>
<tr><td>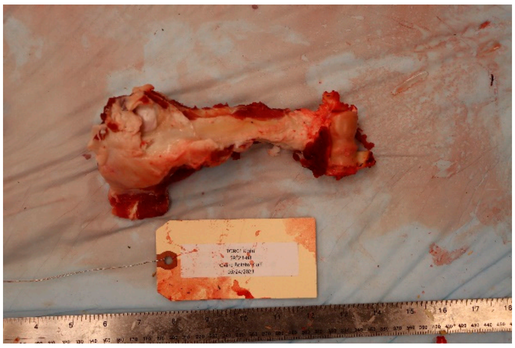</td><td>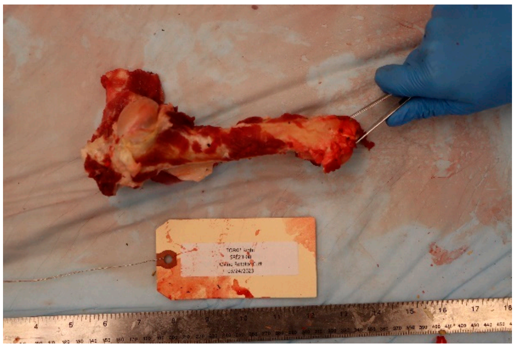</td></tr>
<tr><th colspan="2">Axial</th></tr>
<tr><td colspan="2">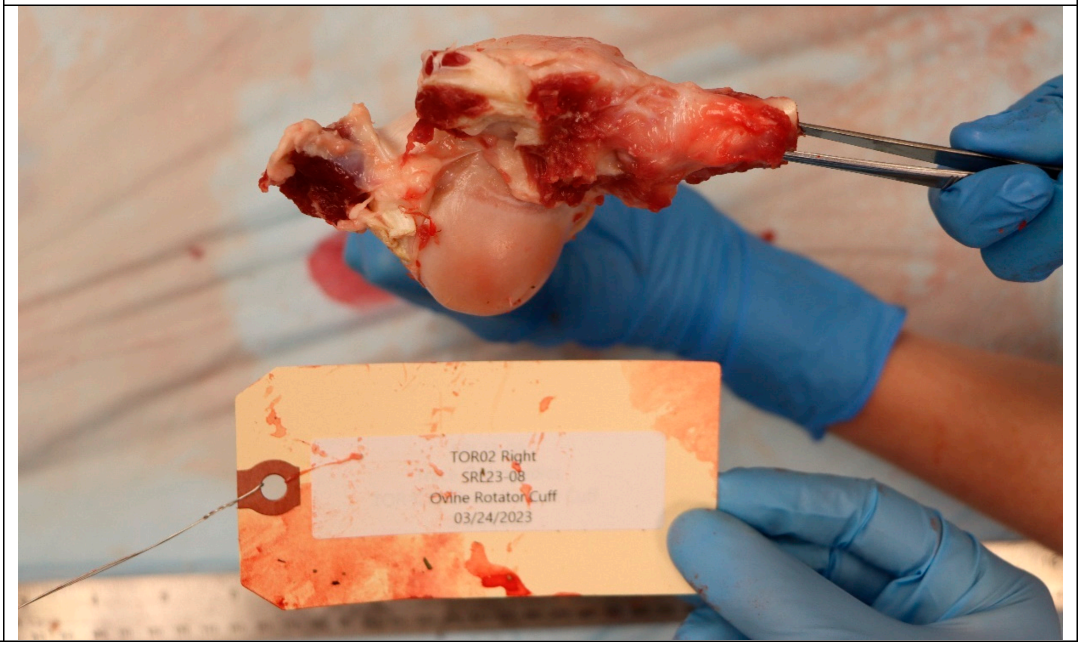
</td></tr>
</table>

<table>
<tr><th>Specimen Info</th><th>Dorsal</th><th>Lateral</th></tr>
<tr><td rowspan="5">Sample ID:
TOR03 L

Group:
Control

Timepoint:
8 weeks</td><td>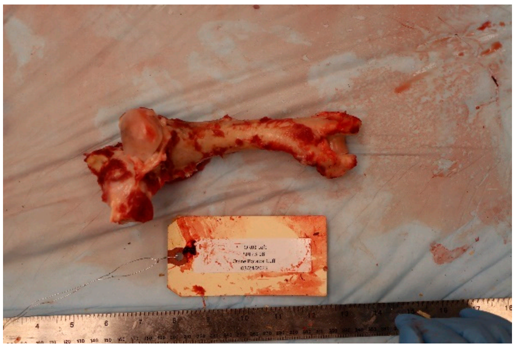</td><td>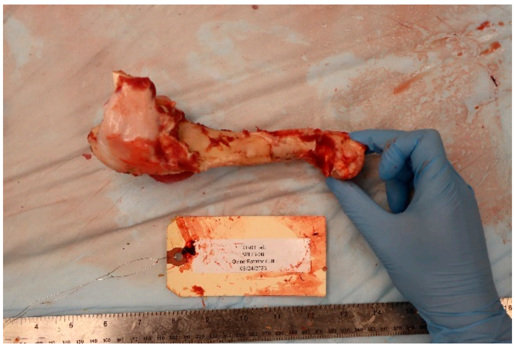</td></tr>
<tr><th>Ventral</th><th>Medial</th></tr>
<tr><td>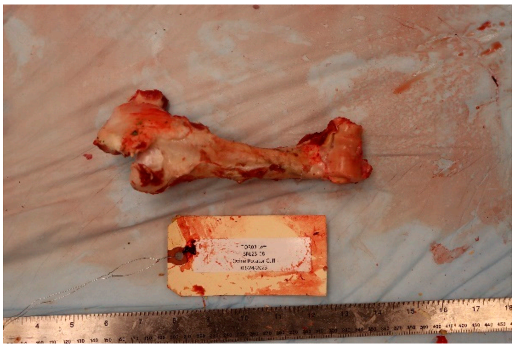</td><td>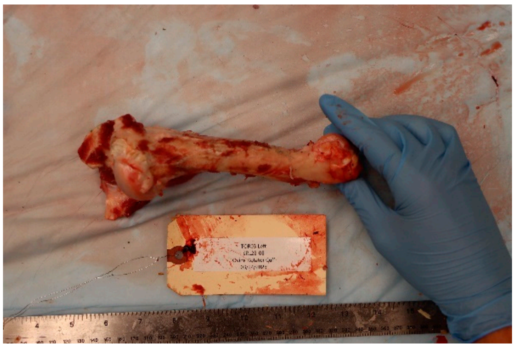</td></tr>
<tr><th colspan="2">Axial</th></tr>
<tr><td colspan="2">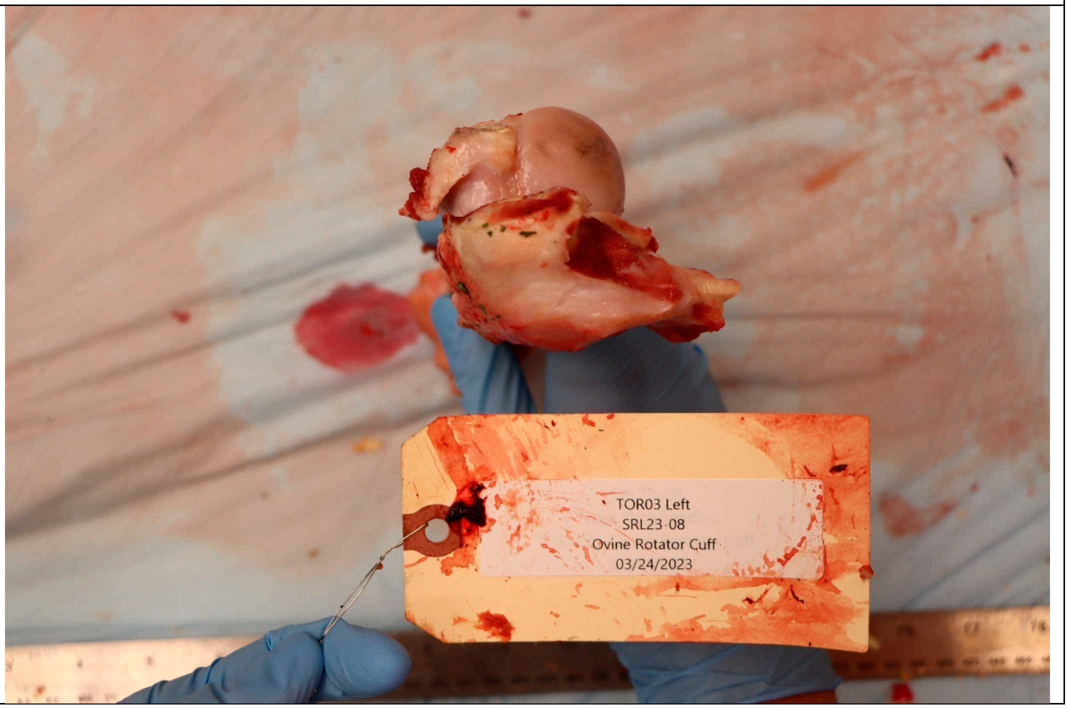
</td></tr>
</table>

<table>
<tr><th>Specimen Info</th><th>(View)</th><th>(View)</th></tr>
<tr><td rowspan="5">Sample ID:
TOR03 R

Group:
Treatment

Timepoint:
8 weeks</td><td>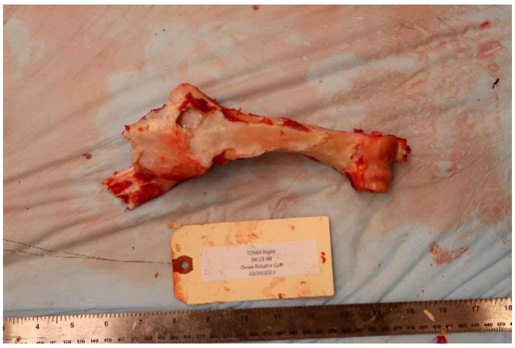</td><td>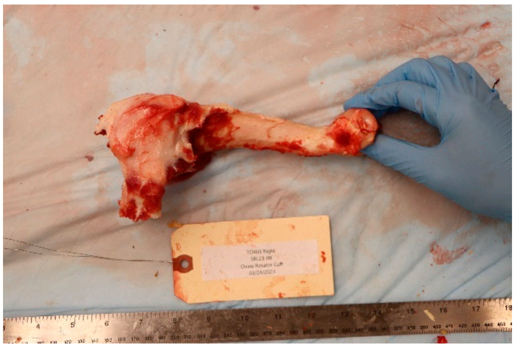</td></tr>
<tr><th>(View)</th><th>(View)</th></tr>
<tr><td>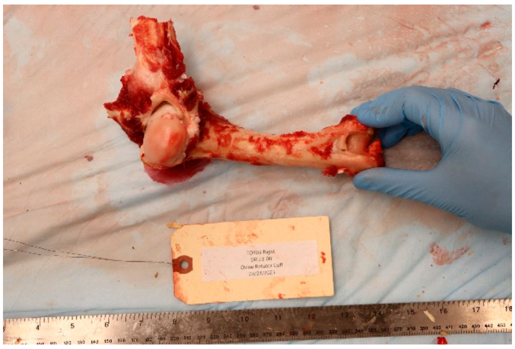</td><td>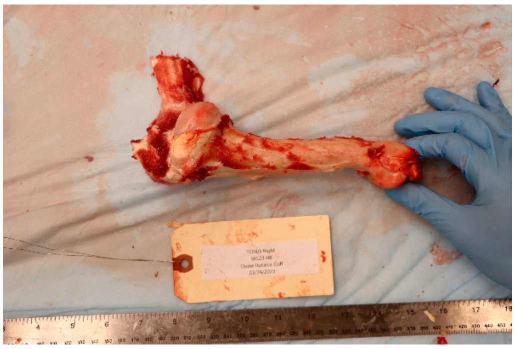</td></tr>
<tr><th colspan="2">(View)</th></tr>
<tr><td colspan="2">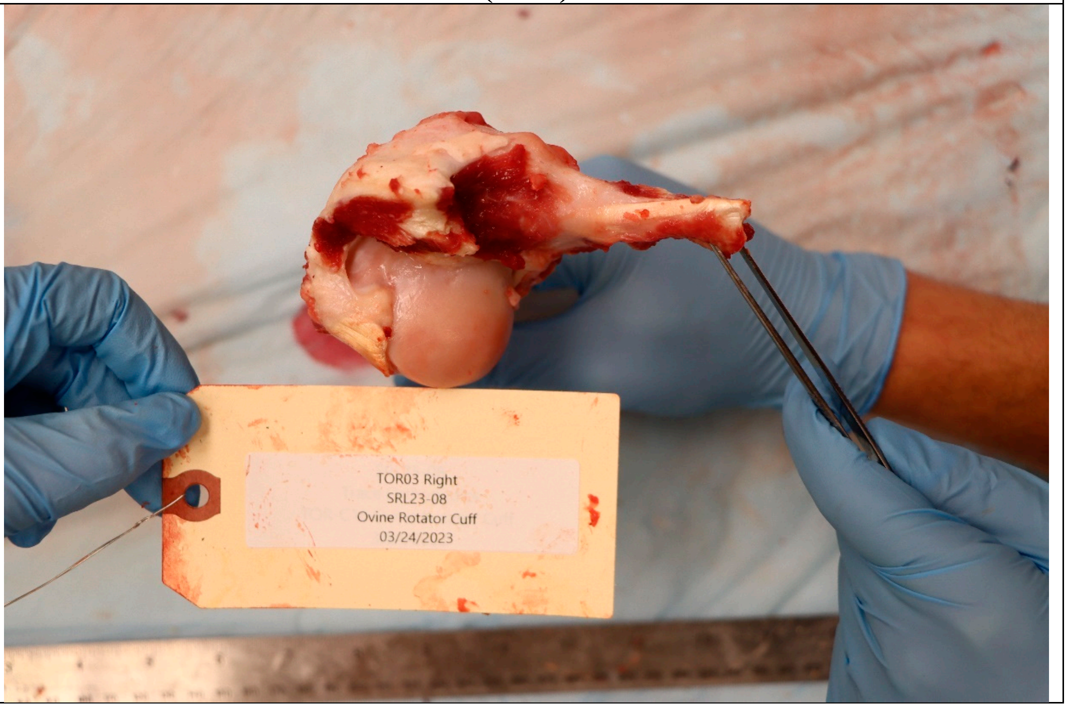
</td></tr>
</table>

Supplementary File D

APPENDIX D: In Vivo Radiographs

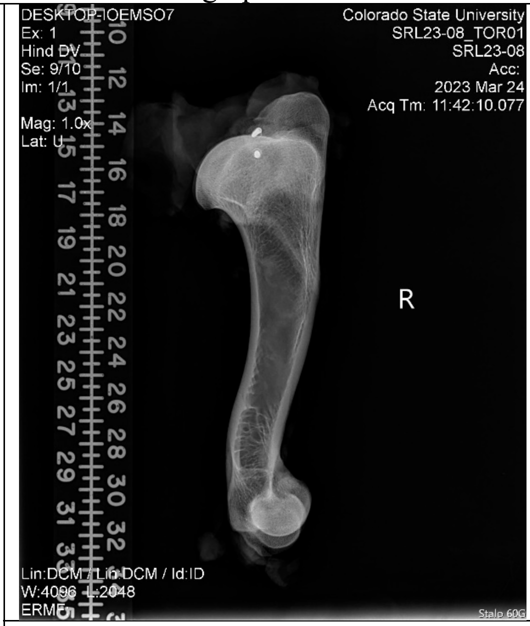

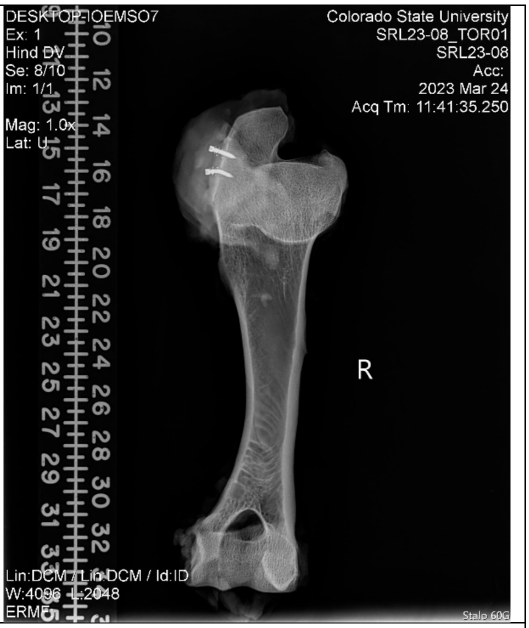

Sample ID:
TOR01

Group:
Control

Timepoint:
8 weeks

Left Shoulder: M/L

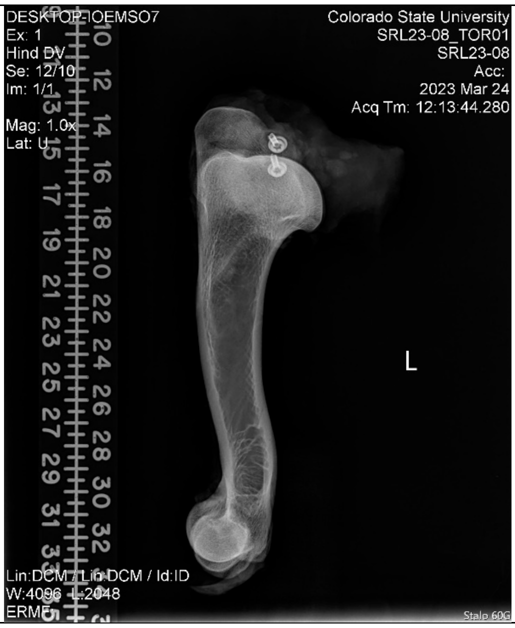

Left Shoulder: D/V

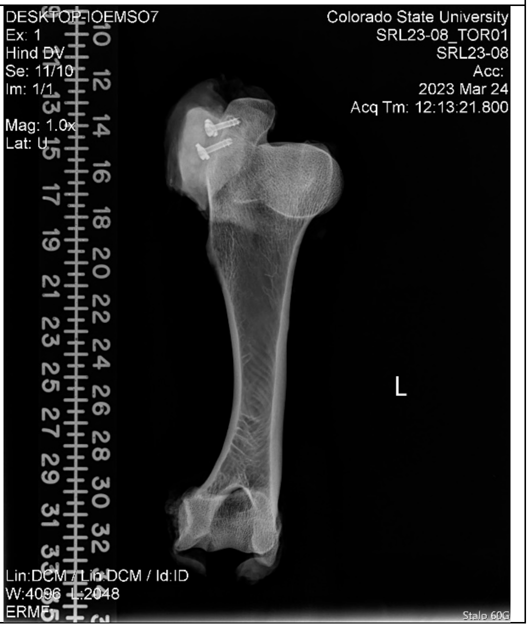

<table>
<tr><th>Specimen Info</th><th>Right Shoulder: M/L</th><th>Right Shoulder: D/V</th></tr>
<tr><td rowspan="3">Sample ID:
TOR02

Group:
Control

Timepoint:
8 weeks</td>
<td>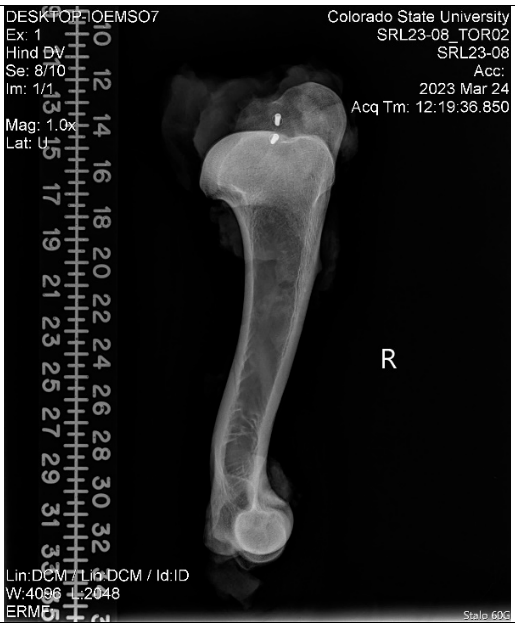
</td>
<td>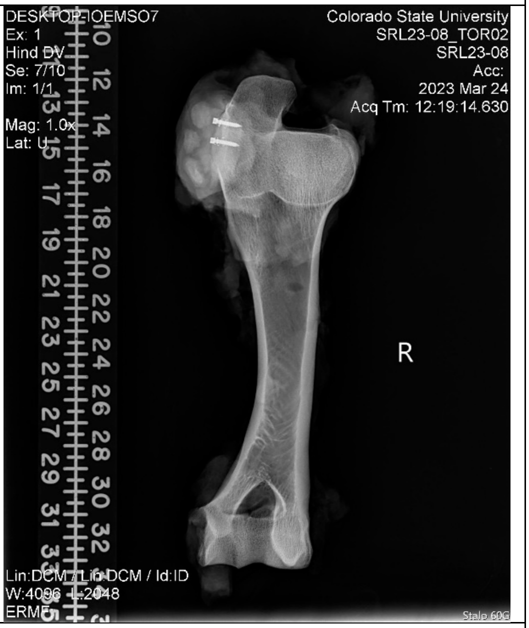
</td></tr>
<tr><th>Left Shoulder: M/L</th><th>Left Shoulder: D/V</th></tr>
<tr><td>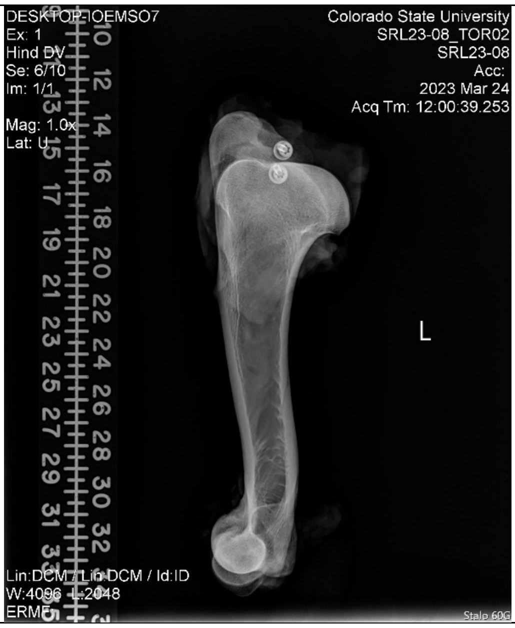
</td>
<td>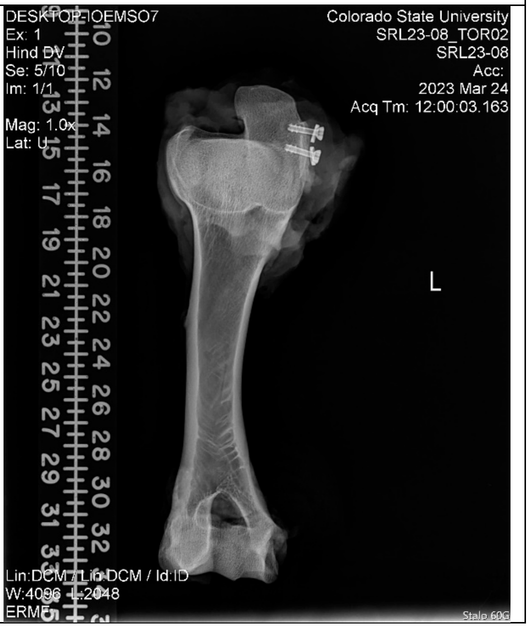
</td></tr>
</table>
